# Antioxidant and lipid supplementation improve the development of photoreceptor outer segments in pluripotent stem cell-derived retinal organoids

**DOI:** 10.1016/j.stemcr.2022.02.019

**Published:** 2022-03-24

**Authors:** Emma L. West, Paromita Majumder, Arifa Naeem, Milan Fernando, Michelle O'Hara-Wright, Emily Lanning, Magdalena Kloc, Joana Ribeiro, Patrick Ovando-Roche, Ian O. Shum, Neeraj Jumbu, Robert Sampson, Matt Hayes, James W.B. Bainbridge, Anastasios Georgiadis, Alexander J. Smith, Anai Gonzalez-Cordero, Robin R. Ali

**Affiliations:** 1 UCL Institute of Ophthalmology, 11-43 Bath Street, London EC1V 9EL, UK; 2 NIHR Biomedical Research Centre at Moorfields Eye Hospital NHS Foundation Trust and UCL Institute of Ophthalmology, City Road, London EC1V 2PD, UK; 3 Kellogg Eye Center, University of Michigan, 1000 Wall Street, Ann Arbor, MI 48105, USA

**Keywords:** retinal organoids, outer segments, disease modeling, induced pluripotent stem cells, RPGR, gene therapy, photoreceptors, retinitis pigmentosa, degeneration, docosahexaenoic acid

## Abstract

The generation of retinal organoids from human pluripotent stem cells (hPSC) is now a well-established process that in part recapitulates retinal development. However, hPSC-derived photoreceptors that exhibit well-organized outer segment structures have yet to be observed. To facilitate improved inherited retinal disease modeling, we determined conditions that would support outer segment development in maturing hPSC-derived photoreceptors. We established that the use of antioxidants and BSA-bound fatty acids promotes the formation of membranous outer segment-like structures. Using new protocols for hPSC-derived retinal organoid culture, we demonstrated improved outer segment formation for both rod and cone photoreceptors, including organized stacked discs. Using these enhanced conditions to generate iPSC-derived retinal organoids from patients with X-linked retinitis pigmentosa, we established robust cellular phenotypes that could be ameliorated following adeno-associated viral vector-mediated gene augmentation. These findings should aid both disease modeling and the development of therapeutic approaches for the treatment of photoreceptor disorders.

## Highlights

• Antioxidants and lipids are required for the formation of organized outer segments
• Both rod and cone hPSC-derived photoreceptors generate well-formed outer segments
• Improved conditions provide a robust model of X-linked retinitis pigmentosa type 3
• Enhanced segment formation permits the evaluation of therapeutic interventions

## Graphical abstract

**Figure:**
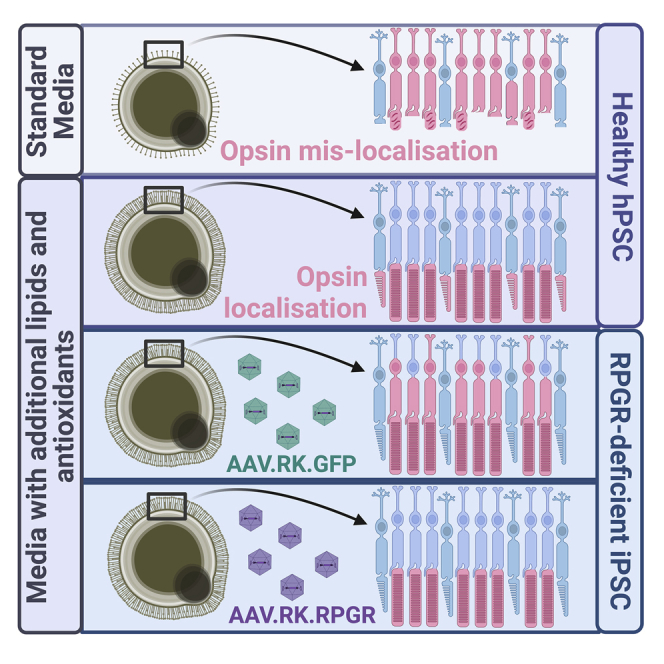

## Teaser

In this article, Ali and colleagues demonstrate the generation of hPSC-derived photoreceptors, bearing organized outer segments. Using these enhanced conditions, they report robust cellular phenotypes in an iPSC-based model of X-linked retinitis pigmentosa, which were ameliorated with gene supplementation. The findings presented will aid disease modeling of human photoreceptor-specific disorders and the development of novel therapeutic approaches.

## Introduction

Inherited retinal degenerations caused by mutations in photoreceptor-specific genes result in blindness for millions of people worldwide, with limited treatment options currently available. A fundamental requirement for the development of novel retinal therapies is the establishment of robust pre-clinical models in which disease mechanisms can be elucidated and new therapies tested. One approach is the use of human pluripotent stem cells (hPSCs), which can be differentiated toward retinal lineages. Since the initial demonstration of ESC-derived optic cups that develop into layered retinal tissue, many groups have devised protocols to generate 3D retinal structures, commonly referred to as retinal organoids (reviewed in O'Hara-Wright and Cordero, 2020). In turn, this has led to an increase in disease modeling using induced pluripotent stem cells (iPSCs) derived from patients carrying photoreceptor-specific mutations (Parfitt et al., 2016; Deng et al., 2018; Megaw et al., 2017; Lane et al., 2020; Gao et al., 2020; Lukovic et al., 2020). However, a remaining limitation is the incomplete maturation of hPSC-derived photoreceptors *in vitro*. To date, only a few rudimentary outer segment (OS)-like structures have been observed by ultrastructural analysis, following extensive long-term culture (Lukovic et al., 2020; Wahlin et al., 2017; Gonzalez-Cordero et al., 2017; Ovando-Roche et al., 2018). The photoreceptor OS is a specialized sensory cilium, formed of stacked membranous discs that are constantly renewed and contain the photoresponsive opsins (Young, 1969). Therefore, improved formation of hPSC-derived photoreceptors bearing OSs is essential for effective disease modeling, as these structures are vital for photoreceptor function.

The increased metabolic activity of mature photoreceptors is maintained *in vivo* by the high rate of choroidal blood flow and the supportive function of the retinal pigment epithelial (RPE) cells (Boulton and Dayhaw-Barker, 2001; Strauss, 2005). RPE cells phagocytose shed OSs, recycling both visual pigments and essential fatty acids, the two major components of the disc membranes (Rice et al., 2015; Bazan et al., 1992). By considering the high concentrations of essential nutrients maintained in the outer retina, we developed an optimized medium for the long-term culture of maturing photoreceptors. We also investigated supplementation with docosahexaenoic acid (DHA), the predominant long chain poly-unsaturated fatty acid (LC-PUFA) present in the retina, which is essential for correct OS disc morphology and optimal visual function (Shindou et al., 2017). In addition, we examined whether our enhanced culture conditions might allow improved retinal disease modeling. We therefore generated iPSC lines from patients with X-linked retinitis pigmentosa type 3 (XLRP3) caused by pathogenic mutations in the retinitis pigmentosa GTPase regulator (*RPGR*) gene. *RPGR* mutations result in defective photoreceptor cilial function and cause a particularly severe type of RP with an early onset of disease in childhood and relatively rapid progression that leads to severe visual impairment by the third to fourth decade (Tee et al., 2016). We were able to observe cellular defects in XLRP3 hPSC-derived retinal organoids that subsequently allowed us to evaluate whether a shortened *RPGR* transgene was able to rescue function in human photoreceptors.

## Results

### Generation of mouse ESC-derived photoreceptor cells bearing outer segment-like structures

To promote the formation of photoreceptor OSs, not previously observed in late-stage mouse embryonic stem cell (mESC)-derived retinal cultures, we designed an enhanced serum-free medium, herein referred to as ALT. To determine the importance of individual components of ALT medium, we also tested an antioxidant-rich medium, hereafter referred to as AOX medium, in addition to our original retinal maturation medium (RMM); see Table S1 for media composition. In parallel, we supplemented with 50 μM DHA pre-complexed with fatty acid-free BSA (referred to as +DHA). BSA alone was used as a control (+BSA). The differentiation of mESC-derived embryoid bodies (EBs) containing retinal regions was established as previously described, with retinal cultures maintained in long-term media from day 21 onward (Kruczek et al., 2017).

**Figure 1:**
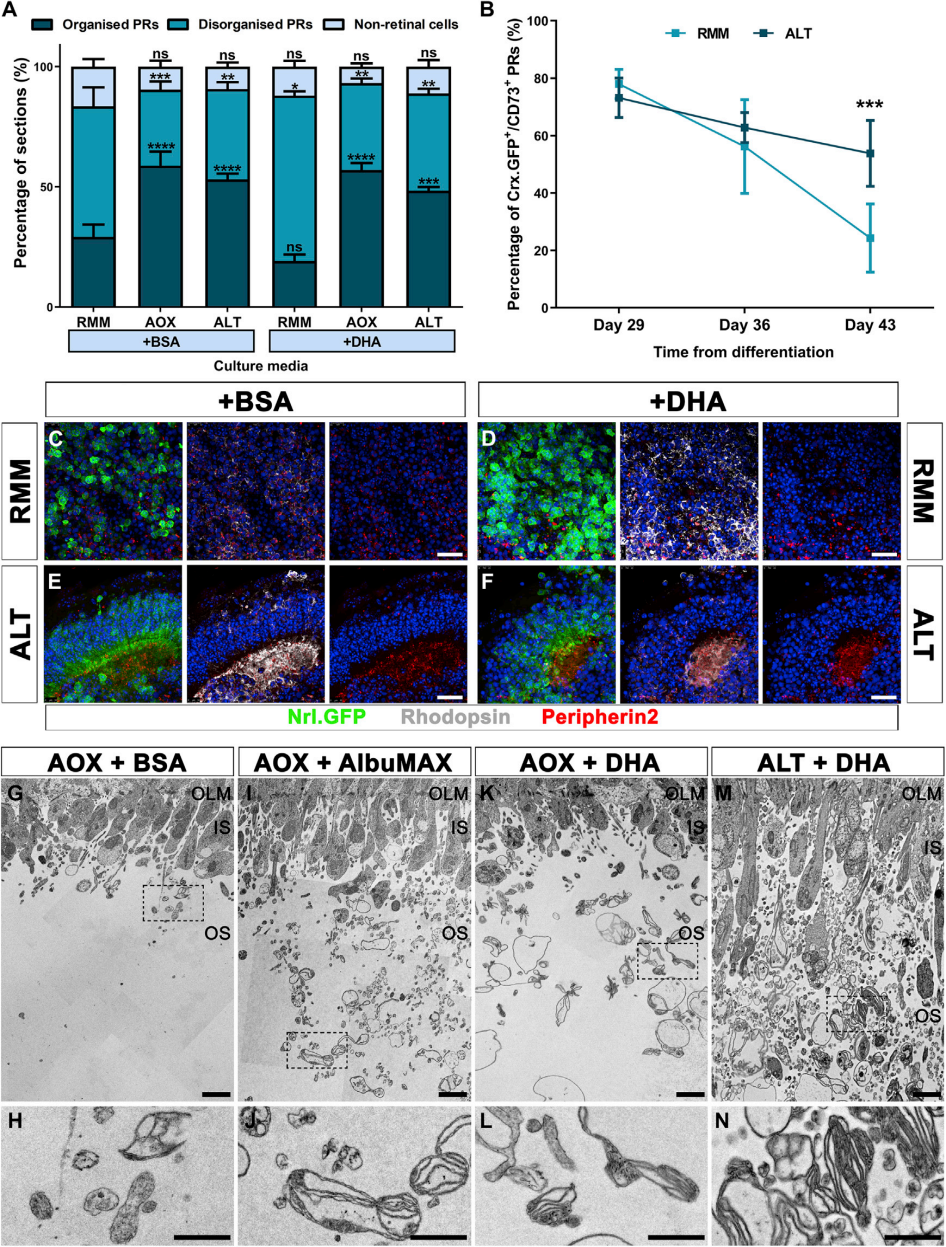
Improved long-term culture of mouse ESC-derived photoreceptors (A) Histogram showing the percentage of classified sections (non-retinal, organized, and disorganized PRs; see Figure S1 and supplemental experimental procedures for criteria) when cultured under various conditions (±SEM; ∗p < 0.05, ∗∗p < 0.01, ∗∗∗p < 0.001, ∗∗∗∗p < 0.0001; n ≥ 12 sections, N ≥ 3 experiments). (B) Line graph showing the percentage of rod (Crx.GFP^+^CD73^+^) PRs present over time in RMM or ALT media (±SD; ∗∗∗p < 0.001; n = 24 pooled EBs, N ≥ 3 experiments). (C–F) Representative images of mESC-derived retinal regions (Nrl.GFP line, green rod PRs) at day 42, maintained in either RMM or ALT media, with +BSA or +DHA and stained for rhodopsin (gray) and peripherin2 (red). (G–N) TEM micrographs showing retinal regions maintained in different culture conditions. Inset regions shown at higher magnification (H, J, L, and N). Nuclei were stained with DAPI (blue). Scale bars, 1μm (H, J, L, and N), 2μm (G, I, K, and M), and 10μm (C–F). IS, inner segment region; OLM, outer limiting membrane; OS, outer segment region; PRs, photoreceptors.

The first sign of mESC-derived photoreceptor cell loss after day 30 is the disorganization of the outer nuclear layer (ONL)-like structures within the EBs. We therefore examined day 34 cryosections, according to set criteria (Figures S1A–S1H; see supplemental experimental procedures) and determined the percentage of sections with organized, disorganized, or no photoreceptors (non-retinal cells) following culture with the various media (Figure 1A). A significantly greater percentage of sections containing organized photoreceptors was observed with AOX and ALT media, compared with those maintained in RMM with BSA (Figure 1A; 59 ± 10% and 53 ± 6% versus 29 ± 12% of sections, respectively; mean ± SEM, p < 0.0001; two-way ANOVA with Dunnett's multiple comparisons test; n ≥ 12 sections, N ≥ 3 differentiations). This suggests that the increased antioxidants, present in both AOX and ALT media, supported the maintenance of photoreceptor organization and enhanced survival at this later developmental timepoint. A similar result was observed for AOX and ALT media with DHA supplementation, compared with the RMM +BSA (Figure 1A, 57 ± 5% and 48 ± 4% versus 29 ± 12% of sections, respectively; mean ± SEM, p < 0.001). However, significantly more sections contained disorganized photoreceptors in RMM +DHA, suggesting a detrimental effect of increased lipid concentrations without additional antioxidants (Figure 1A; 69 ± 4% versus 54 ± 18% of sections, respectively; mean ± SEM, p < 0.05). No significant differences were found in the percentage of sections containing non-retinal cells for any of the media conditions (Figure 1A; mean ± SEM, p > 0.05).

To determine if there was increased survival of photoreceptors over time, we used flow cytometry to analyze the percentage of rod photoreceptors (Crx.GFP^+^CD73^+^ cells), using a Crx.GFP mESC line (Figures 1B and S1I). At day 29, no significant difference in the percentage of rods grown in either RMM or ALT media only was observed, confirming differentiation was unaffected (Figure 1B; 73 ± 7% versus 78 ± 5% Crx.GFP^+^CD73^+^ cells in ALT and RMM, respectively; p > 0.05; two-way ANOVA with Sidak's MCT; n = 24 pooled EBs, N ≥ 3 experiments). At day 36 and 43, the percentage of rods was greatly reduced when cultured in standard RMM. Despite a slight reduction over time, use of ALT medium resulted in a significantly higher percentage of rods being preserved at day 43 (Figure 1B; 54 ± 12% versus 24 ± 12% Crx.GFP^+^CD73^+^ cells, respectively; p < 0.001). These results therefore support the use of this enriched base medium for extended preservation of mESC-derived photoreceptors at late stages of culture.

To look in more detail at the morphology of photoreceptors, cryosections were stained for phototransduction proteins rhodopsin and peripherin, which localize to the OS in the adult mouse retina (Figures 1C–1F and S2). As previously observed, rhodopsin and peripherin staining was present in the segment region of the photoreceptors under standard conditions (RMM +BSA), with rhodopsin also present in the ONL-like layer (Figures S2A and S2G and Gonzalez-Cordero et al., 2013). Similar results were observed for all other media conditions (Figures S2C, S2E, S2I, and S2K). However, slightly less rhodopsin was noted in the ONL-like layer for both AOX and ALT media conditions with lipid-bound BSA, for all mESC lines examined (Figures S2D, S2F, S2J, and S2L and data not shown). This could be clearly observed at day 42, whereby rhodopsin was discretely localized to the segment region of ALT-grown photoreceptors; in contrast, no organized photoreceptor regions remained under standard conditions, as observed in previous studies (Figures 1C–1E, respectively and Gonzalez-Cordero et al., 2013). This rhodopsin localization is reminiscent of late postnatal (>P12) mouse photoreceptors, a developmental stage that until now had not been possible to investigate in 3D mESC-derived retinal cultures. Further testing of individual components in AOX medium revealed no significant differences in photoreceptor organization, compared with the AOX +BSA control (Figures S2M–S2Q, mean ± SEM, p > 0.05; two-way ANOVA with Dunnett's MCT; n ≥ 12 sections, N ≥ 3 differentiations).

To investigate the formation of OS-like structures further, transmission electron microscopy (TEM) was used to examine day 34 mESC-derived photoreceptors (Figures 1G–1N). Despite preservation of photoreceptor organization with AOX +BSA medium, there was little indication of OS-like structures at the ultrastructural level, similar to previous observations using standard conditions (Figures 1G and 1H) (Gonzalez-Cordero et al., 2013). In contrast, numerous membranous structures were observed with the addition of lipid-rich BSA (AlbuMAX) and BSA-bound DHA (Figures 1I and 1L, respectively). Likewise, membranous structures were abundant in ALT +DHA retinal cultures, with many structures containing numerous internal foldings, reminiscent of nascent OSs (Figures 1M and 1N). These findings demonstrate the importance of additional antioxidants to maintain photoreceptors in long-term mESC-derived retinal cultures, as well as the requirement of BSA-bound lipids for the development of OS-like structures.

### Efficient generation of human PSC-derived photoreceptors bearing outer segment-like structures

**Figure 2:**
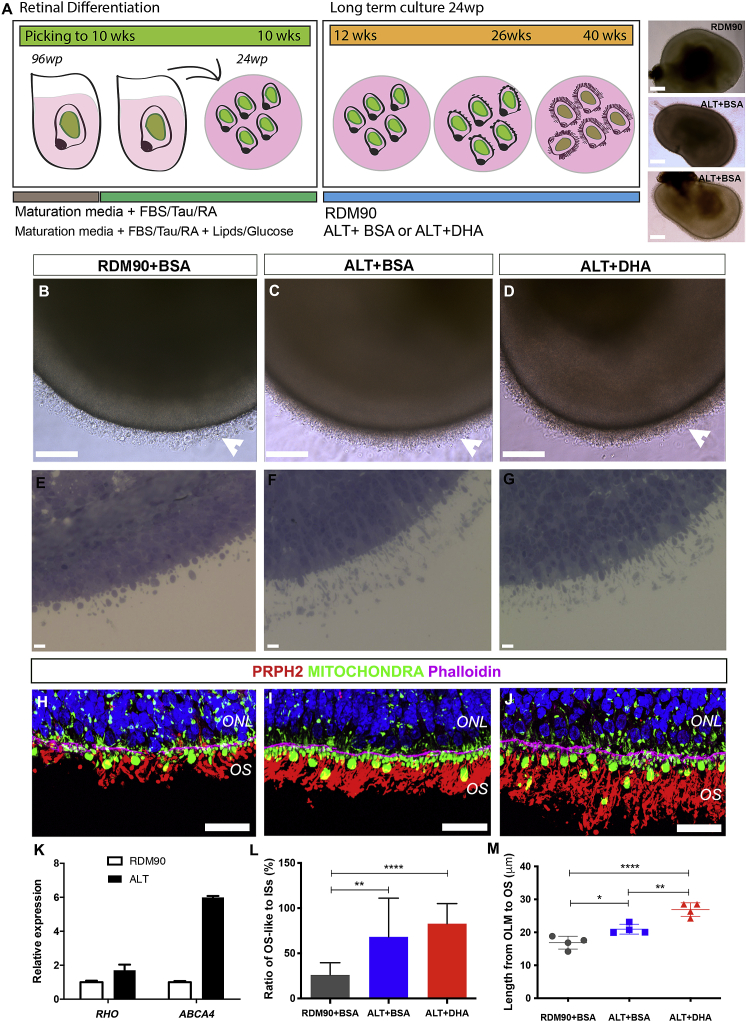
Efficient differentiation of outer segment-bearing photoreceptors in 26-week hESC-derived retinal organoids (A) Schematic of retinal differentiation protocol with bright field images of retinal organoids. (B–D) Representative bright field images showing brush borders (white arrowheads). (E–G) Semithin images of brush border region. (H–J) Retinal neuroepithelium showing phalloidin positive OLM (pink), mitochondria-rich ISs (green), and elongated PRPH2+ OSs (red). (K) RT-qPCR analysis showing the relative expression of *RHO* and *ABCA4* in RDM90 and ALT media (±SEM; n = 15 pooled NRVs, N = 3 experiments). (L) Histogram showing the ratio of OS-like to IS structures as a percentage (±SD; ∗∗p < 0.01, ∗∗∗∗p < 0.0001; n = 30 images, N = 3 experiments). (M) Graph showing the length of segment structures for all conditions (±SD; ∗p < 0.05, ∗∗p < 0.01, ∗∗∗∗p < 0.0001; n = 40 images, N = 4 experiments). Nuclei were stained with DAPI (blue). Scale bars, 20 μm (E–G), 25 μm (H–J), 50 μm (B–D), 70 μm (A). OLM, outer limiting membrane; IS, inner segment; ONL, outer nuclear layer; OS, outer segment.

Having established the benefits of ALT medium for mESC-derived photoreceptors, we next sought to investigate its use for hPSC-derived retinal organoids. A summary of our standard and optimized protocol is shown schematically in Figure 2A. Briefly, hPSC-derived neuroretinal vesicles (NRVs) were isolated from confluent cultures at 3–5 weeks and grown in suspension for up to 10 weeks to form retinal organoids, in accordance with our original protocol (Figure 2A, see methods for detailed protocol). Organoids were further cultured from 12 weeks with either our standard RDM90 or ALT medium, supplemented with either BSA-bound DHA (+DHA) or fatty acid-free BSA (+BSA) as the control. As previously described (Gonzalez-Cordero et al., 2017), using our standard medium supplemented with BSA (RDM90 +BSA), bright field images of retinal organoids revealed a semi-translucent laminated neuroepithelium with brush-like protrusions, herein referred to as a brush border (Figure 2B, white arrowhead). However, a more protuberant brush border was observed in retinal organoids cultured with ALT medium (Figures 2C and 2D, white arrowheads). No differences between ALT +BSA and ALT +DHA were evident, and RDM90 supplemented with DHA was not sufficient to induce the dense brush border observed with ALT medium (Figures S3A–S3F). To examine the brush border in more detail we analyzed semithin sections, which verified this region comprised segment-like structures protruding from the neuroepithelial layer (Figures 2E–2G). Photoreceptor OS formation was further confirmed in all conditions by the presence of PERIPHERIN-2 positive (Figures 2H–2J; PRPH2+; red) structures observed apically to the mitochondria-rich inner segments (Figures 2H–2J; MITOCHONDRIA+; green) and the outer limiting membrane (OLM), delineated by phalloidin (Figures 2H–2J; Phalloidin+; magenta). In addition, immunohistochemical analysis demonstrated the presence of ABCA4, a transmembrane phospholipid-transporting ATPase present in the membranes of OSs *in vivo*, localized apically to ESPIN, a connecting cilium (CC) marker (Figures S3G and S3H). Transcriptional analyses also confirmed the increased expression of phototransduction components *RHO* and *ABCA4* for organoids maintained in ALT medium, compared with RDM90 (Figure 2K).

To determine if the dense brush borders observed were due to the increased efficiency of OS-like structure formation, the ratio of OS-like to inner segment structures was quantified and expressed as a percentage. The percentage of inner segments that had OS-like structures was significantly greater for both ALT +BSA (68 ± 43%) and ALT +DHA (83 ± 22%) compared with RDM90 +BSA (26 ± 14%) cultures (Figure 2L; p < 0.01; Kruskal-Wallis with Dunn's MCT; n = 30 images, N = 3 differentiations). In addition, the length of the segments, from the Phalloidin+ OLM to the distal edge of the PRPH2+ OS-like structures, was measured. Significantly longer segments were observed for both ALT conditions (21 ± 2 μm and 27 ± 2 μm for +BSA and +DHA, respectively), compared with RDM90 +BSA (17 ± 2 μm) (Figure 2M; p < 0.05; ANOVA with Tukey's MCT; n = 40 images, N = 4 differentiations). Combined, these results suggest the improved formation of photoreceptor OS-like structures in retinal organoids at 26 weeks, when cultured with ALT medium.

### Improved development of hPSC-derived photoreceptor outer segment-like structures with long-term culture in ALT medium

**Figure 3:**
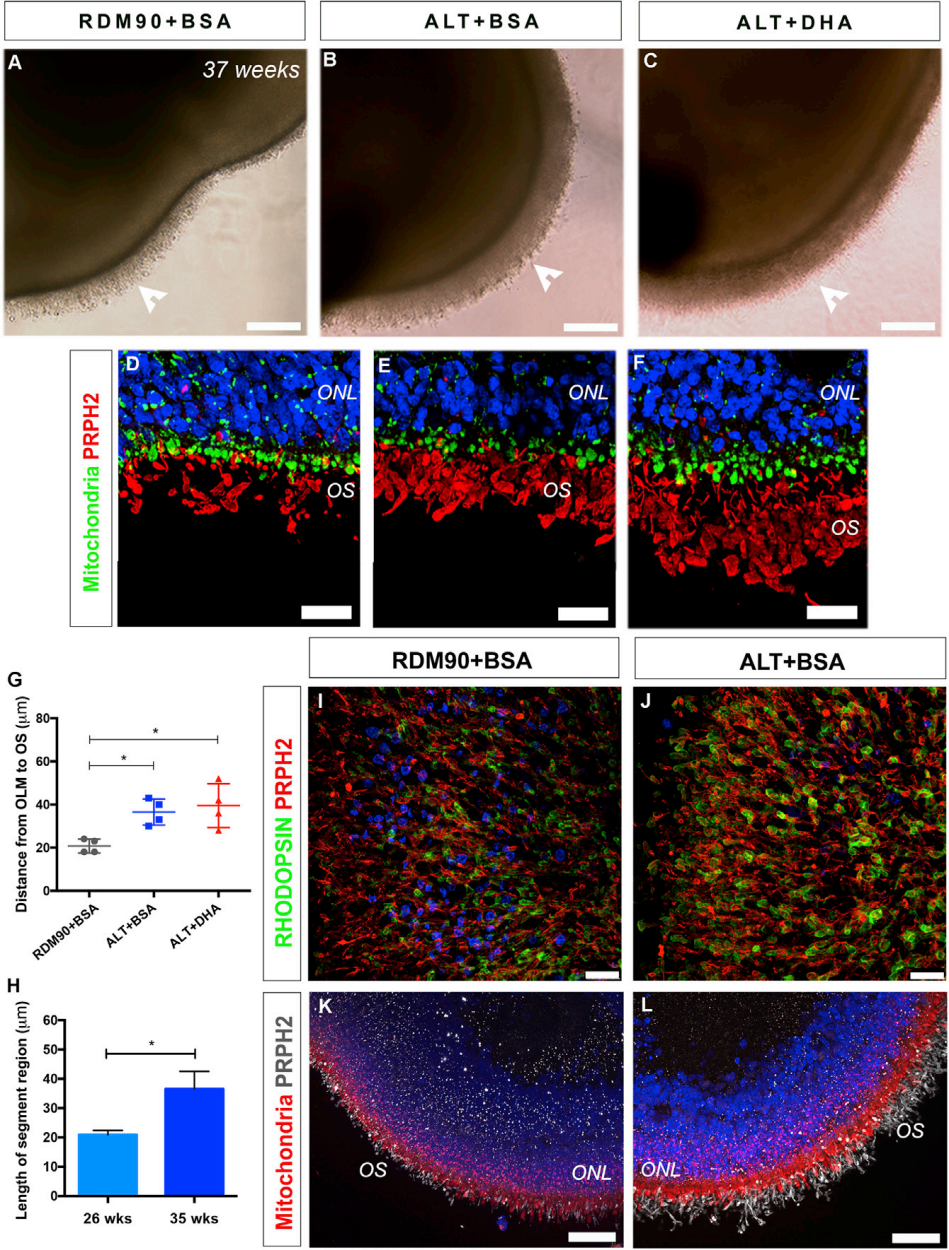
Maintained formation of outer segments by photoreceptors at late stages of culture (A–C) Representative bright field images of 37-week retinal organoids showing brush borders (white arrowheads). (D–F) Retinal organoid neuroepithelium showing mitochondria-rich ISs (green) and elongated PHPR2+ OSs (red). (G) Graph showing the length of segment structures maintained in all media conditions (±SD; ∗p < 0.05; n = 40 images, N = 4 experiments). (H) Graph showing the segment length in 26- and 35-week ALT cultures (±SD; ∗p < 0.05; n = 40 images, N = 4 experiments). (I and J) 3D view of a retinal organoid showing the distribution of RHODOPSIN+ (green) and PHPR2+ (red) photoreceptor OSs. (K and L). Cross-sectional image, showing the mitochondria-rich ISs (red) and PHPR2+ OSs (gray). Nuclei were stained with DAPI (blue). Scale bars, 25 μm (D, F, I, and J), 50 μm (K and L). ONL, outer nuclear layer; IS, inner segment; OS, outer segment.

To assess if the enhanced brush borders could be maintained, retinal organoids were cultured for up to 37 weeks. A clear difference in gross morphology of the brush border could be observed in both ALT conditions, compared with organoids cultured under standard conditions (Figures 3A–3C). While the inner segments packed with mitochondria and PHPR2+ OS-like structures could be readily identified in all conditions (Figures 3D–3F), significantly longer segment structures were observed for both ALT conditions, compared with RDM90 +BSA (Figure 3G, 37 ± 6 μm +BSA and 40 ± 10 μm +DHA versus 21 ± 3 μm, respectively; p < 0.05; ANOVA with Tukey's MCT; n = 40 images, N = 4 differentiations). In addition, the length of the segment region significantly increased from 26 to 35 weeks in ALT-cultured organoids (Figure 3H; 21 ± 2 μm versus 37 ± 6 μm, respectively; p = 0.029; two-tailed Mann-Whitney test; n = 40 images, N = 4 differentiations). This contrasted with the minimal increase in segment length for RDM90 +BSA cultured organoids over the same period (17 ± 2 μm versus 21 ± 3 μm, respectively). To determine the widespread coverage of RHODOPSIN+/PHPR2+ OS-like protrusions, we examined whole retinal organoids (Figures 3I and 3J). While the use of either medium resulted in the presence of OS-like structures at 37 weeks, the DAPI-positive nuclei (blue) were visible through the brush border under standard conditions, but not in ALT-cultured organoids (Figures 3I and 3J, respectively). This improved coverage was also evident when the organoids were analyzed in cross-section, with defined inner segment (Mitochondria+; red) and OS-like (PHPR2+; gray) structures more apparent in ALT-cultured organoids (Figures 3K and 3L). The results described here were performed using H9 hESC-derived retinal organoids; however, enhanced brush borders were observed in all hPSC lines tested (Figures S3I–S3P; N > 5). These findings support the improved formation and continued development of OS-like structures in hPSC-derived photoreceptors, cultured long term in ALT medium. In addition, supplementation with DHA did not result in any significant improvement, as assessed by light microscopy, beyond that observed with ALT +BSA, at late stages of development.

### Improved formation of hPSC-derived cone photoreceptor outer segment-like structures

**Figure 4:**
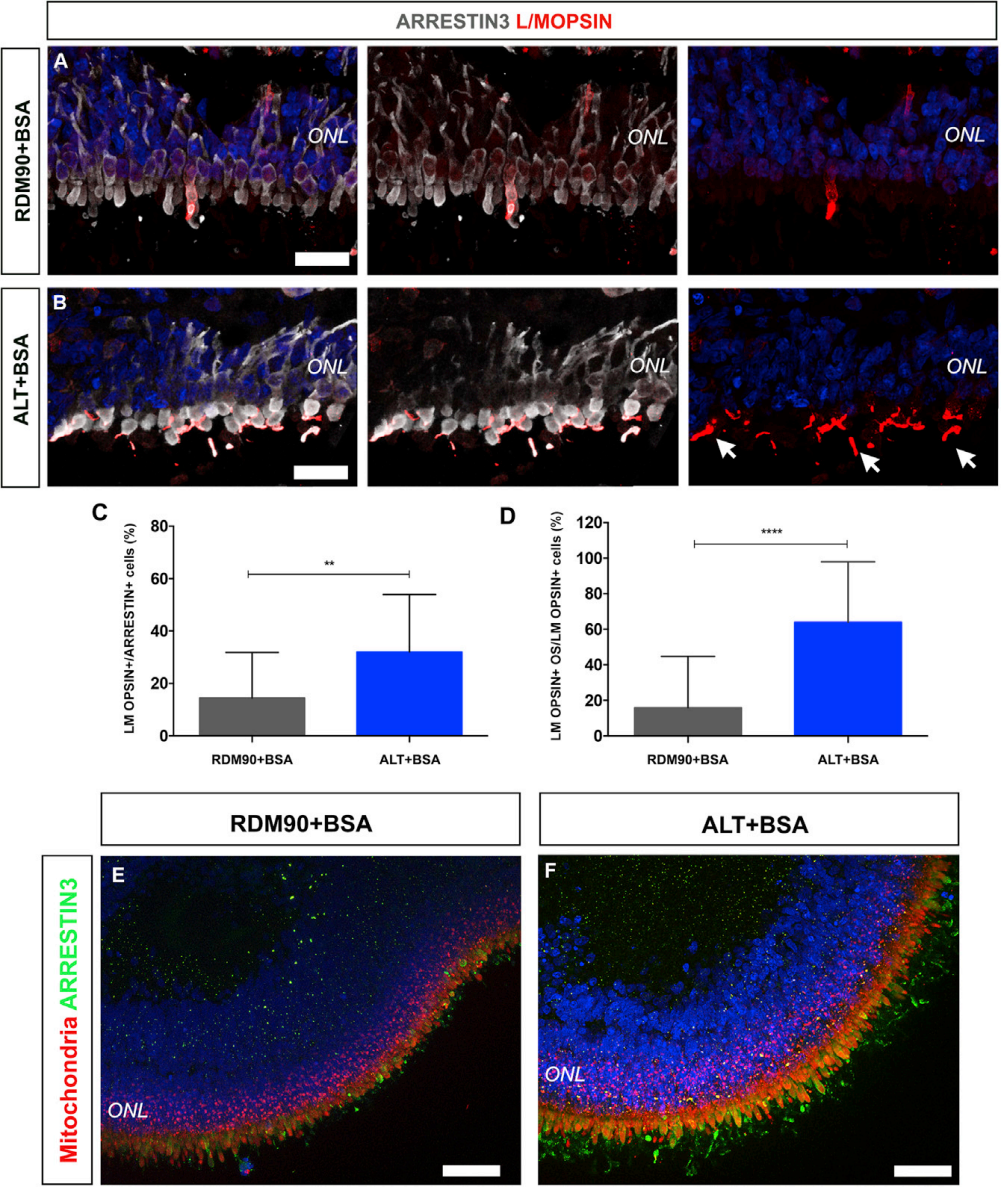
Development of outer segment-bearing cone photoreceptors (A and B) Representative images showing ARRESTIN3+ and L/MOPSIN+ cone photoreceptors cultured in RDM90 (A) and ALT medium (B). (B) In ALT medium L/MOPSIN staining is localized to outer segments (white arrows). (C) Histogram showing the percentage of ARRESTIN3+ cones that were also LM OPSIN+ (±SD; ∗∗p < 0.01; n = 30 images, N = 3 experiments). (D) Histogram showing the percentage of LM OPSIN+ cones that had LM OPSIN localized to the OS (±SD; ∗∗∗∗p < 0.0001; n = 30 images, N = 3 experiments). (E and F) Cross-sectional image, showing the mitochondria+ ISs (red) and ARRESTIN3+ cone OSs (green). Nuclei were stained with DAPI (blue). Scale bars, 25 μm (A and B), 50 μm (E and F). ONL, outer nuclear layer; OS, outer segment; IS, inner segment.

While previous studies have reported the generation and development of hPSC-derived cone photoreceptors (Zhong et al., 2014; Zhou et al., 2015; Gonzalez-Cordero et al., 2017), the formation of cone OS-like structures has not been described specifically. We therefore sought to establish if ALT medium would improve segment formation in hPSC-derived cone photoreceptors. Cones were present in both media conditions, as demonstrated by cone-specific phototransduction components ARRESTIN3 and L/M OPSIN (Figures 4A and 4B). By 37 weeks a significantly greater percentage of double-positive LM OPSIN+/ARRESTIN3+ cones were observed in ALT +BSA compared with RDM90 +BSA medium (Figure 4C; 32 ± 22% versus 14 ± 17%, respectively; p = 0.0011; two-tailed Mann-Whitney test; n = 30 images, N = 3 differentiations). To determine if this corresponded to improved segment formation, we quantified the percentage of cones showing LM OPSIN+ segment-like structures. While 16 ± 29% of cones demonstrated elongated LM OPSIN+ segment-like structures under standard conditions, this was significantly increased with ALT medium to 64 ± 34% (Figure 4D; p < 0.0001; two-tailed Mann-Whitney test; n = 30 images, N = 3 differentiations). Furthermore, examining whole retinal organoids in cross-section demonstrated the augmented formation of cone OS-like structures (ARRESTIN3+; green), apical to mitochondria-rich inner segments (MITOCHONDRIA+; red), with ALT (Figures 4E and 4F). These results suggest that ALT medium supports enhanced segment formation in cone as well as rod photoreceptor subtypes.

### Ultrastructural analysis of hPSC-derived photoreceptors indicates improved outer segment disc structure following culture in ALT medium

**Figure 5:**
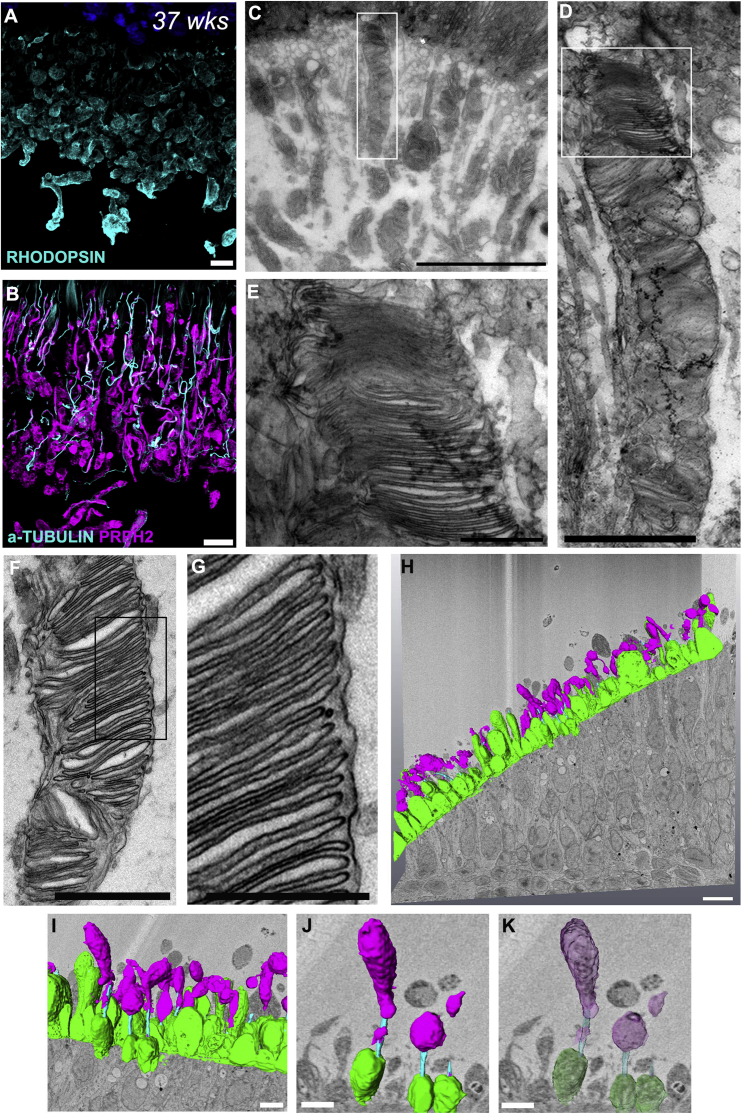
Ultrastructure analysis of photoreceptor cells with improved outer segment formation (A) Immunohistochemical image of RHODOPSIN+ (cyan) OS regions in ALT maintained photoreceptors. (B) Image showing the localization of α-TUBULIN+ axonemes (cyan) and PHPR2+ OSs (pink) in ALT-cultured photoreceptors. (C–E) TEM micrographs of 26-week hPSC-derived photoreceptors cultured in ALT +DHA, showing the abundance of OSs (C). High magnification image of inset from C, showing the morphology of disc membranes (D). High magnification image of inset from D, showing stacked disc membranes (E). (F and G) Transverse TEM section through one OS (F). High magnification image of inset from (F), showing organized and stacked membranous discs (G). (H–K) 3view serial 3D reconstruction of the ONL region of an ALT +DHA cultured retinal organoid. The photoreceptor ISs (green), CC (blue), and OSs (pink) were pseudo-colored. Scale bars, 0.5 μm (E and G), 1 μm (F), 2 μm (D),10 μm (A–C). IS, inner segment; CC, connecting cilium; ONL, outer nuclear layer; OS, outer segment.

The unique structure of photoreceptor OSs, made up of densely packed membranous discs, is crucial for efficient phototransduction. Immunohistochemical analysis of photoreceptors cultured with ALT medium demonstrated elongated axonemes as shown by acetylated-tubulin (cyan), as well as RHODOPSIN- and PHPR2-positive OS-like structures (Figures 5A and 5B, cyan and pink, respectively). To determine the ultrastructure of these extended structures, we examined late-stage retinal organoids by electron microscopy. TEM of photoreceptors maintained in ALT medium confirmed the abundance of structures containing membranous infoldings, reminiscent of OS discs (Figures 5C–5G and S4D–S4K). In contrast, few diffuse and disorganized structures were observed in RDM90 cultures, as described previously (Figures S4A–S4C; Gonzalez-Cordero et al., 2017). The OSs of photoreceptors cultured in ALT medium showed discrete membranous structures, with closely stacked disc-like structures clearly present for photoreceptors cultured in ALT +DHA (Figures 5E–5G and S4G–S4K). To confirm the increased abundance of OSs with disc-like morphology following culture in ALT medium, serial block-face scanning electron microscopy (3View) reconstructions of 150 TEM serial sections were performed for all culture conditions (Figures 5H–5K, S5A–S5D and Video S1). In addition, scanning electron microscopy also confirmed an increase in OS formation, following culture in ALT medium (Figures S5E–S5N).

Video S1. 3view EM movie through the segment region of an ALT grown retinal organoid, related to Figure 5 3view 3D reconstruction of 150 sections with thickness of 100 nm each. 3view sequence of backscatter electron microscopy images of hPSC-derived retinal neuroepithelia showing photoreceptor OS (magenta), CC (blue), and IS (green).

### Generation and characterization of RPGR-deficient iPSC-derived retinal organoids

**Figure 6:**
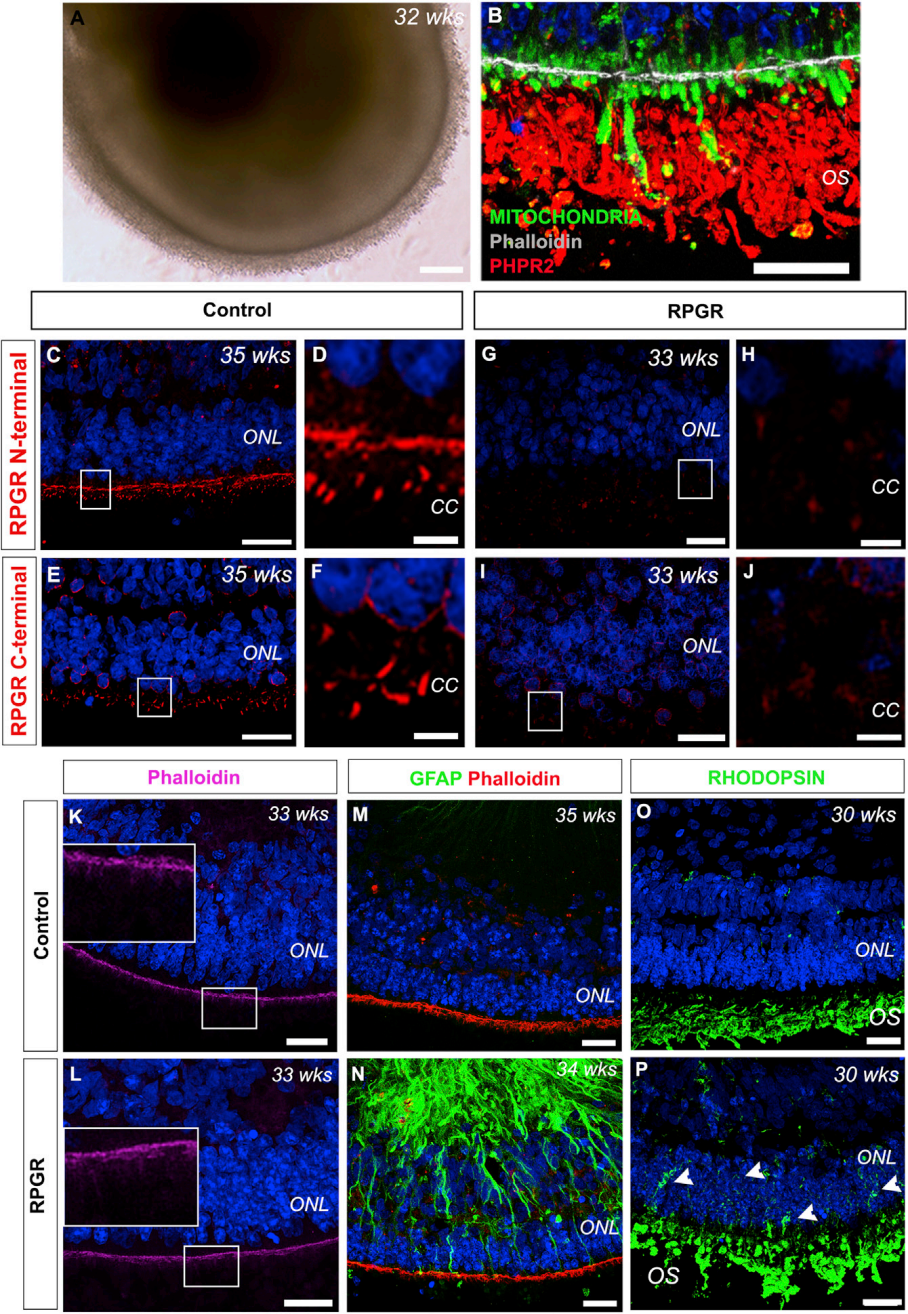
Differentiation and characterization of iPSC-derived RPGR-deficient retinal organoids (A) Bright field image of an RPGR-deficient retinal organoid showing the brush border. (B) Image of RPGR-deficient photoreceptors showing mitochondria-rich ISs (green), phalloidin delineated OLM (gray) and PHPR2+ OSs (red). (C–F) Immunohistochemical analysis with RPGR N-terminal specific antibody (C, D, G, and H) and RPGR C-terminal specific antibody (E, F, I, and J). Images of control retinal organoids, showing typical punctate localization of RPGR (red) to the CC with both antibodies (C–F). (G–J) Images of RPGR-deficient retinal organoids, showing less punctate staining for the constitutive variant of RPGR (G and H) and an absence of staining for the ORF15 isoform (I and J) in the region of the CC. (K and L) Representative images of control and RPGR-deficient organoids showing phalloidin (magenta) localized to the OLM. Inset high magnification panels show no differences in staining pattern. (M and N) Increased GFAP (green) staining in RPGR-deficient neuroepithelia, compared with control. OLM delineated with phalloidin (red) shows no difference. (O and P) Images showing typical RHODOPSIN staining (green) localized to the OSs in both control and RPGR-deficient rod photoreceptors. Mis-localized RHODOPSIN (green) can also be seen in the cell bodies and processes of RPGR-deficient rods (P, white arrowheads). Nuclei were stained with DAPI (blue). Scale bars, 5 μm (D, F, H, and J), 25 μm (B, C, E, G, I, and K–P) and 50 μm (A). IS, inner segment; CC, connecting cilia; ONL, outer nuclear layer; OLM, outer limiting membrane; OS, outer segment.

To determine if these enhanced culture conditions improve disease modeling, we investigated the cellular phenotype present in retinal organoids derived from XLRP3 patients with mutations in the RPGR gene. PBMCs were isolated from three XLRP3 patients, and RPGR-deficient iPSCs were generated, with all three lines demonstrating typical colony morphology, pluripotency markers, and a normal karyotype (Figure S6). Upon retinal differentiation, all lines gave rise to retinal organoids that were further cultured with our enhanced medium, in addition to three healthy control lines (Figure S6W). Bright field images of 32-week RPGR-deficient retinal organoids demonstrated pronounced brush borders, similar to controls (Figures 6A, 3B and 3C, respectively). Immunohistochemistry confirmed the presence of numerous segment structures apical to the OLM, demarcated by phalloidin (gray), with both mitochondria-rich inner segments (green) and PHPR2+ OS-like structures (red) present (Figure 6B). RPGR protein localizes to the CC in photoreceptors and is thought to be involved with protein trafficking to the OS, with RPGR-deficient mouse models demonstrating opsin mis-localization and photoreceptor degeneration (Hong et al., 2001). To evaluate the presence of the two major RPGR isoforms, RPGR (encoded by 19 exons) and RPGR-ORF15 (terminates within intron 15), we analyzed both N- and C-terminal RPGR binding antibodies, respectively. In control organoids, punctate staining was observed apical to the edge of the ONL-like layer, in the region of the CC, for both antibodies (Figures 6C–6F). In contrast, little to no staining was observed in this region for RPGR-deficient photoreceptors, with N- and C-terminal specific antibodies, respectively (Figures 6G–6J). We further analyzed RPGR-deficient retinal organoids for characteristics of the disease phenotype. An increase in actin polymerization, as demonstrated by increased phalloidin staining of the CC, has previously been reported in RPGR-deficient cell lines, RPGR KO mice, and recently in RPGR-deficient iPSC-derived retinal organoids (Gakovic et al., 2011; Megaw et al., 2017). However, we could not detect a difference between our control and RPGR-deficient organoids, with a typical staining pattern of the OLM apparent in both (Figures 6K and 6L, insets and Figures S7A, S7C, and S7D). Reactive gliosis, revealed by increased GFAP expression in Müller glia, is a well-described feature of many retinal degenerations. Such a phenotype has been described in both RPGR KO mice and RPGR iPSC-derived retinal organoids previously (Deng et al., 2018; Megaw et al., 2017). Similarly, GFAP (green) upregulation was evident in RPGR-deficient organoids, when compared with controls (Figures 6M and 6N and S7B and S7C). Finally, as RPGR is thought to be involved in transport across the CC, we examined the localization of RHODOPSIN within both healthy and RPGR-deficient photoreceptors. While in control organoids, RHODOPSIN (green) was discreetly localized to the OSs, in RPGR-deficient photoreceptors, RHODOPSIN was also found throughout the cell, including the cell body and processes (Figures 6O and 6P, arrowheads and S7D). Thus our improved culture conditions enable us to distinguish a clearly defined and clinically relevant cellular phenotype in RPGR-deficient retinal organoids.

### Characterization of RPGR-deficient iPSC-derived retinal organoids following AAV-mediated gene supplementation

**Figure 7:**
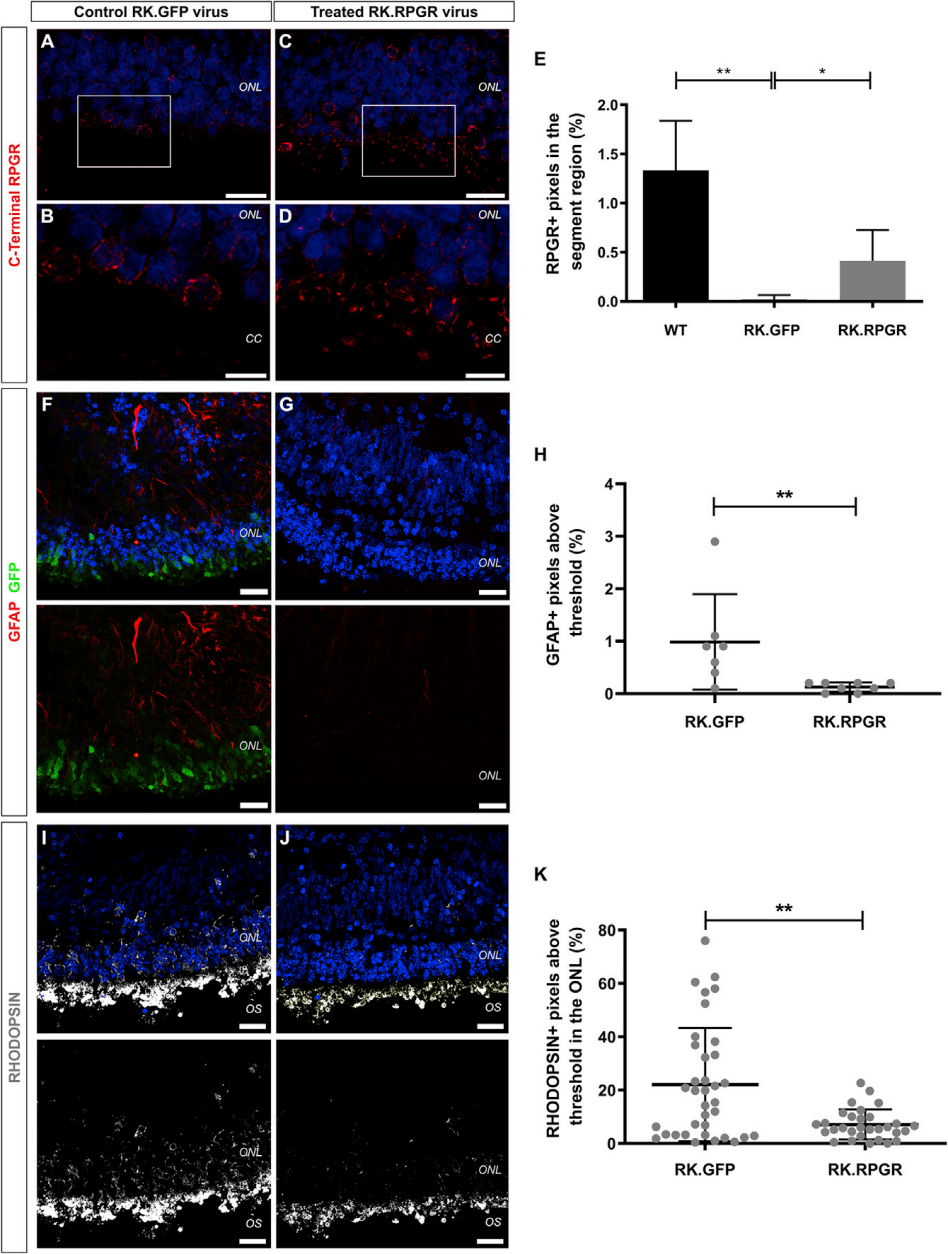
Characterization of RPGR disease phenotype following gene supplementation (A–D) Representative images of iPSC-derived RPGR-deficient retinal organoids, transduced with either RK.GFP or RK.RPGR and stained for the RPGR-ORF15 isoform (red). High magnification images of insets in (A) and (C), highlighting the IS and CC region, are shown in (B) and (D), respectively. (E) Histogram showing the percentage of positive pixels for RPGR-ORF15 staining in the segment region of untreated normal retinal organoids and treated RPGR-deficient retinal organoids (±SD; ∗p < 0.05, ∗∗p < 0.01; n ≥ 12 images, N = 4 experiments). (F and G) Immunohistochemical analysis of GFAP^+^ glial cell processes (red) in RPGR-deficient organoids, treated with RK.GFP or RK.RPGR virus. Transduced GFP^+^ photoreceptors (green) can be seen in the RK.GFP-treated control (F). (H) Graph showing the percentage of GFAP^+^ pixels in treated RPGR-deficient organoids (±SD; ∗∗p < 0.01; n ≥ 7 images, N = 3 experiments). (I and J) Immunohistochemical analysis demonstrating the localization of RHODOPSIN (gray) within rod photoreceptors, following either RK.GFP or RK.RPGR viral transduction. (K) Graph showing the percentage of RHODOPSIN^+^ pixels in the ONL region of treated RPGR-deficient retinal organoids (±SD; ∗∗p < 0.01; n = 30 images, N = 6 experiments). Nuclei were stained with DAPI (blue). Scale bars, 10 μm (B and D), 25 μm (A, C, F, G, I, and J). CC, connecting cilium; IS, inner segment; ONL, outer nuclear layer; OS, outer segment.

To test the efficacy of RPGR gene supplementation, we used an adeno-associated viral (AAV) vector in which a photoreceptor-specific human rhodopsin kinase (RK) promoter (Khani et al., 2007) was used to express a shortened RPGR-ORF15 transgene (AAV7m8.RK.RPGR). The shortened transgene has been used to rescue a mouse model of XLRP3 (Pawlyk et al., 2016) but had not previously been shown to rescue function in human RPGR-deficient photoreceptors. An AAV vector driving a GFP reporter under the control of the same RK promoter was used as a control (AAV7m8.RK.GFP). Vectors were added to cultures between 15 and 18 weeks and retinal organoids analyzed from 22 weeks, with an estimated transduction efficiency of ∼44% of photoreceptors, as determined using the control vector (Figure S7E, 44 ± 11.4% GFP^+^ cells; n = 13 sections, N = 5 ROs). First, we determined the presence of RPGR-ORF15 using the C-terminal specific antibody. RPGR-ORF15 protein (red) was present apical to the ONL-like layer in the CC region of RPGR-deficient photoreceptors in RK.RPGR-treated organoids, similar to healthy controls (Figures 7C, 7D, 6E, and 6F, respectively). In contrast, little RPGR-ORF15 protein (red) was detected in the CC region of RK.GFP-treated organoids (Figures 7A and 7B). Quantitative analysis of RPGR-ORF15 staining in the CC region demonstrated a significant increase in RPGR protein from 0.02 ± 0.04% positive pixels in RK.GFP to 0.41 ± 0.31% in RK.RPGR-treated organoids (Figure 7E; p < 0.05; Kruskal-Wallis with Dunn's MCT; n ≥ 12 images, N = 4 experiments). Although the RPGR signals did not reach the levels observed in healthy controls (1.33 ± 0.50% positive pixels), this is most likely due to incomplete transduction of all photoreceptors in the RPGR-deficient retinal organoids.

Having established RPGR-ORF15 supplementation, we examined GFAP upregulation and RHODOPSIN mis-localization in 35-week organoids. While immunohistochemical analysis of control-treated RPGR-deficient retinal organoids revealed GFAP upregulation (red), treatment with RK.RPGR resulted in decreased GFAP levels, similar to healthy controls (Figures 7F, 7G, S7F and S6M, respectively). Quantitative analysis of GFAP staining confirmed a significant decrease in GFAP+ Müller cells in RK.RPGR-treated compared with RK.GFP-treated organoids (Figure 7H; 0.13 ± 0.09% versus 0.99 ± 0.91% positive pixels, respectively; p = 0.005; two-tailed Mann-Whitney test; n ≥ 7 images, N = 3 experiments). In addition, immunohistochemical analysis of RK.GFP-treated organoids confirmed RHODOPSIN mis-localization to the cell body and processes of photoreceptors, as observed in untreated RPGR-deficient organoids (Figures 7I and 6P, respectively). In contrast, far less RHODOPSIN was observed mis-localized to the ONL-like layer in RK.RPGR-treated mutant photoreceptors (Figures 7J and S7G). To establish if the difference observed was significant, the intensity of RHODOPSIN staining was measured as the percentage of pixels above threshold in the ONL-like layer, not including the segment region. RHODOPSIN staining of the ONL-like layer was significantly reduced in RK.RPGR-treated, compared with RK.GFP-treated organoids (Figures 7K; 7.1 ± 5.6% versus 22.1 ± 21.3% positive pixels, respectively; p = 0.006; two-tailed Mann-Whitney test; n = 30 images, N = 6 experiments). This confirms the improved RHODOPSIN localization observed in RPGR-deficient retinal organoids, following gene supplementation. These findings demonstrate the benefit of enriched culture conditions to investigate therapeutic interventions using hPSC-derived retinal organoids and support the use of this shortened RPGR construct for rescuing function in human as well as mouse photoreceptors.

## Discussion

One limitation to the use of retinal organoids to model inherited retinal degenerations is that even after extensive long-term culture (>30weeks), hPSC-derived photoreceptors exhibit few nascent OS-like structures. Therefore, disease phenotypes related to the mature structure of photoreceptors, such as ciliopathies, have proved difficult to model effectively using retinal organoids alone. Here, we describe enhanced culture conditions that permit the generation of photoreceptors with well-developed OSs containing disc-like structures. These new conditions support the development of both rod and cone photoreceptor segments *in vitro*. Importantly, this protocol improves the proportion of photoreceptors that form OSs, providing a more robust model with which to investigate retinal disease. To demonstrate the utility of this modified protocol, we generated iPSC lines from patients with frameshift mutations in exon *ORF15* of the RPGR gene that result in XLRP3. RPGR-deficient retinal organoids demonstrated clear disease phenotypes including the mis-localization of RHODOPSIN, which was confined to the photoreceptor OSs in healthy control organoids but could also be found in the cell body and processes of mutant photoreceptors. This has been observed previously in KO mouse models but has not been reported using hPSC-derived photoreceptors to date, possibly because of the mis-localization of RHODOPSIN in control organoids, due to the inefficiency of OS formation (Megaw et al., 2017; Deng et al., 2018; Hong et al., 2005; Wu et al., 2015). Using AAV gene supplementation to restore RPGR to the mutant photoreceptors, we demonstrated a significant reduction in RHODOPSIN mis-localization, ameliorating the phenotype despite incomplete transduction efficiency. We have previously shown that gene therapy using a shortened RPGR-ORF15 transgene improves photoreceptor function and viability in an animal model of RPGR deficiency (Pawlyk et al., 2016). Here we demonstrate that the shortened transgene is also able to restore function in human photoreceptor cells, providing additional validation of a construct that is currently being used in clinical trials of gene therapy for XLRP3 (ClinicalTrials.gov: NCT03252847).

LC-PUFAs are known to be essential for both brain and retinal development, with dietary restriction resulting in reduced visual function (Wheeler et al., 1975; Neuringer et al., 1984). DHA, a major component of retinal phospholipids, is actively sequestered via the choroidal blood flow and influences rhodopsin content at the disk membranes, as well as photoresponses and disk morphogenesis (Shindou et al., 2017; Anderson et al., 1992; Nguyen et al., 2014). Previous studies have examined the effects of DHA on the differentiation and survival of photoreceptors, from primary retinal progenitors and, more recently, derived from both mouse and human PSCs (Arai et al., 2017; Rotstein et al., 1997; Brooks et al., 2019). Despite increased levels of rhodopsin expression and improved inner segment and CC formation, little improvement in brush border density or OS ultrastructure were demonstrated (Arai et al., 2017; Brooks et al., 2019). In this study, we determined that increased antioxidant levels were required to enable the beneficial effects of DHA to be observed. Therefore, the higher concentrations of BSA-bound lipids used here most likely account for the difference in findings (Arai et al., 2017; Brooks et al., 2019). The exact proportions of specific PUFAs present in lipid-rich BSA (AlbuMAX) are undefined. However, we demonstrate, for the first time to our knowledge, mESC-derived photoreceptors exhibiting membranous OS-like structures, following the addition of either BSA-bound DHA or lipid-rich BSA. Interestingly, the inclusion of BSA-bound lipids also improved the segment structures and M/L opsin content of hPSC-derived cone photoreceptors. Unlike rods, cone OSs are formed by a continuation of the plasma membrane and are comprised of lower levels of DHA or omega-3 relative to omega-6 PUFAs, suggesting a different biophysical lipid requirement (Young 1969; Agbaga et al., 2018). Metabolic cross-talk between the two photoreceptor subtypes, mediated by rod-derived cone viability factor, means we cannot exclude the possibility that improved cone OS formation is a secondary effect of enhanced rod photoreceptor maturation (Aït-Ali et al., 2015). It is also important to note that while our protocol resulted in the efficient generation of hPSC-derived photoreceptors exhibiting organized OSs, the formation and maintenance of perfectly stacked discs in all photoreceptors has yet to be achieved. This will most likely require additional support at the level of the photoreceptor segments, such as that provided by the close apposition of RPE cells *in vivo*. While little difference in gross OS morphology was observed for DHA supplemented retinal organoids, it remains to be determined if there is a functional difference between hPSC-derived photoreceptors grown with increased concentrations of DHA, as opposed to other LC-PUFAs. Despite this, we have continued to use our enhanced media in combination with DHA supplementation for retinal disease modeling. In addition, it will be of interest to establish if the improved OS ultrastructure observed here results in enhanced electrophysiological responses to light. Further studies are needed to address these limitations and determine if retinal organoids maintained with BSA-bound lipids can provide a light-responsive model system for the human retina.

In summary, we designed a nutrient-rich medium to support the increased energetic and biosynthetic demands of maturing PSC-derived photoreceptors. In contrast to our original culture conditions, in which only a limited number of hPSC-derived photoreceptors exhibited rudimentary OS-like structures, these new conditions permitted the efficient development of OS-bearing rod and cone photoreceptors. These improvements were not limited to the increased frequency of OS development in hPSC-derived photoreceptors, but also resulted in better organization, with membranous structures reminiscent of stacked OS discs. This enabled us to effectively model cellular defects in XLRP and demonstrate rescue by gene supplementation. Together, these findings suggest that our enhanced culture protocol facilitates the development of more structurally mature hPSC-derived photoreceptors, and this may be useful for inherited retinal disease modeling and *in vitro* testing of novel therapeutic strategies.

## Experimental procedures

See supplemental experimental procedures for detailed protocols.

### Mouse ESC culture and retinal differentiation

Mouse ESCs were maintained and differentiated to form EBs containing retinal regions as previously described (Kruczek et al., 2017). From day 21 onward the media used was either standard RMM, AOX, or ALT media (see Table S1), supplemented with 50 μM DHA, 12.5 μM fatty acid-free BSA, additional glucose (25 mM final concentration), or AlbuMAX II (0.4 mg/ml).

### Human PSC culture and retinal differentiation

hPSCs (see Table S2) were maintained and differentiated as previously described to generate NRVs (Gonzalez Cordero et al., 2017). From 12 weeks of differentiation, media were changed to either standard RDM90 or ALT medium and supplemented with 50 μM DHA or 12.5 μM fatty acid-free BSA.

### Production and use of recombinant AAV viral vector

Both pD10/RK*promoter-GFP* and pD10/RK*promoter-RPGR* constructs containing AAV-2 inverted terminal repeats were used to generate AAV7m8.RK.GFP and AAV7m8.RK.RPGR viral vector. Retinal organoids were infected at 15–18weeks with 3 × 10^11^ viral particles per organoid, with an estimated gMOI of 6 × 10^5^.

### Immunohistochemical analysis

See supplemental experimental procedures (Table S3) for full details. Images were acquired with a confocal microscopy (Leica DM5500Q) and LAS AF image software. Image analysis was performed using FiJi and Gimp 2.8.22 software and blinded, wherever possible.

### Ultrastructural analysis

For TEM, sections were imaged with a JEOL 11010 TEM operating at 80 V and acquired with a Gatan Orius camera using Digital Micrograph software. For scanning electron microscopy, specimens were imaged in a Zeiss Sigma FESEM operating at 3–5 kV. For 3view, stacks of backscatter electron micrographs were automatically acquired using a Gatan 3view system working in conjunction with a Zeiss Sigma field emission scanning electron microscope. Stacks were converted to TIFF images in Digital Micrograph software, prior to importation into Amira 5.3.3 software.

### Statistical analysis

In all experiments, means are presented ±SD, unless otherwise stated; n = number of images, sections, EBs, or organoids examined; N = number of independent differentiations, cell lines, or experiments performed. Graphpad Prism 6 software was used for statistical analysis.

## Author contributions

Conceptualization, E.L.W., A.G-C., J.W.B.B., and R.R.A.; Methodology, E.L.W., A.G-C., P.M., M.F., A.N., P.O-R., R.S., M.H., and A.G.; Investigation, E.L.W., A.G-C., P.M., A.N., M.F., M.O’H-W., E.L., R.S., J.R., N.J., and I.O.S.; Resources, M.H. and J.W.B.B.; Writing – Original Draft, E.L.W. and A.G-C.; Writing – Review & Editing, A.G., A.J.S., and R.R.A.; Supervision, A.J.S., J.W.B.B., and R.R.A.; Funding Acquisition, J.W.B.B. and R.R.A.

## Conflicts of interest

The authors declare no competing interests.

## Supplemental information

Document S1. Supplemental experimental procedures, Figures S1–S7, and Tables S1–S4

Document S2. Article plus supplemental information
