# Supplementary material for: Antioxidant and lipid supplementation improve the development of photoreceptor outer segments in pluripotent stem cell-derived retinal organoids
Source: Stem Cell Reports. 2022 Mar 24;17(4):775–88. doi: 10.1016/j.stemcr.2022.02.019 (PMC9023802; doi:10.1016/j.stemcr.2022.02.019)
Supplement: Document S1. Supplemental experimental procedures, Figures S1–S7, and Tables S1–S4 [file mmc1.pdf]

Stem Cell Reports, Volume *17*

Supplemental Information

Antioxidant and lipid supplementation improve the development of photoreceptor outer segments in pluripotent stem cell-derived retinal organoids

Emma L. West, Paromita Majumder, Arifa Naeem, Milan Fernando, Michelle O'Hara-Wright, Emily Lanning, Magdalena Kloc, Joana Ribeiro, Patrick Ovando-Roche, Ian O. Shum, Neeraj Jumbu, Robert Sampson, Matt Hayes, James W.B. Bainbridge, Anastasios Georgiadis, Alexander J. Smith, Anai Gonzalez-Cordero, and Robin R. Ali

Supplemental Information

Supplemental Figures

Figure S1. Long term survival and organisation of mESC-derived photoreceptors in retinal cultures, *related to Figure 1*

(**A**-**H**) Immunohistochemical images demonstrating examples of embryoid body sections for the relevant categories of organisation, non-retinal cells (**A**,**E**), organised PRs (**B**,**C**,**F**,**G**) and disorganised PRs (**D**,**H**). DAPI (blue) was used to determine the presence of ONL-like layers (dashed white lines) within retinal regions, that contained organised Recoverin+ photoreceptors (red; white arrow heads). Crx.GFP^{+} photoreceptors (green) corresponded to the Recoverin^{+} regions observed. (**I**) Flow cytometry plots showing the gating strategy used to determine the percentage of Crx.GFP^{+}/CD73^{+} rod photoreceptors present in dissociated retinal cultures with time. Control samples included an unstained CCE cell sample (**Unstained cells**), unstained Crx.GFP cell sample (**Crx.GFP cells**), CD73 stained CCE cell sample (**CD73 stained cells**) and CD73 stained Crx.GFP cell sample (**Stained Crx.GFP cells**).
Scale bars: 200 μm (**A-D**) and 50 μm (**E-H**). Abbreviations: PRs, photoreceptors.

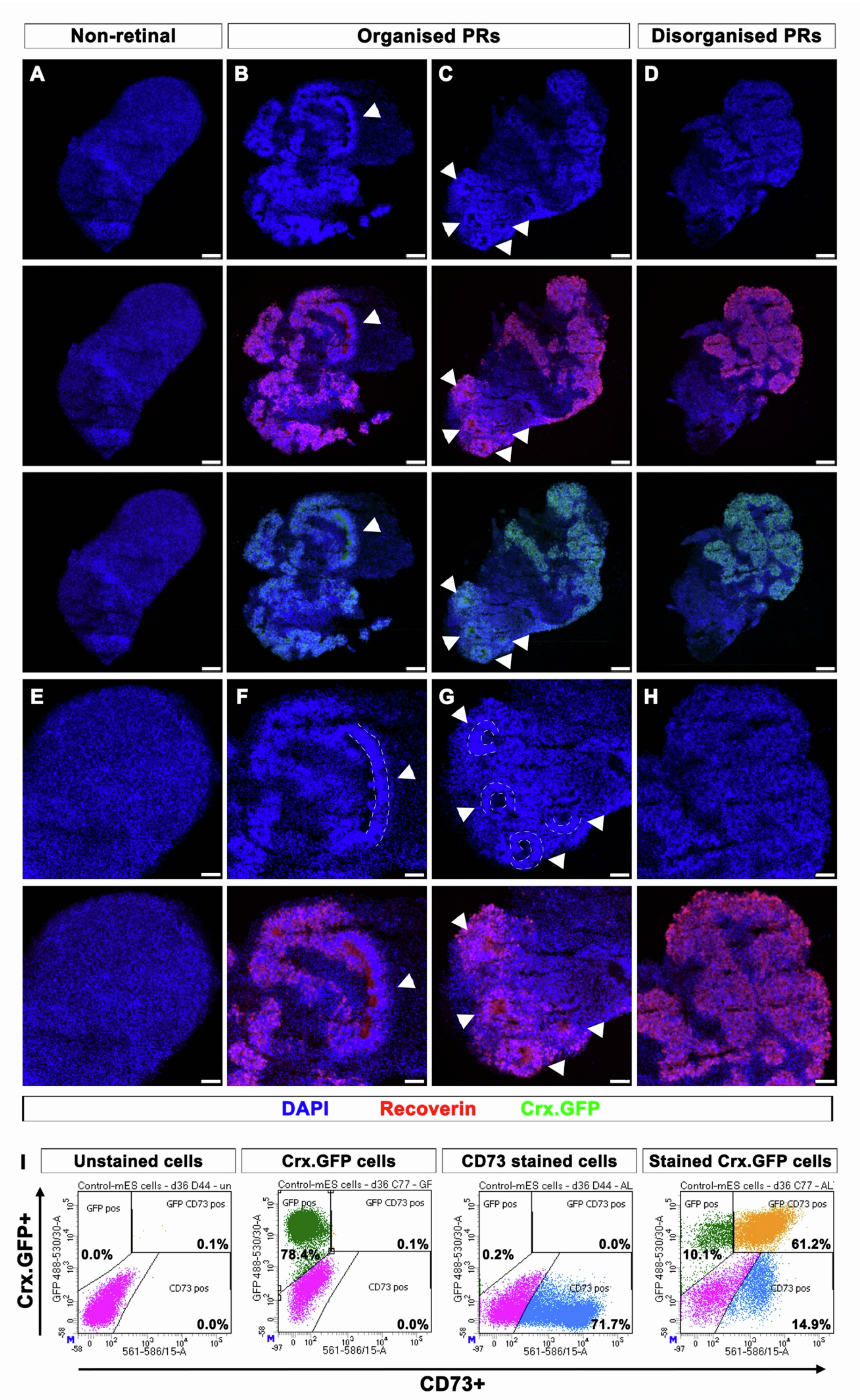

Non-retinal
Organised PRs
Disorganised PRs
A
B
C
D
E
F
G
H
DAPI
Recoverin
Crx.GFP
I
Unstained cells
Crx.GFP cells
CD73 stained cells
Stained Crx.GFP cells
GFP pos
GFP CD73 pos
CD73 pos
0.0%
0.1%
0.0%
78.4%
0.1%
0.0%
0.2%
0.0%
71.7%
10.1%
61.2%
14.9%
GFP 488-530/30-A
561-586/15-A
Crx.GFP+
CD73+

Figure S2. Characterisation of photoreceptor morphology and organisation in various culture conditions using different mESC lines, *related to Figure 1*

(**A**-**F**) Representative confocal images of mESC-derived retinal neuroepithelia (Crx.GFP line, green photoreceptors) at day 34, maintained in 3 different culture media with +BSA or +DHA and stained for Rhodopsin (grey) and Peripherin2 (red). (**G**-**L**) Representative confocal images of mESC-derived retinal neuroepithelia (CCE cell line) at day 34, maintained in 3 different culture media with +BSA or +DHA and stained for Rhodopsin (grey) and Peripherin2 (red). (**M**) Histogram showing the percentage of sections as classified into 3 set categories (non-retinal, organised and disorganised PRs) maintained in AOX culture media with +BSA, additional glucose (+Glucose), lipid-rich BSA (+AlbuMAX) and +DHA (error bars, mean ± SEM, $p > 0.05$; n>12 sections, N>3 independent experiments). (**N**-**Q**) Representative confocal images of mESC-derived retinal regions (Crx.GFP line; green photoreceptors) at day 34, maintained in AOX culture media with with +BSA (**N**), additional glucose (**O**; +Glucose), lipid-rich BSA (**P**; +AlbuMAX) and +DHA (**Q**) and stained for Rhodopsin (grey) and Peripherin2 (red).
Scale bars: 10 μm (**A**-**F**, **N-Q**) and 25 μm (**G**-**L**). Abbreviations: PRs, photoreceptors.

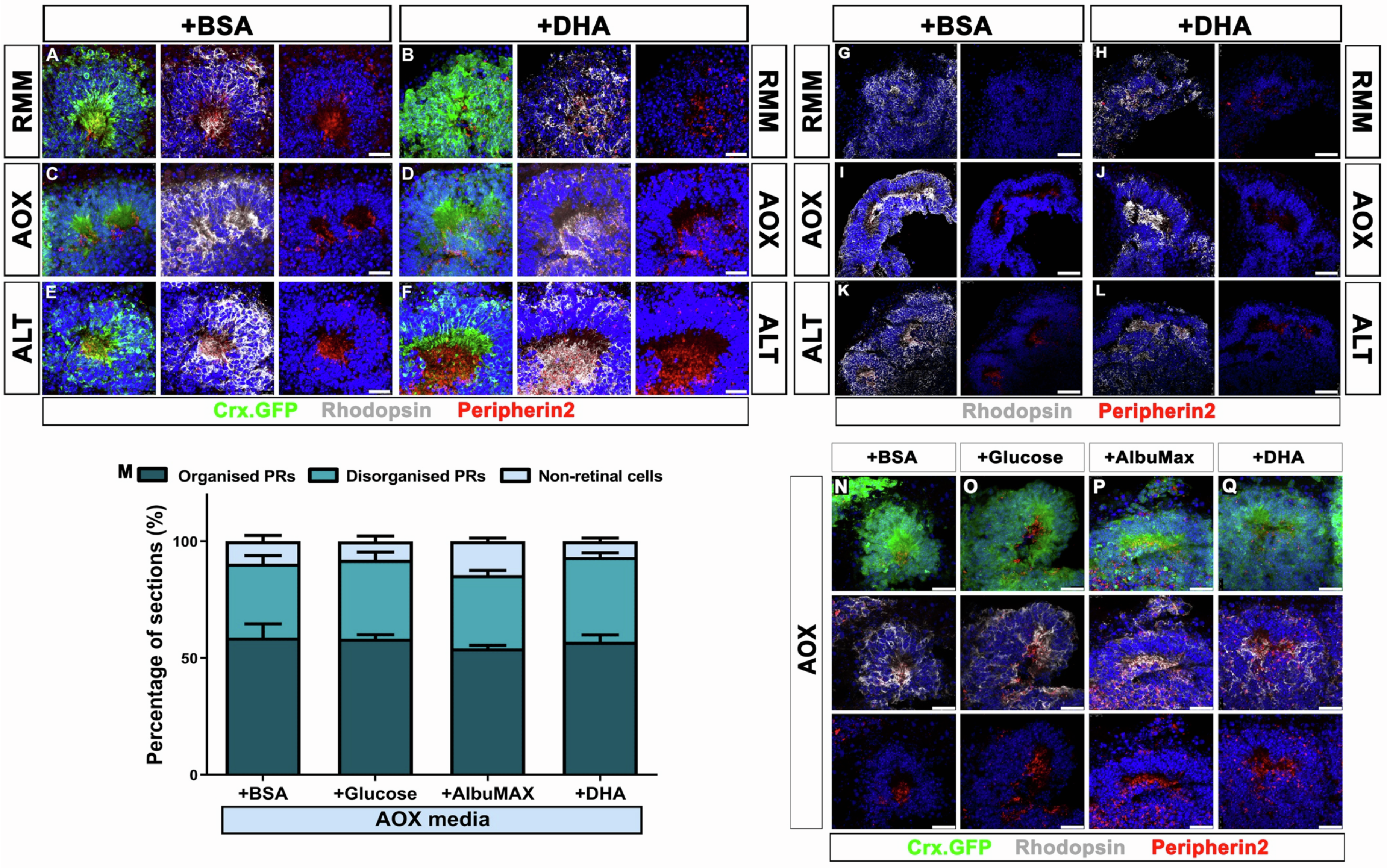

+BSA
+DHA
A
B
C
D
E
F
RMM
AOX
ALT
Crx.GFP
Rhodopsin
Peripherin2
+BSA
+DHA
G
H
I
J
K
L
RMM
AOX
ALT
Rhodopsin
Peripherin2
M
Organised PRs
Disorganised PRs
Non-retinal cells
Percentage of sections (%)
100
50
0
+BSA
+Glucose
+AlbuMAX
+DHA
AOX media
+BSA
+Glucose
+AlbuMax
+DHA
N
O
P
Q
AOX
Crx.GFP
Rhodopsin
Peripherin2

Figure S3. Characterisation of segment morphology in DHA and ALT supplemented retinal organoid cultures, *related to Figure 2 & 3*

(**A**) Representative bright field image of wk 26 retinal organoid. (**B**) High magnification image of neuroepithelia showing brush border regions in standard RDM90+BSA media. (**C**) IHC analysis showing MITOCHONDRIA rich ISs and developing PRPH2+ OSs. (**D, E**) Representative bright field image of wk 26 retinal organoid and neuroepithelia (**E**, high magnification) showing no differences in brush border developed in RDM90+DHA media. (**F**) IHC analysis showing MITOCHONDRIA rich ISs and developing PRPH2+ OSs. (**G**) Image of RDM90+BSA cultured retinal organoid showing ESPIN positive structures in the CC region and ABCA4 positive OSs. (**H**) Image of ALT+BSA cultured retinal organoid showing ESPIN positive structures in the CC region and elongated ABCA4 positive OSs.
(**I-P**) Representative bright field images of retinal organoids generated from various pluripotent stem cell lines, cultured in either RDM90+BSA (**I**, **K**, **M**, **O**) or ALT+BSA (**J**, **L**, **N**, **P**).
Scale bars: 25μm (**C, F**, **G**, **H**), 50μm (**A-E**, **I-P**). Abbreviations: CC, connecting cilia; IS, inner segment; OS, outer segment; ONL, outer nuclear layer.

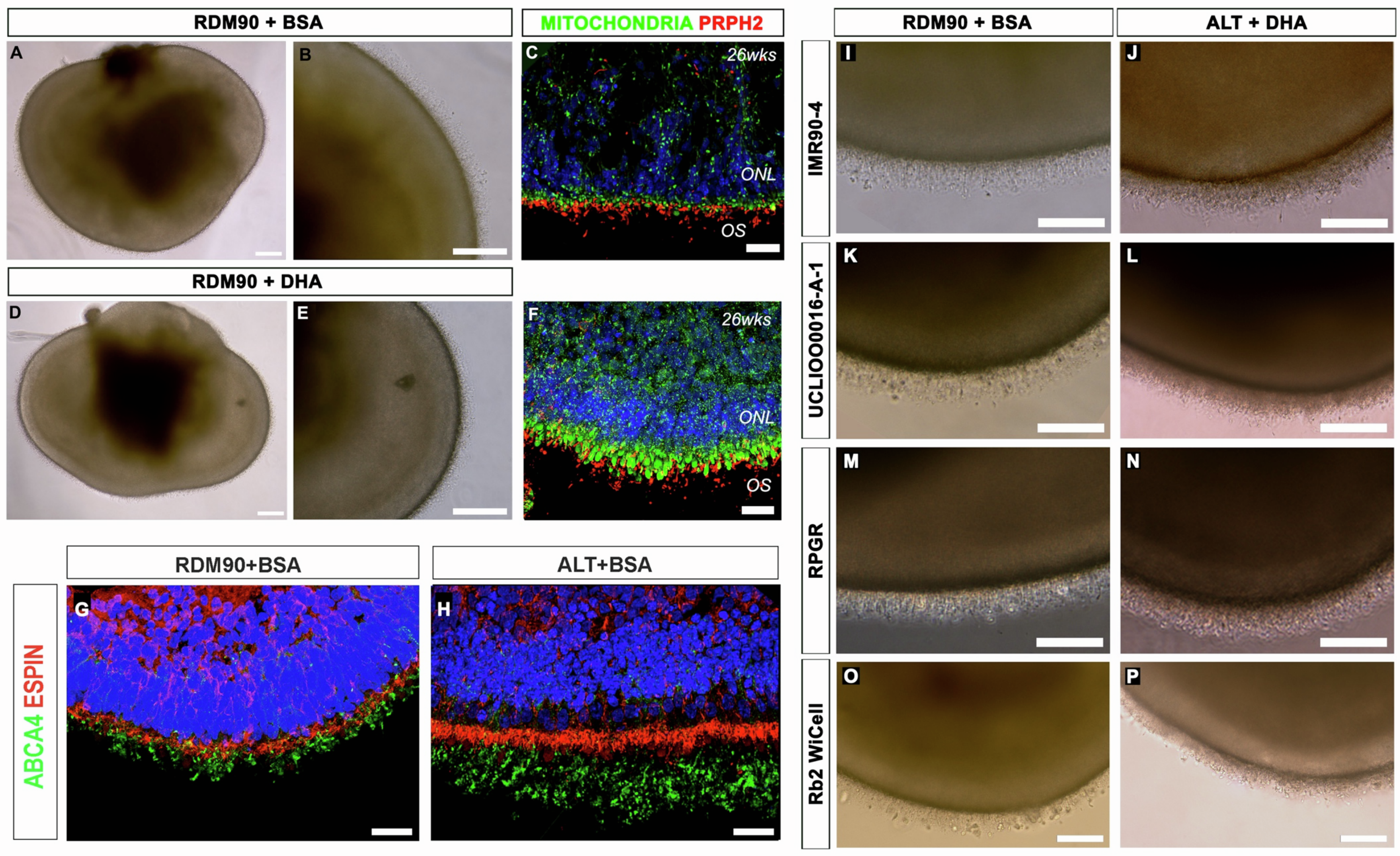

RDM90 + BSA
A
B
MITOCHONDRIA PRPH2
C
26wks
ONL
OS
RDM90 + DHA
D
E
F
26wks
ONL
OS
ABCA4 ESPIN
RDM90+BSA
G
ALT+BSA
H
RDM90 + BSA
ALT + DHA
IMR90-4
I
J
UCLIOO0016-A-1
K
L
RPGR
M
N
Rb2 WiCell
O
P

Figure S4. Ultrastructure analysis of the segment region of retinal organoids in various conditions, *related to Figure 5*

(**A**-**C**) Electron micrographs showing RDM90+BSA grown photoreceptor cells. Insets in **B** highlight two OSs that are shown in higher magnification in panels above.
(**D-F**) Images showing OSs of ALT+BSA cultures. Inset is shown in higher magnification in the panel above**.** OS disk membranes were more discernable in these cultures (**D**-**F**). (**G-K**) Electron micrographs of an ALT+DHA grown retinal organoid showing the brush border region. The ultrastructure of this region of the retinal neuroepithelia demonstrated numerous OSs (**G**). Dashed line inset in **G** is shown in high magnification in **H** and **I** to highlight OS morphology, with clearly visible stacked OS disk membranes. Solid line inset in **G** is shown in higher magnification in **K** and **J** to highlight another region with a number of OSs, with clearly visible stacked OS disk membranes.
Scale bars: 1μm (**E**), 2μm (**B**), 5μm (**A**, **D**, **I**, **J**), 10μm (**C**, **G**, **H**, **K**). Abbreviations: OS, outer segment; CC, connecting cilia; IS, inner segment.

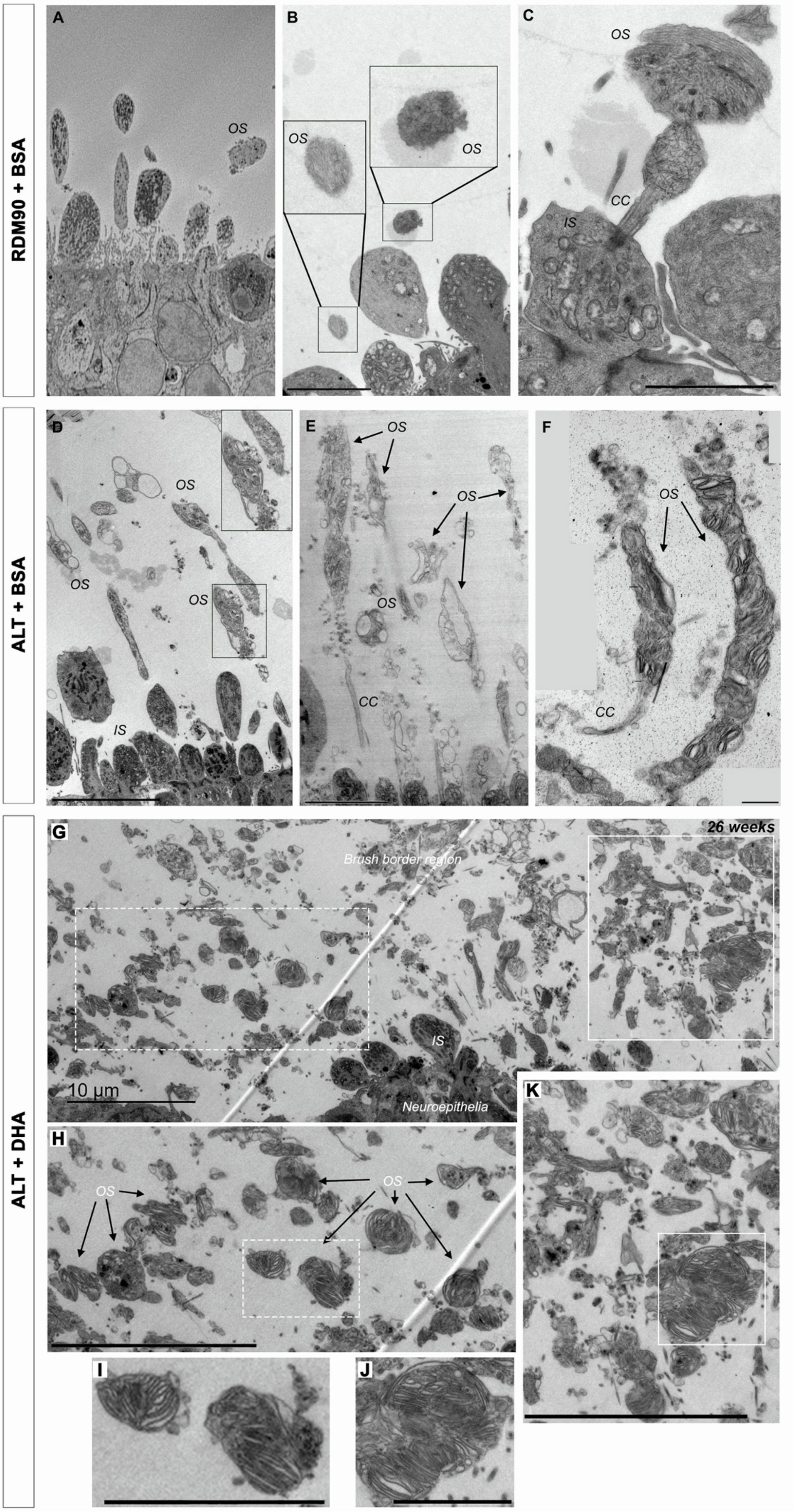
RDM90 + BSA
A
OS
B
OS
OS
C
OS
CC
IS
ALT + BSA
D
OS
OS
OS
IS
E
OS
OS
OS
CC
F
OS
CC
ALT + DHA
G
26 weeks
Brush border region
IS
10 µm
Neuroepithelia
H
OS
OS
K
I
J

Figure S5. 3D Ultrastructural analysis of photoreceptor segment structures present in RDM90 and ALT cultured retinal organoids, *related to Figure 5*

(**A-D**) 3view sequence of backscatter EM images of hPSC-derived retinal neuroepithelia showing photoreceptor OS (magenta), CC (blue) and IS (green). (**A**, **C**) 3view 3D reconstruction of 150 sections with thickness of 100 nm each. (**B**, **D**) Single 3view section of 100nm. (**E**, **J**) Representative bright field images of retinal organoids cultured in RDM90+BSA or ALT+BSA, showing brush border structures apical to neuroepithelia regions. (**F**, **K**) SEM micrographs showing topography of whole retinal organoids highlighting the neuroepithelia region. (**G-I**, **L-N**) Topographic features of neuroepithelia showing photoreceptor cell density and morphology from RDM90+BSA (**G-I**) or ALT+BSA (**L-N**) cultured organoids, at ascending magnifications.

Scale bars: 2μm (**M, N**), 3μm (**H**, **I**), 10μm (**G**, **L**), 20μm (**F**, **K**), 50μm (**E**, **J**). Abbreviations: CC, connecting cilium; IS, inner segment; OLM, outer limiting membrane; ONL, outer nuclear layer; OS, outer segment; RPE, retinal pigment epithelium.

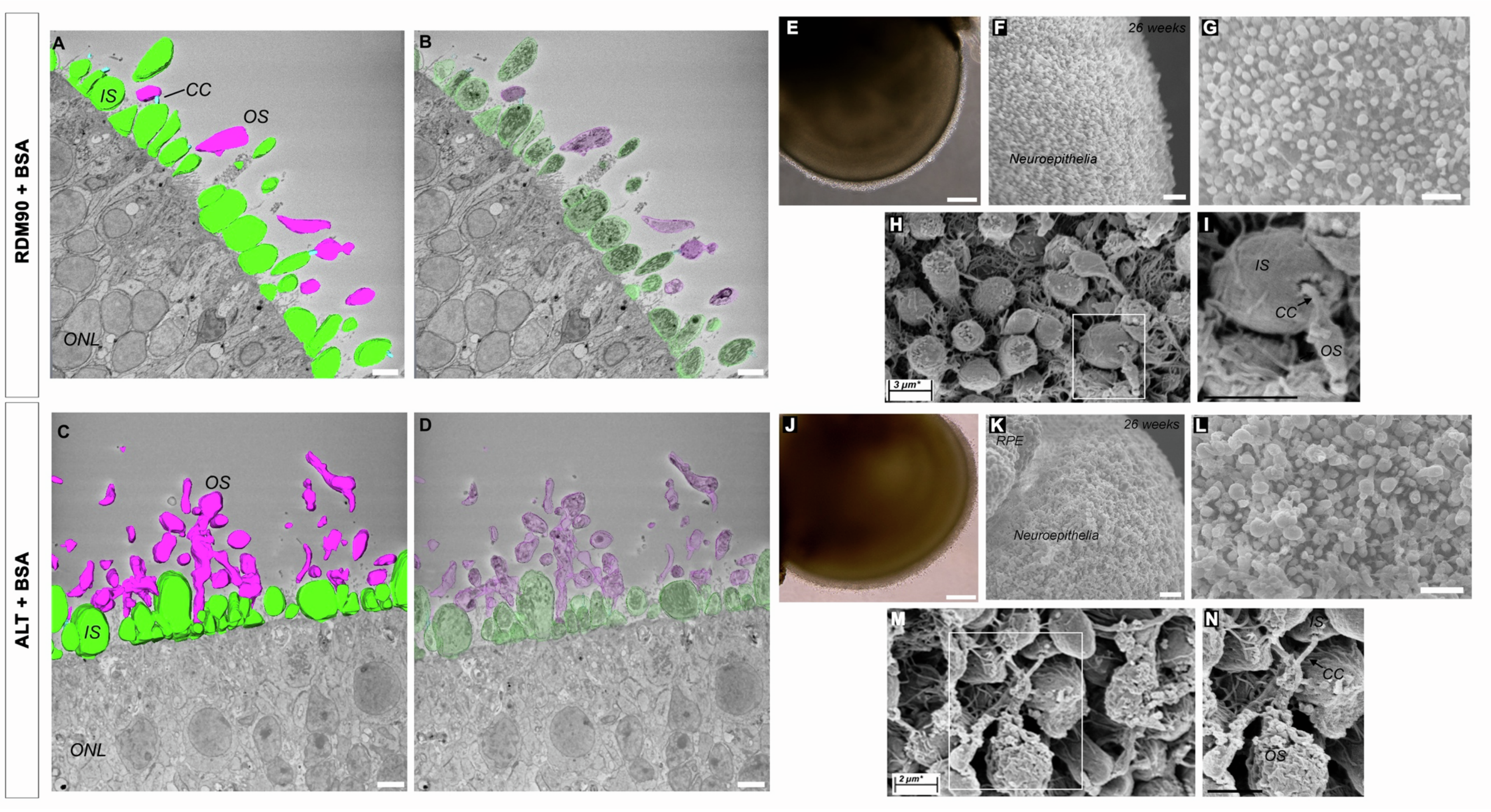

RDM90 + BSA
ALT + BSA
A
IS
CC
OS
ONL
B
C
OS
IS
ONL
D
E
F
26 weeks
Neuroepithelia
G
H
3 µm*
I
IS
CC
OS
J
K
RPE
26 weeks
Neuroepithelia
L
M
2 µm*
N
IS
CC
OS

Figure S6. RPGR iPSC line characterisation, *related to Figure 6*

(**A-R**) Typical iPSC colonies appearance and morphology for all 3 RPGR patient iPSC lines generated (**A, G, M**). All iPSC lines showed a normal male karyotype (**B, H, N**). Immunocytochemistry of iPSCs demonstrated the presence of pluripotency markers SOX2, OCT3/4, C-MYC and NANOG for all lines (**C-F, I-L, O-R**). (**S-U**) Sequencing confirmed the presence of mutations within the RPGR *ORF15* gene for all lines. (**V**) The TaqMan hPSC Scorecard assay was performed on all hPSC lines used in this study. Box and whisker plot confirmed that RPGR1-3 iPSC lines average expression of self-renewal and embryonic germ layer genes fall within the expected range in terms of self-renewal, ectoderm and mesoderm gene expression and are similar to the 3 control lines (H9, Rb2 and IMR90-4) used in this study. (**W**) Representative bright field images of iPSC-derived retinal organoids from all 3 RPGR patient lines, at 7-9 weeks of development.
Scale bars: 75µm (**C-F, I-L, O-R**), 100µm (**W**).

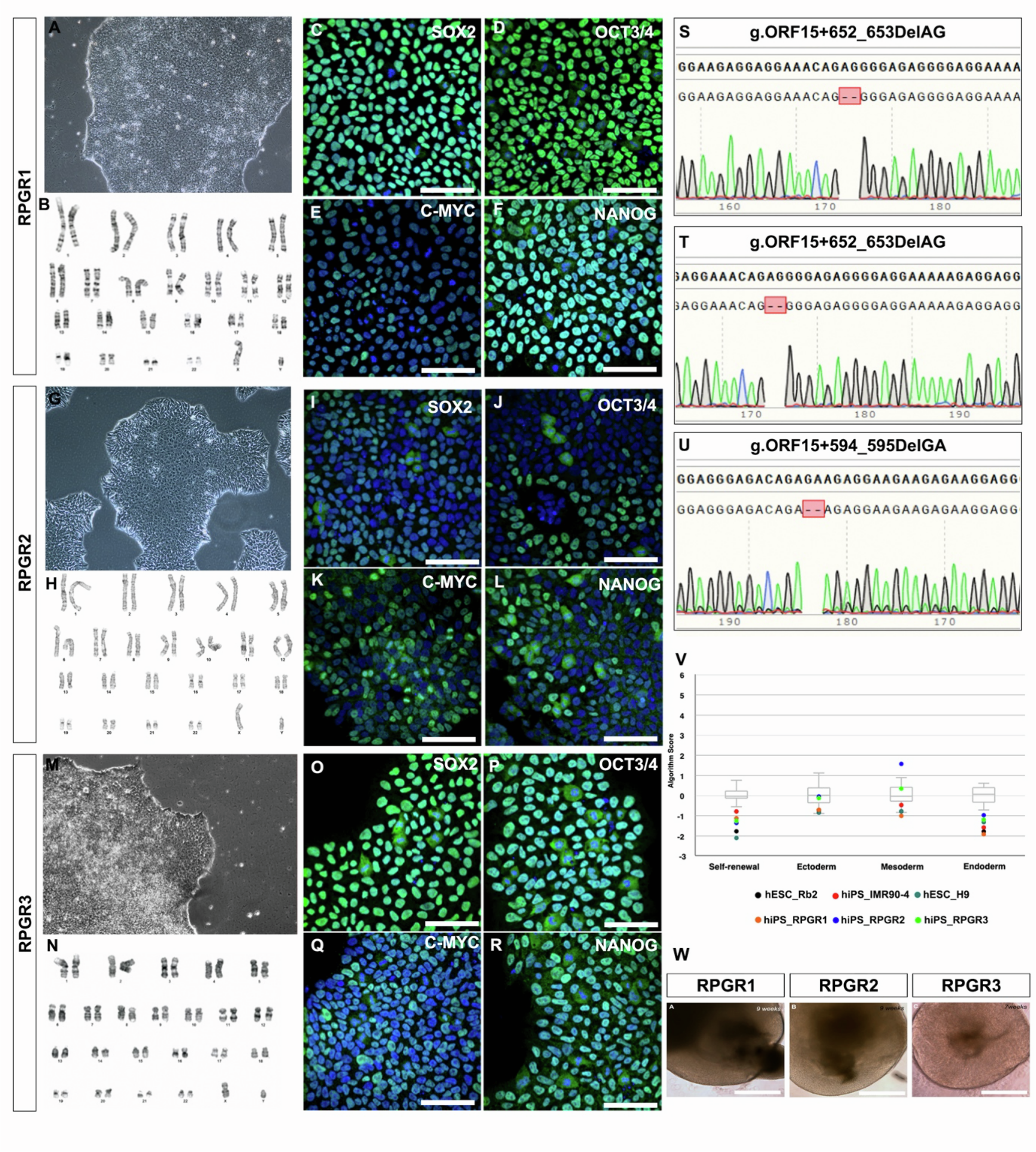
RPGR1
RPGR2
RPGR3
A
B
C SOX2
D OCT3/4
E C-MYC
F NANOG
G
H
I SOX2
J OCT3/4
K C-MYC
L NANOG
M
N
O SOX2
P OCT3/4
Q C-MYC
R NANOG
S g.ORF15+652_653DelAG
GGAAGAGGAGGAAACAGAGGGGAGAGGGGAGGAAAA
GGAAGAGGAGGAAACAG--GGGAGAGGGGAGGAAAA
160 170 180
T g.ORF15+652_653DelAG
AGGAAACAGAGGGGAGAGGGGAGGAAAAAGAGGAGG
AGGAAACAG--GGGAGAGGGGAGGAAAAAGAGGAGG
170 180 190
U g.ORF15+594_595DelGA
GGAGGGAGACAGAGAAGAGGAAGAAGAGAAGGAGG
GGAGGGAGACAGA--AGAGGAAGAAGAGAAGGAGG
190 180 170
V
Algorithm Score
6 5 4 3 2 1 0 -1 -2 -3
Self-renewal
Ectoderm
Mesoderm
Endoderm
hESC_Rb2
hiPS_IMR90-4
hESC_H9
hiPS_RPGR1
hiPS_RPGR2
hiPS_RPGR3
W
RPGR1
RPGR2
RPGR3

Figure S7. Characterisation of iPSC-derived RPGR-deficient retinal organoids, with and without AAV-mediated gene supplementation, *related to Figure 6 & 7*

(**A**) IHC analysis showing no differences in Phalloidin (magenta) expression in the OLM of control (IMR90-4) and RPGR-deficient organoids. (**B**) IHC showing upregulation of GFAP (green) in RPGR-deficient compared to control retinal neuroepithelia. (**C**) IHC showing co-localisation of CRALBP (grey) and GFAP (green) in Müller glial cells. (**D**) IHC analysis for RHODOPSIN (red) and Phalloidin (grey) in control and RPGR-deficient retinal organoids showing mis-localisation of RHODOPSIN to the ONL region. Insets are shown at a higher magnification in the top right panel, without DAPI (blue), for these regions. (**E**) Schematic showing the experimental design for the treatment of iPSC-derived retinal organoids with viral vectors. Representative image of an RPGR-deficient retinal organoid transduced with AAV 7m8.RK.GFP, at 33 wks in culture. The transduction efficiency of AAV 7m8.RK.GFP, shown by the percentage of GFP+ cells in the ONL-like layer of retinal organoid sections, was 44 ± 11.4% (mean ± SD; n = 13 sections, N = 5 ROs). (**F**) IHC images showing few GFAP+ activated Müller glial cells in iPSC-derived RPGR-deficient retinal organoids treated with RK.RPGR viral vector. (**G**) IHC image showing the presence of RHODOPSIN in the OSs but not the cell bodies of RPGR-deficient photoreceptors, following RPGR gene supplementation.
Scale bars: 25μm (**A**, **D**, **E-G** and insets). Abbreviations: OLM, outer limiting membrane; ONL, outer nuclear layer; OS, outer segment; RO, retinal organoid.

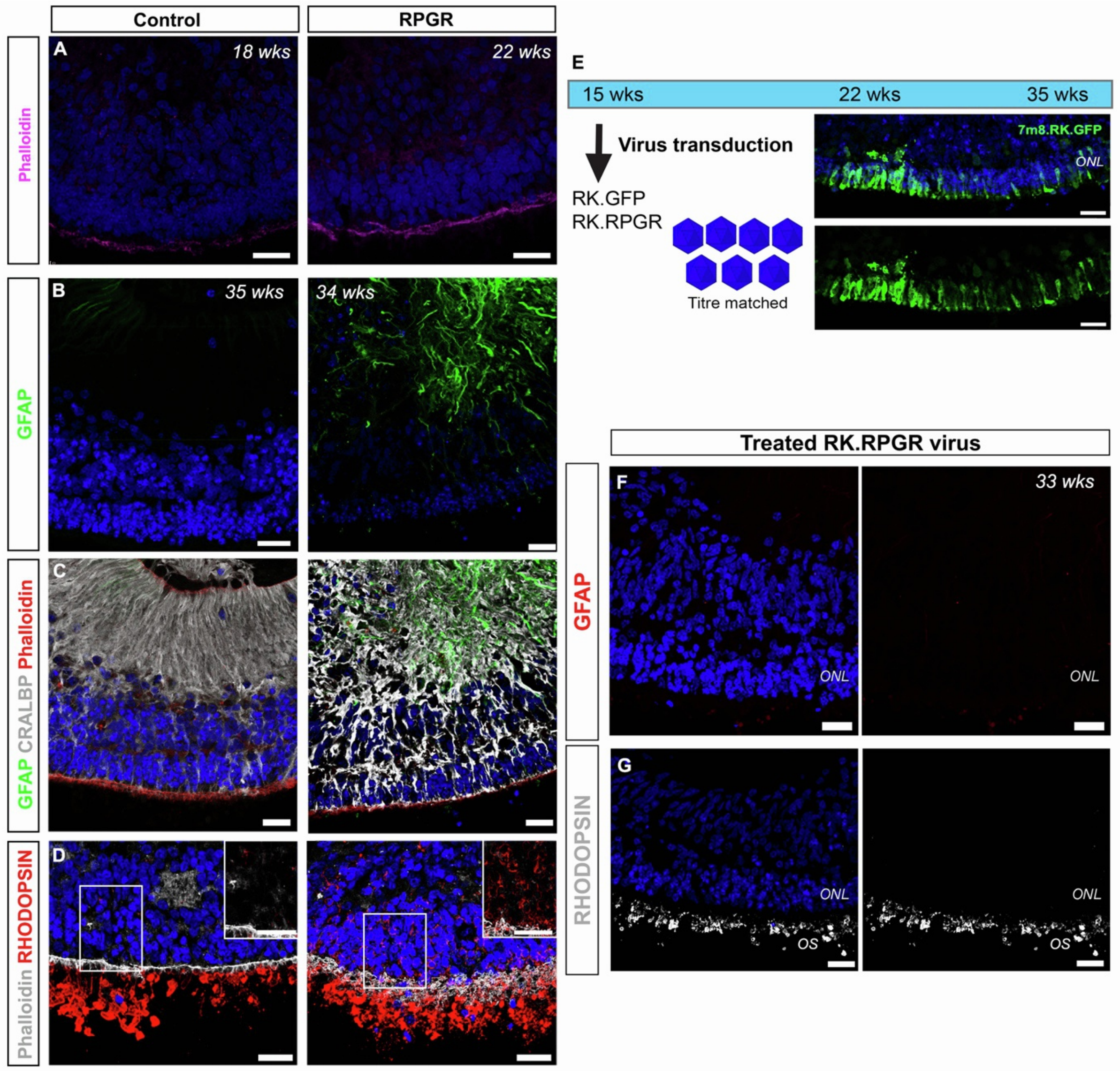

Control
RPGR
A
18 wks
22 wks
Phalloidin
B
35 wks
34 wks
GFAP
C
GFAP CRALBP Phalloidin
D
Phalloidin RHODOPSIN
E
15 wks
22 wks
35 wks
Virus transduction
RK.GFP
RK.RPGR
Titre matched
7m8.RK.GFP
ONL
Treated RK.RPGR virus
F
33 wks
GFAP
ONL
ONL
G
RHODOPSIN
ONL
OS
ONL
OS

Supplemental Video File 1. 3view EM movie through the segment region of an ALT grown retinal organoid, *related to Figure 5.*
3view 3D reconstruction of 150 sections with thickness of 100 nm each. 3view sequence of backscatter EM images of hPSC-derived retinal neuroepithelia showing photoreceptor OS (magenta), CC (blue) and IS (green).

Supplemental Tables

Supplemental Table 1. Media composition

Media Components/Concentrations	RMM	AOX	ALT
DMEM/F12 (Glutamax) Media	✓	✓	___
Advanced DMEM/F12 Media†	___	___	✓
N-2 Supplement	✓	✓	✓
B-27 (-Vit A) Supplement‡	___	✓	✓
Glucose concentration	17.5mM	17.5mM	25mM
Glutamax Supplement concentration	2.5mM	2.5mM	4mM
Antibiotic/Antimycotic	✓	✓	✓
Taurine	0.25mM	0.25mM	0.25mM
Retinoic Acid (*added at day 14 - 21 only)	0.5μM*	0.5μM*	0.5μM*

†contains 400mg/L AlbuMAX® II
‡contains vitamin E, vitamin E acetate, superoxide dismutase, catalase and glutathione antioxidants

Supplemental Table 2. Details of cell lines used

Cell Line	Type	Source
CCE line (EK.CCE 129/SvEv)	msESC	Kind gift of E. Robertson
Crx.GFP line (B6.SJL-Tg(Crx-GFP,-ALPP)1Clc/J)	msESC	Kind gift of Y. Arsenijevic
Nrl.GFP line (B6.Cg-Tg(Nrl-EGFP)1Asw/J)	msESC	Generated in-house
H9, WA09 (WAe009-A)	hESC	WiCell Stem Cell Bank
RB2 (WIC-WA09-RB-002)	hESC	WiCell Stem Cell Bank
IMR90-4 (WISCi004-B)	hiPSC	WiCell Stem Cell Bank
RPGR1 (UCLIOO004-A)	hiPSC	Generated in-house
RPGR2 (UCLIOO0010-A-4)	hiPSC	Generated in-house
RPGR3 (UCLIOO0011-A-1)	hiPSC	Generated in-house
UCLIOO0016-A-1	hiPSC	Generated in-house

Abbreviations: msESC, mouse embryonic stem cell; hESC, human embryonic stem cell; hiPSC, human induced pluripotent stem cell.

Supplemental Table 3. Antibodies used for immunohistochemistry			
Antigen	Host species	Concentration used	Supplier
**Nrl**	goat	1 in 200	R & D Systems (AF2945)
**M/L Opsin**	rabbit	1 in 100	Millipore (AB5405)
**CRALBP**	mouse	1 in 500	Abcam (AB15051)
**Crx**	mouse	1 in 800	Abnova (H00001406-M02)
**Mitochondria**	mouse	1 in 200	Millipore (MAB1273)
**GFAP**	rat	1 in 300	Calbiochem (345860)
**Arrestin3**	goat	1 in 100	Novus (NBP1-37003)
**Sox2**	mouse	1 in 200	Stemlight Cell signaling technology (NEB 9092S)
**Nanog**	rabbit	1 in 200	Stemlight Cell signaling technology (NEB 9092S)
**c-Myc**	mouse	1 in 200	Stemlight Cell signaling technology (NEB 9092S)
**Oct3/4**	rabbit	1 in 200	Stemlight Cell signaling technology (NEB 9092S)
**Recoverin**	rabbit	1 in 1000	Chemicon (AB5585)
**Rhodopsin**	mouse	1 in 1000	Sigma (O4886)
**Acetylated α-tubulin**	mouse	1 in 200	Abcam (ab24610)
**ARL3**	mouse	1:100	New East Bioscience (26070)
**ARL13b**	rabbit	1:1000	Proteintech (17711)
**Rxrγ**	rabbit	1 in 200	Abcam (ab15518)
**RETGC**	rabbit	1 in 100	Gift from K. Palczewski
**N-terminal RPGR**	rabbit	1 in 1000	Novus (NBP-57905)
**C-terminal RPGR ORF15**	rabbit	1 in 100	Gift from Alan Wright
**Peripherin-2**	rabbit	1 in 1000	Gift from Gabriel Travis
**Pericentrin (PCN)**	rabbit	1 in 1000	Abcam (ab4448)
**ABCA4**	mouse	1 in 100	Abcam (ab77285)

Supplemental Table 4. Gene-specific Primers used				
Gene name	Forward Primer (5'-3')	Reverse Primer (5'-3')	Amplicon size (bp)	Probe number
CRX	caccaggctgtgccctac	tgggtcttggcaaacagtg	107	17
ABCA4	gcgtctctggctgaagatg	tcaactctcaagtccgtccag	116	1
Rhodopsin	gcctcatcgtcacccagt	tcatctatatcatgatgaacaagcag	88	84

Supplemental Experimental Procedures

Mouse ESC culture and retinal differentiation

The mouse embryonic stem cell (mESC) lines (**Table S2**) were maintained on feeder free conditions, as previously described (Osakada et al., 2009). For 3D retinal differentiation, 3 × 10^3 dissociated mESCs were resuspended in differentiation medium (GMEM containing 1.5% KSR, 0.1 mM NEAA, 1 mM pyruvate, 0.1 mM 2-mercaptoethanol) and plated into each well of a 96-well ultra low-binding U-bottom plate (Nunclon Sphera, Thermo) and incubated at 37 °C, 5% CO_2. This was defined as day 0 of differentiation culture. Growth factor-reduced Matrigel (BD Biosciences) was added to embryoid body (EB) cell aggregates on day 1 of culture to a final concentration of 2% (v/v). For whole EB (wEB) retinal differentiation, wEBs were transferred into retinal maturation medium (RMM; DMEM/F12 Glutamax containing N2 supplement and Pen/strep) at day 9, plated in 24-well low-binding plates (Corning) at a density of 6 wEBs/well and incubated at 37 °C, 5% CO_2. The media was changed every 2–3 days, with the addition of 1 mM taurine (Sigma) from day 14 onwards and 500 nM retinoic acid (RA; Sigma) from day 14 - 21 of culture only. For long term culture and media testing wEBs were maintained in either RMM, antioxidant-rich media (AOX; DMEM/F12 Glutamax containing N2 supplement, B27 supplement without RA and Pen/strep) or advanced long-term media (ALT; Advanced DMEM/F12, B27 supplement without RA, N2 supplement, 4mM glutamax, 7.5 mM glucose and AA) from day 21 of differentiation culture onwards. In addition, docosahexaenoic acid (DHA) was prepared at a 4:1 carrier ratio with fatty acid-free BSA and added at a final concentration of 50μM, with fatty acid-free BSA used as the vehicle control. Where additional components were added to AOX media the final concentration was 25 mM glucose (+Glucose) or 0.4mg/ml AlbuMAX® II (+AlbuMAX).

Human PSC culture and retinal differentiation

The human embryonic and induced pluripotent stem cell lines (**Table S2**) were maintained in feeder free conditions with E8 (Thermo Fisher) and on geltrex coated 6 well plates. Briefly, when 80% confluent hPSCs were dissociated using Versene solution for 10 minutes. PSC small clumps were collected, washed twice with PBS and resuspended in E8 media for further maintenance culture on 6 well plates. For retinal neuroepithelial differentiation human PSCs were maintained as described above until 90-95% confluent, then media without FGF (E6, Thermo Fisher) was added to the cultures for two days (D1 and 2 of differentiation) followed by a neural induction period (up to 7 weeks) in proneural induction media (PIM; Advanced DMEM/F12, MEM non-essential amino acids, N2 Supplement, 100mM Glutamine and Pen/Strep). Lightly- pigmented islands of retinal pigmented epithelium (RPE) appeared as early as week 3 in culture. Optic vesicles were formed from within the RPE region between weeks 4 and 7. During this period neuroretinal vesicles were manually excised with 21G needles and kept individually in low binding 96 well plates in retinal differentiation media (RDM; DMEM, F12, Pen/Strep and B27 without retinoic acid). At 6 wks of differentiation retinal differentiation medium was

supplemented with 10% FBS, 100uM taurine (Sigma, T4871) and 2mM glutamax and at 10 wks 1 uM retinoic acid (RA) was added (RDM+ Factors media). At 10 wks of culture vesicles were transferred to low binding 24 well plates (5 vesicles/well). At 12 wks of differentiation, media was changed again to either our standard RDM90 media, which is RDM+ Factors supplemented with N2 supplement and 0.5 uM RA or the advanced long term (ALT) media, composed of Advanced DMEM/F12, B27 without RA, N2 supplement, 4mM glutamax, 7.5 mM glucose, 100uM taurine, 0.5 uM RA and Pen/Strep. Maintenance cultures of hPSCs were feed daily and differentiation cultures were feed every 2-3 days. All representative images in the paper were from Wicell H9 ESC line, unless stated otherwise. To further validate the system, we assessed segment formation in a second ESC line (H9Rb2, Wicell), an iPSC control line (IMR90-4, Wicell) and several hiPSC lines from patients (**Table S2**). In all cell lines tested (N>5 cell lines), an enhanced brush border was observed (see **Figures 2**, **3**, **6A** and **Figure S7**).

Generation of human iPSC lines

Following informed consent, peripheral blood was taken from individuals with confirmed XLRP3 inherited retinal degeneration. The study followed the tenets of the Declaration of Helsinki and was approved by the Moorfields and Whittington Hospitals' local Research Ethics Committees and the NRES Committee London Riverside Ethics Committee (REC 11/H0721/13).
On day 0, 2x 10^6 PBMCs were defrosted and added to 10ml of prewarmed (37°C) Stemspan 3000 with Penicillin/ Streptomycin (SS Medium). Following centrifugation at 300g for five minutes, the pellet of cells was resuspended in Expansion Medium (EM) and plated in one well of a 12-well plate. On day 3, cells were collected, centrifuged, resuspended in 2ml of EM Medium and plated in one well of a 12-well plate. Cells were left to expand in expansion medium for a further 3 days prior to nucleofection (6 days in total) prior to reprograming. On day 6, medium containing the cells was collected into a 15ml falcon tube. 200,000 cells were transferred into a new tube with 12ml of PBS and centrifuged at 300g for 5 minutes. Lonza P3 Nucleofection supplement was added to P3 buffer (P3 buffer), from P3 Primary Cell 4D-Nucleofector™ X Kit L (Lonza™), according to manufacturer instruction (20µl/electroporation reaction). Addgene episomal plasmids pCXLE-hUL [pCXLE-hUL was a gift from Shinya Yamanaka (Addgene plasmid #27080; http://n2t.net/addgene:27080; RRID:Addgene_27080)], pCXLE-hSK [pCXLE-hSK was a gift from Shinya Yamanaka (Addgene plasmid #27078; http://n2t.net/addgene:27078; RRID:Addgene_27078)] and pCXLE-hOCT3/4-shp53-F [pCXLE-hOCT3/4-shp53-F was a gift from Shinya Yamanaka (Addgene plasmid #27077; http://n2t.net/addgene:27077; RRID:Addgene_27077)](Okita et al., 2007) were added to P3 buffer (0.33 µg each). Following centrifugation, the cell pellet was resuspended in the P3 buffer containing Yamanaka's plasmids. Cells were then transferred to the cuvette strip (Lonza™) and electroporated using program EO-115 of Amaxa 4D-Nucleofector™ System (Lonza). After electroporation cells were kept at room temperature for 5 minutes. 80µL of Roswell Park Memorial Institute (RPMI) 1640 (Gibco™) was added to the cuvette strip and it was placed at 37°C for 10 minutes. Cells were then added to 1 well of a 6-well plate (Corning®Costar), previously coated with Geltrex (1:100), in 2ml of EM. Cells were kept in EM until day 8. From day 8 to day 10 cells were fed daily with a 1:1 mix of Essential 8™ Medium (E8 Medium; Invitrogen) and EM. After day 10 cells were fed daily with E8 Medium. Retinal differentiation of patient iPSC cells was performed as described above.

Production and use of recombinant AAV viral vector

Both pD10/RK*promoter-GFP* and pD10/RK*promoter-RPGR,* construct containing AAV-2 inverted terminal repeat (ITR) were used to generate AAV7m8.RK.GFP and RK.RPGR viral vector. Recombinant AAV2/2 serotype particles were produced through a previously described triple transient transfection method HEK293T cells (Nishiguchi et al., 2015). AAV7m8 serotype was bound to an AVB Sepharose column (GE Healthcare) and eluted with 50 mM Glycine pH2.7 into 1 M Tris pH 8.8. Vectors were washed in 1 × PBS and concentrated to a volume of 100–150 µl using Vivaspin 4 (10 kDa) concentrators. Viral genome titres were determined by quantitative real-time PCR using a probe-based assay binding the SV40 poly-adenylation signal. Amplicon-based standard series of known amounts were used for sample interpolation. Final titres were expressed as vg/mL.
SV40 Forward primer: 5'-Agcaatagcatcacaaatttcacaa-3'.
SV40 Reverse primer: 5'-AGATACATTGATGAGTTTGGACAAAC-3'.

SV40 Probe: FAM-5'-AGCATTTTTTTCACTGCATTCTAGTTGTGGTTTGTC-3'-TAMRA. Retinal organoids were infected at 15-18 wks with 3×10^{11} viral particles per organoid, with an estimated gMOI of 6×10^{5}.

Immunohistochemistry and immunocytochemistry

hPSC-derived retinal organoids (n>20 NRVs; N=3 independent experiments) were used for assessments of ALT media and OS formation as described in the main manuscript. Retinal organoids were collected either unfixed (RPGR antibodies) or fixed for 1 hour in 4% paraformaldehyde (PFA) and embedded in OCT (RA Lamb). Cryosections were cut (12-14 µm thick) and all sections were collected for analysis. For immunohistochemistry, sections were blocked in 5% goat or donkey serum and 1% bovine serum albumin in PBS. Primary antibody (**Table S3**) was incubated overnight at 4°C. Sections were incubated with secondary antibody for 2 hrs at RT, washed and counter-stained with DAPI (Sigma-Aldrich). Alexa fluor 488, 546 and 633 secondary antibodies (Invitrogen-Molecular Probes) were used at a 1:500 dilution.

For immunocytochemistry of whole retinal organoids (3D view) a clearing protocol was performed. Briefly, NRVs were fixed for 1 hour in 4% PFA. Samples were blocked as above, including 0.3% Triton X-100 in PBS, and primary antibody was incubated overnight at 4°C. Samples were incubated with secondary antibody and DAPI overnight at 4°C. Samples were dehydrated in a graded ethanol series (30, 50, 70, 80, 96, and 2 x 100% ethanol in PBS), and transferred into clearing solution (2 parts benzylbenzoate (Sigma-Aldrich):1 part benezylalcohol (Sigma-Aldrich) for 20 min in the dark. Secondary antibodies were used at a 1:300 dilution.

Confocal image acquisition and quantification

Images were acquired by confocal microscopy (Leica DM5500Q). A series of XY optical sections, approximately 0.8µm apart, throughout the depth of the section were taken and built into a stack to give a projection image. LAS AF image software was used.

To determine the organisation of photoreceptors within mESC-derived 3D retinal differentiation cultures, cryoembedded EBs (x6 EBs/sample) were sectioned (18 µm), stained for Recoverin and mounted. As the quantity of organised retinal regions can vary greatly between embryoid bodies and batches of differentiation we analysed at least 12 EBs over multiple independent differentiations (n>3) using several mouse ES cell lines (N=3), to quantify the presence of organised ONL-like photoreceptor layers. Every sixth section through the EBs was examined for the presence of at least one region of Recoverin+ photoreceptors, if this was not present the section was classed as non-retinal (Figure S1A,E). If Recoverin+ photoreceptors were present, the sections were further examined for the presence of an ONL-like layer using DAPI (Figure S1F,G; ONL-like layer indicated with white dashed lines). Recoverin+ segment structures extending from the ONL-like layer could also be observed, further confirming a region of organised photoreceptors (Figure S1B,C,F,G; white arrow heads). If Recoverin+ cells were present but there was no indication of any ONL-like regions observed with DAPI, this section was classed as disorganised photoreceptors (Figure S1D,H). All analysis was performed blinded.

Human retinal organoids were cryoembedded before sectioning and mounting. PHPR2+ OS structures were located using epifluorescence illumination and measures were taken from 10 regions of the neuroepithelia in 10 images using FiJi software. To accurately measure segment length, only regions showing aligned sagittal sections through the segments were used, as determined by Phalloidin expression in the OLM and Mitochondria staining in the ISs.

For pixel intensity quantification a threshold was set that separated the fluorescent signal from background. All images were analysed using the same threshold value and presented as the percentage of positive pixels above threshold. All other aspects of imaging were kept consistent. For the quantification of RPGR and RHODOPSIN, the the CC region and the ONL were selected for analysis, respectively. For the quantification of GFAP, the whole image was used. Image analysis was performed blinded.

Ultrastructural analysis

For ultrastructural analysis, mESC-derived EBs or hPSC-derived retinal organoids were fixed in 3 % glutaraldaehyde and 1 % paraformaldehyde in 0.08M sodium cacodylate-HCl (pH 7.4), for at least 12 hrs at 4ºC. The specimens were washed with 2.5% glutaraldehyde in 0.1M cacodylate-HCl buffer (pH 7.4) and post-fixed in 1 % aqueous solution of osmium tetroxide for 2.5 hrs in the dark. The specimens were then dehydrated by an ascending ethanol series (50 - 100 %, 10 mins per step with rotation) with additional 2 x 10 mins 100 % ethanol and 3 x 10 mins 1,2-epoxypropane steps. The specimens were left in a 50:50 mixture of 1,2-epoxypropane and araldite for a minimum of 3 hrs with rotation, followed by 5 hrs in fresh araldite and embedded and cured at 60 ºC for 48 hrs. Semithin (0.7 µm) and ultrathin (70 - 100 nm) sections were cut using a Leica ultracut S microtome fitted with an appropriate diamond knife (Diatome histoknife Jumbo or Diatome Ultrathin). Semithin sections were stained with 1 % toluidine blue and imaged using a Leitz Diaplan microscope fitted with a Leica digital camera (DC 500). Ultrathin sections were collected on copper formvar coated grids (100 mesh, Agar Scientific) and imaged both with and without 1% lead citrate contrast-staining on a JEOL 11010 TEM operating at 80V. Images were acquired with a Gatan Orius camera and montaged using Digital Micrograph software. Several montaged images were stitched together using Adobe Photoshop elements 12 software and cropped to produce the final figure panels.

For scanning electron microscopy 1 and 2 µm thick semithin sections were cut and dried onto 20 mm glass coverslips, etched for 30 minutes in potassium methoxide (Sigma Cat 60402-250 ml), rinsed twice for five minutes in methanol followed by hexamethyldisilazane (HMDS) and air drying. Dried specimens were coated with 1.5 nm of platinum and imaged in a Zeiss Sigma FESEM operating at 3 – 5kV.

Serial Block-Face Scanning Electron Microscopy (3view EM)

hPSC-derived retinal organoids were fixed in 3% glutaraldehyde and 1% paraformaldehyde in 0.08 M sodium cacodylate –HCl buffer, pH 7.4, and then en bloc stained with osmium ferricyanide-thiocarbohydrazide-osmium, uranyl acetate and Walton’s lead citrate with two modifications. First, the osmium concentration was reduced to 1% and, second, graded alcohols (50, 70, 90, 3 x 100%) and propylene oxide were used instead of acetone to dehydrate specimens for infiltration and curing overnight at 60°C in Durcupan ACM resin. Specimens were then superglued to aluminium pins and trimmed to place the region of interest within a 0.5 x 0.5 x 0.4 mm mesa and sputter coated with 5 nm gold palladium. Stacks of backscatter electron micrographs were automatically acquired using a Gatan 3 view system working in conjunction with a Zeiss Sigma field emission scanning electron microscope working in variable pressure mode at a chamber pressure of 9 Pa and 4 kV. At a standard magnification of x1000 and a pixel resolution of 4096 x 4096, the total area sampled measured 255.4 µm2 on x and y and, depending on the number of 100 nm thick sections sampled, between 67 and 150 µm on z. The resulting stacks were normalised for contrast and brightness and then converted to TIFF images in Digital Micrograph software prior to importation into Amira 5.3.3 software for semi-automated segmentation and presentation.

Quantitative PCR

50 ng of cDNA was loaded per well of 96-well plate (Life Technologies Ltd., UK) mixed with 2x Fast Start TaqMan® Probe Master Mix (Roche Ltd., UK), gene-specific forward and reverse primers at a final 900 nM concentration and an appropriate hydrolysis probe binding to the amplified region at a final concentration of 250 nM (Roche Diagnostics Ltd., UK), all dissolved in DNase and RNase free water up to 20 µl final volume. Each cDNA sample was run in triplicate. The reactions were then run on an ABI Prism 7900HT Fast Real-time Sequence Detection System (Applied Biosystems Ltd., UK) equipped with SDS 2.2.2 software for amplication results analysis. From amplification curves Ct values were obtained for each sample. Expression levels were normalized to photoreceptor specific *Crx* (*Crx* gene) mRNA levels for each sample to assess relative expression of particular genes in different experimental conditions. Cycling conditions were as follows 40 cycles of 95°C for 30 sec. and 60°C for 1 minute. **Table S4** contains the list of gene-specific primer sequences used.

Flow cytometry analysis

Mouse ESC-derived retinal vesicles were dissociated at various time points of culture into a single cell suspension using a papain-based Neurosphere Dissociation Kit (Miltenyi Biotec, 130-095-943). Cells were counted and resuspended in 1% Bovine Serum Albumin (in PBS) to a concentration of 1×10^7 cells per mL, and CD73 antibody staining (Miltenyi Biotec, 130-103-054) was performed in 100µL aliquots and incubated for 30 minutes at 4°C at a dilution of 1:100. Cells were washed once in 1X Binding Buffer and resuspended in PBS. SYTOX Blue (Invitrogen) was then added to the samples at a final concentration of 1µM for 5 minutes at room temperature before analysis. Cells were analysed using FACSDiva software. Background fluorescence was measured using unstained cells and single-stained controls were used to set gating parameters between positive and negative populations (see **Figure S2**). Small debris, cell fragments and aggregates were excluded from analysis on the basis of live-dead dye fluorescence followed by forward and side scatter (measuring cell size and granularity, respectively).

Statistical analysis

All means are presented ± SD (standard deviation), unless otherwise stated; N, number of independent differentiations or experiments performed; n, number of images, sections, EBs or organoids examined, where appropriate. For quantification assessment by Flow cytometry statistical analysis is based on at least three independent experiments. Statistical significance was assessed using Graphpad Prism 6 software and denoted as $^*p < 0.05$, $^{**}p < 0.01$, $^{***}p < 0.001$ and $^{****}p < 0.0001$. Appropriate statistical tests were applied including ANOVA with relevant post hoc correction for multiple comparisons; Kruskal-Wallis with Dunn's multiple comparisons test; two-tailed Mann-Whitney test.

Supplemental References

Nishiguchi, K.M., Carvalho, L.S., Rizzi, M., Powell, K., Holthaus, S.-M. kleine, Azam, S.A., Duran, Y., Ribeiro, J., Luhmann, U.F.O., Bainbridge, J.W.B., et al. (2015). Gene therapy restores vision in rd1 mice after removal of a confounding mutation in Gpr179. Nature Communications *6*, 6006.

Okita, K., Ichisaka, T., and Yamanaka, S. (2007). Generation of germline-competent induced pluripotent stem cells. Nature *448*, 313–317.

Osakada, F., Ikeda, H., Sasai, Y., and Takahashi, M. (2009). Stepwise differentiation of pluripotent stem cells into retinal cells. Nature Protocols *4*, 811–824.
