# Supplementary material for: Antioxidant and lipid supplementation improve the development of photoreceptor outer segments in pluripotent stem cell-derived retinal organoids
Source: Stem Cell Reports. 2022 Mar 24;17(4):775–88. doi: 10.1016/j.stemcr.2022.02.019 (PMC9023802; doi:10.1016/j.stemcr.2022.02.019)
Supplement: Document S2. Article plus supplemental information [file mmc3.pdf]

Stem Cell Reports
Article
OPEN ACCESS

Antioxidant and lipid supplementation improve the development of photoreceptor outer segments in pluripotent stem cell-derived retinal organoids

Emma L. West,[1,4] Paromita Majumder,[1] Arifa Naeem,[1] Milan Fernando,[1,5] Michelle O'Hara-Wright,[1,5] Emily Lanning,[1,4] Magdalena Kloc,[1] Joana Ribeiro,[1] Patrick Ovando-Roche,[1] Ian O. Shum,[1] Neeraj Jumbu,[1] Robert Sampson,[1] Matt Hayes,[1] James W.B. Bainbridge,[1,2] Anastasios Georgiadis,[1] Alexander J. Smith,[1,4] Anai Gonzalez-Cordero,[1,5,*] and Robin R. Ali[1,2,3,4,*]

[1]UCL Institute of Ophthalmology, 11-43 Bath Street, London EC1V 9EL, UK
[2]NIHR Biomedical Research Centre at Moorfields Eye Hospital NHS Foundation Trust and UCL Institute of Ophthalmology, City Road, London EC1V 2PD, UK
[3]Kellogg Eye Center, University of Michigan, 1000 Wall Street, Ann Arbor, MI 48105, USA
[4]Present address: KCL Centre for Cell and Gene Therapy, Tower Wing, Guy's Hospital, London SE1 9RT, UK
[5]Present address: Children's Medical Research Institute, University of Sydney, 214 Hawkesbury Road, Westmead NSW 2145, Australia
*Correspondence: agonzalez-cordero@cmri.org.au (A.G.-C.), robin.ali@kcl.ac.uk (R.R.A.)
https://doi.org/10.1016/j.stemcr.2022.02.019

SUMMARY

The generation of retinal organoids from human pluripotent stem cells (hPSC) is now a well-established process that in part recapitulates retinal development. However, hPSC-derived photoreceptors that exhibit well-organized outer segment structures have yet to be observed. To facilitate improved inherited retinal disease modeling, we determined conditions that would support outer segment development in maturing hPSC-derived photoreceptors. We established that the use of antioxidants and BSA-bound fatty acids promotes the formation of membranous outer segment-like structures. Using new protocols for hPSC-derived retinal organoid culture, we demonstrated improved outer segment formation for both rod and cone photoreceptors, including organized stacked discs. Using these enhanced conditions to generate iPSC-derived retinal organoids from patients with X-linked retinitis pigmentosa, we established robust cellular phenotypes that could be ameliorated following adeno-associated viral vector-mediated gene augmentation. These findings should aid both disease modeling and the development of therapeutic approaches for the treatment of photoreceptor disorders.

INTRODUCTION

Inherited retinal degenerations caused by mutations in photoreceptor-specific genes result in blindness for millions of people worldwide, with limited treatment options currently available. A fundamental requirement for the development of novel retinal therapies is the establishment of robust pre-clinical models in which disease mechanisms can be elucidated and new therapies tested. One approach is the use of human pluripotent stem cells (hPSCs), which can be differentiated toward retinal lineages. Since the initial demonstration of ESC-derived optic cups that develop into layered retinal tissue, many groups have devised protocols to generate 3D retinal structures, commonly referred to as retinal organoids (reviewed in O'Hara-Wright and Cordero, 2020). In turn, this has led to an increase in disease modeling using induced pluripotent stem cells (iPSCs) derived from patients carrying photoreceptor-specific mutations (Parfitt et al., 2016; Deng et al., 2018; Megaw et al., 2017; Lane et al., 2020; Gao et al., 2020; Lukovic et al., 2020). However, a remaining limitation is the incomplete maturation of hPSC-derived photoreceptors *in vitro*. To date, only a few rudimentary outer segment (OS)-like structures have been observed by ultrastructural analysis, following extensive long-term culture (Lukovic et al., 2020; Wahlin et al., 2017; Gonzalez-Cordero et al., 2017; Ovando-Roche et al., 2018). The photoreceptor OS is a specialized sensory cilium, formed of stacked membranous discs that are constantly renewed and contain the photoresponsive opsins (Young, 1969). Therefore, improved formation of hPSC-derived photoreceptors bearing OSs is essential for effective disease modeling, as these structures are vital for photoreceptor function.

The increased metabolic activity of mature photoreceptors is maintained *in vivo* by the high rate of choroidal blood flow and the supportive function of the retinal pigment epithelial (RPE) cells (Boulton and Dayhaw-Barker, 2001; Strauss, 2005). RPE cells phagocytose shed OSs, recycling both visual pigments and essential fatty acids, the two major components of the disc membranes (Rice et al., 2015; Bazan et al., 1992). By considering the high concentrations of essential nutrients maintained in the outer retina, we developed an optimized medium for the long-term culture of maturing photoreceptors. We also investigated supplementation with docosahexaenoic acid (DHA), the predominant long chain poly-unsaturated fatty acid (LC-PUFA) present in the retina, which is essential for correct OS disc morphology

This is an open access article under the CC BY-NC-ND license (http://creativecommons.org/licenses/by-nc-nd/4.0/).

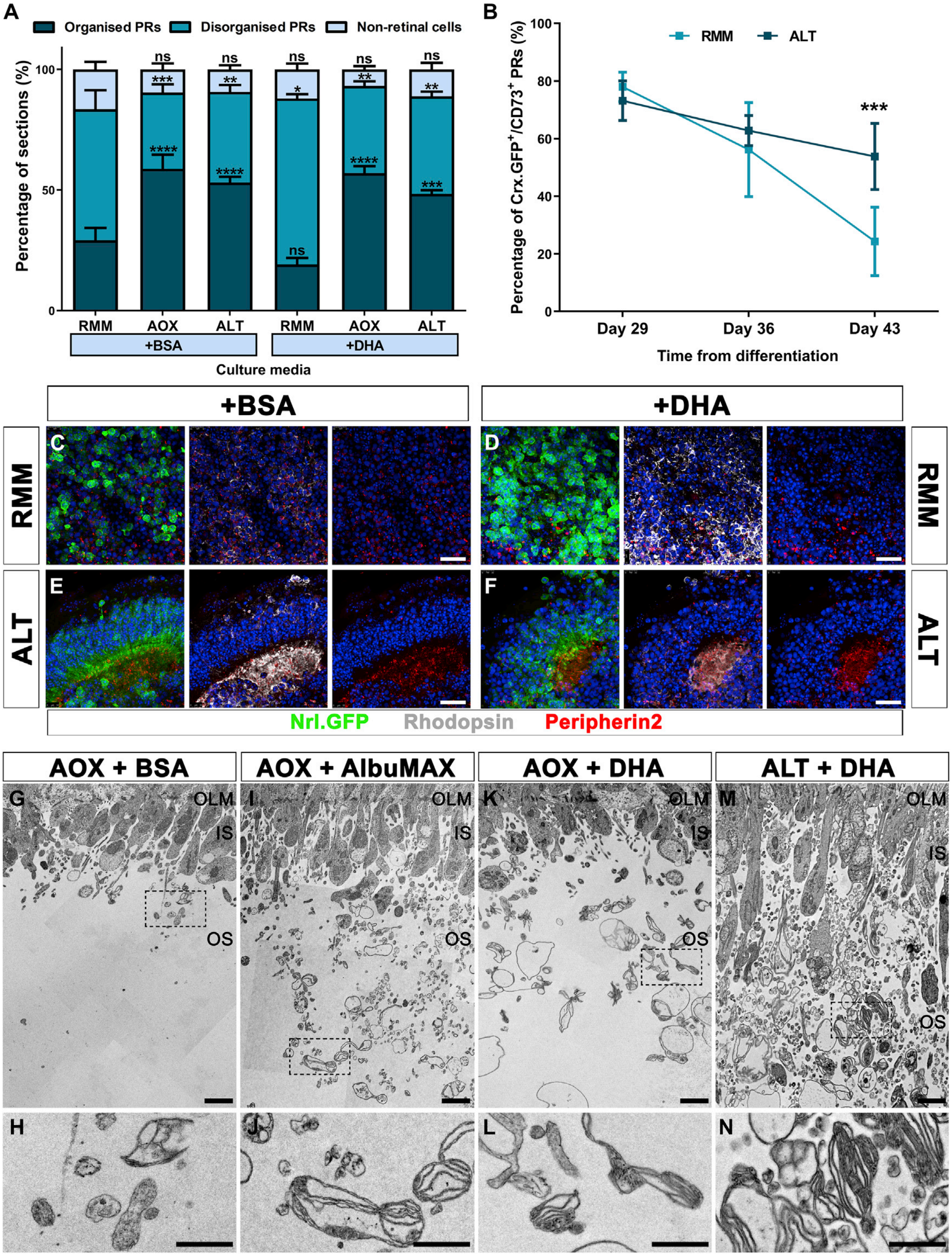

Figure 1. Improved long-term culture of mouse ESC-derived photoreceptors

(A) Histogram showing the percentage of classified sections (non-retinal, organized, and disorganized PRs; see Figure S1 and supplemental experimental procedures for criteria) when cultured under various conditions (±SEM; *p < 0.05, **p < 0.01, ***p < 0.001, ****p < 0.0001; n ≥ 12 sections, N ≥ 3 experiments).

(B) Line graph showing the percentage of rod ($Crx.GFP^+CD73^+$) PRs present over time in RMM or ALT media (±SD; ***p < 0.001; n = 24 pooled EBs, N ≥ 3 experiments).

(legend continued on next page)

and optimal visual function (Shindou et al., 2017). In addition, we examined whether our enhanced culture conditions might allow improved retinal disease modeling. We therefore generated iPSC lines from patients with X-linked retinitis pigmentosa type 3 (XLRP3) caused by pathogenic mutations in the retinitis pigmentosa GTPase regulator (*RPGR*) gene. *RPGR* mutations result in defective photoreceptor cilial function and cause a particularly severe type of RP with an early onset of disease in childhood and relatively rapid progression that leads to severe visual impairment by the third to fourth decade (Tee et al., 2016). We were able to observe cellular defects in XLRP3 hPSC-derived retinal organoids that subsequently allowed us to evaluate whether a shortened *RPGR* transgene was able to rescue function in human photoreceptors.

RESULTS

Generation of mouse ESC-derived photoreceptor cells bearing outer segment-like structures

To promote the formation of photoreceptor OSs, not previously observed in late-stage mouse embryonic stem cell (mESC)-derived retinal cultures, we designed an enhanced serum-free medium, herein referred to as ALT. To determine the importance of individual components of ALT medium, we also tested an antioxidant-rich medium, hereafter referred to as AOX medium, in addition to our original retinal maturation medium (RMM); see Table S1 for media composition. In parallel, we supplemented with 50 μM DHA pre-complexed with fatty acid-free BSA (referred to as +DHA). BSA alone was used as a control (+BSA). The differentiation of mESC-derived embryoid bodies (EBs) containing retinal regions was established as previously described, with retinal cultures maintained in long-term media from day 21 onward (Kruczek et al., 2017).

The first sign of mESC-derived photoreceptor cell loss after day 30 is the disorganization of the outer nuclear layer (ONL)-like structures within the EBs. We therefore examined day 34 cryosections, according to set criteria (Figures S1A–S1H; see supplemental experimental procedures) and determined the percentage of sections with organized, disorganized, or no photoreceptors (non-retinal cells) following culture with the various media (Figure 1A). A significantly greater percentage of sections containing organized photoreceptors was observed with AOX and ALT media, compared with those maintained in RMM with BSA (Figure 1A; 59 ± 10% and 53 ± 6% versus 29 ± 12% of sections, respectively; mean ± SEM, $p < 0.0001$; two-way ANOVA with Dunnett's multiple comparisons test; $n \geq 12$ sections, $N \geq 3$ differentiations). This suggests that the increased antioxidants, present in both AOX and ALT media, supported the maintenance of photoreceptor organization and enhanced survival at this later developmental timepoint. A similar result was observed for AOX and ALT media with DHA supplementation, compared with the RMM +BSA (Figure 1A, 57 ± 5% and 48 ± 4% versus 29 ± 12% of sections, respectively; mean ± SEM, $p < 0.001$). However, significantly more sections contained disorganized photoreceptors in RMM +DHA, suggesting a detrimental effect of increased lipid concentrations without additional antioxidants (Figure 1A; 69 ± 4% versus 54 ± 18% of sections, respectively; mean ± SEM, $p < 0.05$). No significant differences were found in the percentage of sections containing non-retinal cells for any of the media conditions (Figure 1A; mean ± SEM, $p > 0.05$).

To determine if there was increased survival of photoreceptors over time, we used flow cytometry to analyze the percentage of rod photoreceptors (Crx.GFP$^+$CD73$^+$ cells), using a Crx.GFP mESC line (Figures 1B and S1I). At day 29, no significant difference in the percentage of rods grown in either RMM or ALT media only was observed, confirming differentiation was unaffected (Figure 1B; 73 ± 7% versus 78 ± 5% Crx.GFP$^+$CD73$^+$ cells in ALT and RMM, respectively; $p > 0.05$; two-way ANOVA with Sidak's MCT; $n = 24$ pooled EBs, $N \geq 3$ experiments). At day 36 and 43, the percentage of rods was greatly reduced when cultured in standard RMM. Despite a slight reduction over time, use of ALT medium resulted in a significantly higher percentage of rods being preserved at day 43 (Figure 1B; 54 ± 12% versus 24 ± 12% Crx.GFP$^+$CD73$^+$ cells, respectively; $p < 0.001$). These results therefore support the use of this enriched base medium for extended preservation of mESC-derived photoreceptors at late stages of culture.

To look in more detail at the morphology of photoreceptors, cryosections were stained for phototransduction proteins rhodopsin and peripherin, which localize to the OS in the adult mouse retina (Figures 1C–1F and S2). As previously observed, rhodopsin and peripherin staining was present in the segment region of the photoreceptors under standard conditions (RMM +BSA), with rhodopsin also present in the ONL-like layer (Figures S2A and S2G and Gonzalez-Cordero et al., 2013). Similar results were

(C–F) Representative images of mESC-derived retinal regions (Nrl.GFP line, green rod PRs) at day 42, maintained in either RMM or ALT media, with +BSA or +DHA and stained for rhodopsin (gray) and peripherin2 (red).
(G–N) TEM micrographs showing retinal regions maintained in different culture conditions. Inset regions shown at higher magnification (H, J, L, and N). Nuclei were stained with DAPI (blue). Scale bars, 1μm (H, J, L, and N), 2μm (G, I, K, and M), and 10μm (C–F).
IS, inner segment region; OLM, outer limiting membrane; OS, outer segment region; PRs, photoreceptors.

observed for all other media conditions (Figures S2C, S2E, S2I, and S2K). However, slightly less rhodopsin was noted in the ONL-like layer for both AOX and ALT media conditions with lipid-bound BSA, for all mESC lines examined (Figures S2D, S2F, S2J, and S2L and data not shown). This could be clearly observed at day 42, whereby rhodopsin was discretely localized to the segment region of ALT-grown photoreceptors; in contrast, no organized photoreceptor regions remained under standard conditions, as observed in previous studies (Figures 1C–1E, respectively and Gonzalez-Cordero et al., 2013). This rhodopsin localization is reminiscent of late postnatal (>P12) mouse photoreceptors, a developmental stage that until now had not been possible to investigate in 3D mESC-derived retinal cultures. Further testing of individual components in AOX medium revealed no significant differences in photoreceptor organization, compared with the AOX +BSA control (Figures S2M–S2Q, mean ± SEM, $p > 0.05$; two-way ANOVA with Dunnett's MCT; $n \geq 12$ sections, $N \geq 3$ differentiations).

To investigate the formation of OS-like structures further, transmission electron microscopy (TEM) was used to examine day 34 mESC-derived photoreceptors (Figures 1G–1N). Despite preservation of photoreceptor organization with AOX +BSA medium, there was little indication of OS-like structures at the ultrastructural level, similar to previous observations using standard conditions (Figures 1G and 1H) (Gonzalez-Cordero et al., 2013). In contrast, numerous membranous structures were observed with the addition of lipid-rich BSA (AlbuMAX) and BSA-bound DHA (Figures 1I and 1L, respectively). Likewise, membranous structures were abundant in ALT +DHA retinal cultures, with many structures containing numerous internal foldings, reminiscent of nascent OSs (Figures 1M and 1N). These findings demonstrate the importance of additional antioxidants to maintain photoreceptors in long-term mESC-derived retinal cultures, as well as the requirement of BSA-bound lipids for the development of OS-like structures.

Efficient generation of human PSC-derived photoreceptors bearing outer segment-like structures

Having established the benefits of ALT medium for mESC-derived photoreceptors, we next sought to investigate its use for hPSC-derived retinal organoids. A summary of our standard and optimized protocol is shown schematically in Figure 2A. Briefly, hPSC-derived neuroretinal vesicles (NRVs) were isolated from confluent cultures at 3–5 weeks and grown in suspension for up to 10 weeks to form retinal organoids, in accordance with our original protocol (Figure 2A, see methods for detailed protocol). Organoids were further cultured from 12 weeks with either our standard RDM90 or ALT medium, supplemented with either BSA-bound DHA (+DHA) or fatty acid-free BSA (+BSA) as the control. As previously described (Gonzalez-Cordero et al., 2017), using our standard medium supplemented with BSA (RDM90 +BSA), bright field images of retinal organoids revealed a semi-translucent laminated neuroepithelium with brush-like protrusions, herein referred to as a brush border (Figure 2B, white arrowhead). However, a more protuberant brush border was observed in retinal organoids cultured with ALT medium (Figures 2C and 2D, white arrowheads). No differences between ALT +BSA and ALT +DHA were evident, and RDM90 supplemented with DHA was not sufficient to induce the dense brush border observed with ALT medium (Figures S3A–S3F). To examine the brush border in more detail we analyzed semithin sections, which verified this region comprised segment-like structures protruding from the neuroepithelial layer (Figures 2E–2G). Photoreceptor OS formation was further confirmed in all conditions by the presence of PERIPHERIN-2 positive (Figures 2H–2J; PRPH2+; red) structures observed apically to the mitochondria-rich inner segments (Figures 2H–2J; MITOCHONDRIA+; green) and the outer limiting membrane (OLM), delineated by phalloidin (Figures 2H–2J; Phalloidin+; magenta). In addition, immunohistochemical analysis demonstrated the presence of ABCA4, a transmembrane phospholipid-transporting ATPase present in the membranes of OSs *in vivo*, localized apically to ESPIN, a connecting cilium (CC) marker (Figures S3G and S3H). Transcriptional analyses also confirmed the increased expression of phototransduction components *RHO* and *ABCA4* for organoids maintained in ALT medium, compared with RDM90 (Figure 2K).

To determine if the dense brush borders observed were due to the increased efficiency of OS-like structure formation, the ratio of OS-like to inner segment structures was quantified and expressed as a percentage. The percentage of inner segments that had OS-like structures was significantly greater for both ALT +BSA (68 ± 43%) and ALT +DHA (83 ± 22%) compared with RDM90 +BSA (26 ± 14%) cultures (Figure 2L; $p < 0.01$; Kruskal-Wallis with Dunn's MCT; $n = 30$ images, $N = 3$ differentiations). In addition, the length of the segments, from the Phalloidin+ OLM to the distal edge of the PRPH2+ OS-like structures, was measured. Significantly longer segments were observed for both ALT conditions (21 ± 2 μm and 27 ± 2 μm for +BSA and +DHA, respectively), compared with RDM90 +BSA (17 ± 2 μm) (Figure 2M; $p < 0.05$; ANOVA with Tukey's MCT; $n = 40$ images, $N = 4$ differentiations). Combined, these results suggest the improved formation of photoreceptor OS-like structures in retinal organoids at 26 weeks, when cultured with ALT medium.

Improved development of hPSC-derived photoreceptor outer segment-like structures with long-term culture in ALT medium

To assess if the enhanced brush borders could be maintained, retinal organoids were cultured for up to 37 weeks.

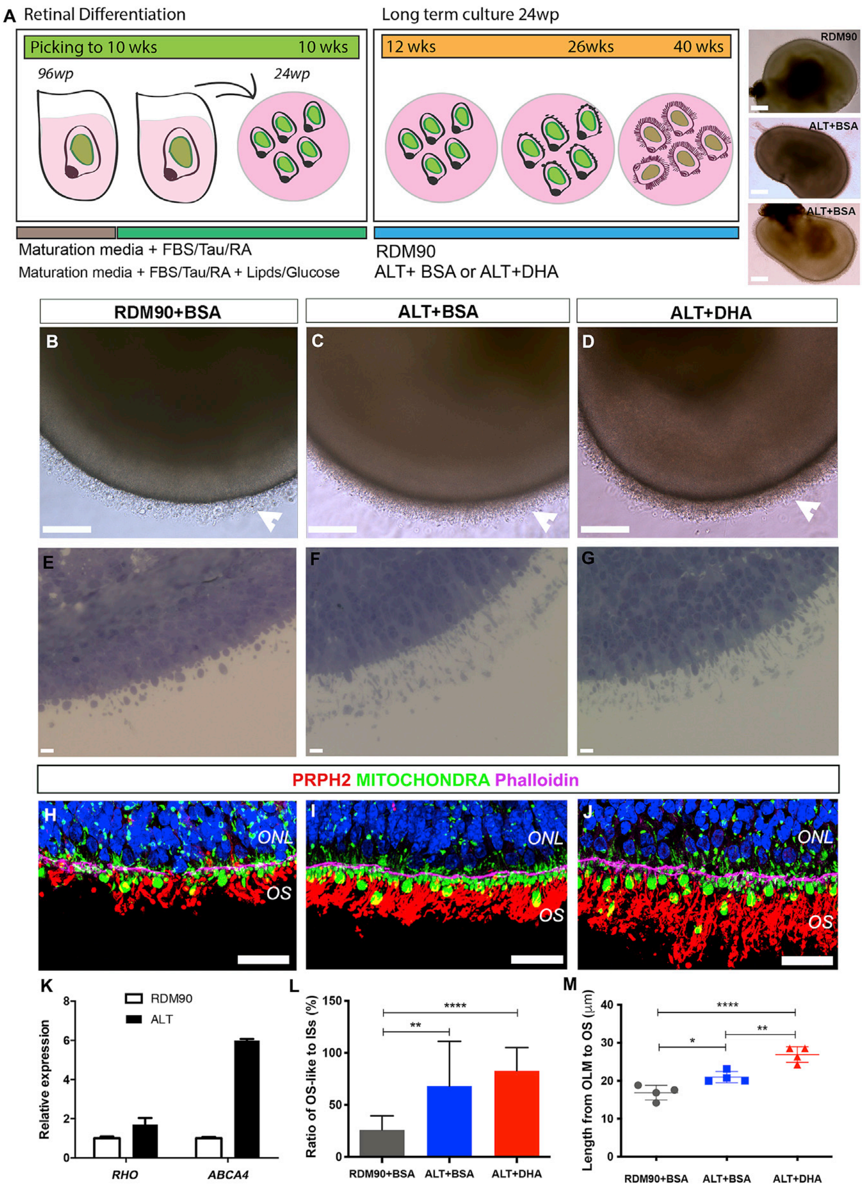

Figure 2. Efficient differentiation of outer segment-bearing photoreceptors in 26-week hESC-derived retinal organoids

(A) Schematic of retinal differentiation protocol with bright field images of retinal organoids.

(B–D) Representative bright field images showing brush borders (white arrowheads).

(E–G) Semithin images of brush border region.

(H–J) Retinal neuroepithelium showing phalloidin positive OLM (pink), mitochondria-rich ISs (green), and elongated PRPH2+ OSs (red).

(K) RT-qPCR analysis showing the relative expression of *RHO* and *ABCA4* in RDM90 and ALT media (±SEM; n = 15 pooled NRVs, N = 3 experiments).

(L) Histogram showing the ratio of OS-like to IS structures as a percentage (±SD; $^{**}p < 0.01$, $^{****}p < 0.0001$; n = 30 images, N = 3 experiments).

(M) Graph showing the length of segment structures for all conditions (±SD; $^{*}p < 0.05$, $^{**}p < 0.01$, $^{****}p < 0.0001$; n = 40 images, N = 4 experiments). Nuclei were stained with DAPI (blue).

Scale bars, 20 µm (E–G), 25 µm (H–J), 50 µm (B–D), 70 µm (A). OLM, outer limiting membrane; IS, inner segment; ONL, outer nuclear layer; OS, outer segment.

A clear difference in gross morphology of the brush border could be observed in both ALT conditions, compared with organoids cultured under standard conditions (Figures 3A–3C). While the inner segments packed with mitochondria and PHPR2+ OS-like structures could be readily identified in all conditions (Figures 3D–3F), significantly longer segment structures were observed for both ALT conditions, compared with RDM90 +BSA (Figure 3G, 37 ± 6 µm +BSA and 40 ± 10 µm +DHA versus 21 ± 3 µm, respectively; $p < 0.05$; ANOVA with Tukey's MCT; n = 40 images, N = 4 differentiations). In addition, the length of the segment region significantly increased from 26 to 35 weeks in ALT-cultured organoids (Figure 3H; 21 ± 2 µm versus 37 ± 6 µm, respectively; p = 0.029; two-tailed Mann-Whitney test; n = 40 images, N = 4 differentiations). This contrasted with the minimal increase in segment length for RDM90 +BSA cultured organoids over the same period (17 ± 2 µm versus 21 ± 3 µm, respectively). To determine the widespread coverage of RHODOPSIN+/PHPR2+ OS-like protrusions, we examined whole retinal organoids (Figures 3I and 3J). While the use of either medium resulted in the presence of OS-like structures at 37 weeks, the DAPI-positive nuclei (blue) were visible through the brush border under standard conditions, but not in ALT-cultured organoids (Figures 3I and 3J, respectively). This improved coverage

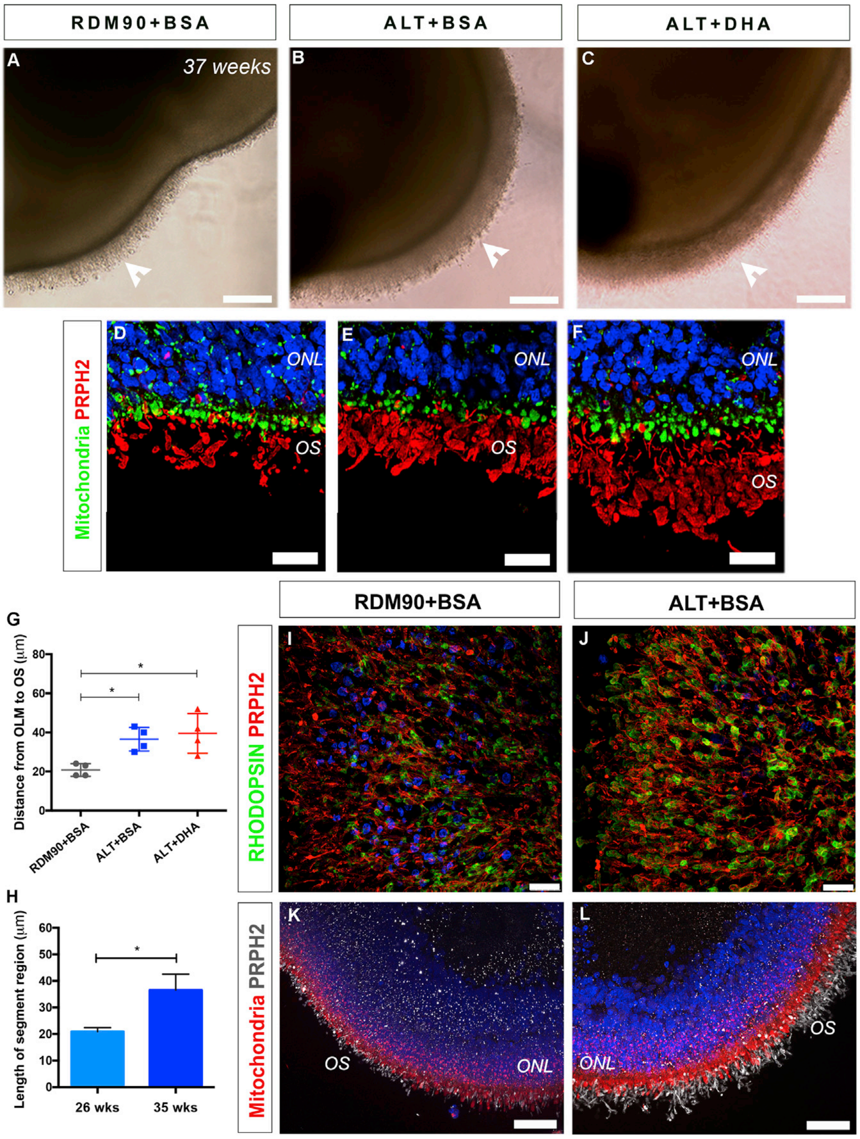

Figure 3. Maintained formation of outer segments by photoreceptors at late stages of culture

(A–C) Representative bright field images of 37-week retinal organoids showing brush borders (white arrowheads).

(D–F) Retinal organoid neuroepithelium showing mitochondria-rich ISs (green) and elongated PHPR2+ OSs (red).

(G) Graph showing the length of segment structures maintained in all media conditions (±SD; $*p < 0.05$; n = 40 images, N = 4 experiments).

(H) Graph showing the segment length in 26- and 35-week ALT cultures (±SD; $*p < 0.05$; n = 40 images, N = 4 experiments).

(I and J) 3D view of a retinal organoid showing the distribution of RHODOPSIN+ (green) and PHPR2+ (red) photoreceptor OSs.

(K and L). Cross-sectional image, showing the mitochondria-rich ISs (red) and PHPR2+ OSs (gray). Nuclei were stained with DAPI (blue).

Scale bars, 25 µm (D, F, I, and J), 50 µm (K and L). ONL, outer nuclear layer; IS, inner segment; OS, outer segment.

was also evident when the organoids were analyzed in cross-section, with defined inner segment (Mitochondria+; red) and OS-like (PHPR2+; gray) structures more apparent in ALT-cultured organoids (Figures 3K and 3L). The results described here were performed using H9 hESC-derived retinal organoids; however, enhanced brush borders were observed in all hPSC lines tested (Figures S3I–S3P; N > 5). These findings support the improved formation and continued development of OS-like structures in hPSC-derived photoreceptors, cultured long term in ALT medium. In addition, supplementation with DHA did not result in any significant improvement, as assessed by light microscopy, beyond that observed with ALT +BSA, at late stages of development.

Improved formation of hPSC-derived cone photoreceptor outer segment-like structures

While previous studies have reported the generation and development of hPSC-derived cone photoreceptors (Zhong et al., 2014; Zhou et al., 2015; Gonzalez-Cordero et al., 2017), the formation of cone OS-like structures has not been described specifically. We therefore sought to establish if ALT medium would improve segment formation in hPSC-derived cone photoreceptors. Cones were present in both media conditions, as demonstrated by cone-specific phototransduction components ARRESTIN3 and L/M OPSIN (Figures 4A and 4B). By 37 weeks a significantly greater percentage of double-positive LM OPSIN+/ARRESTIN3+ cones were observed in ALT +BSA

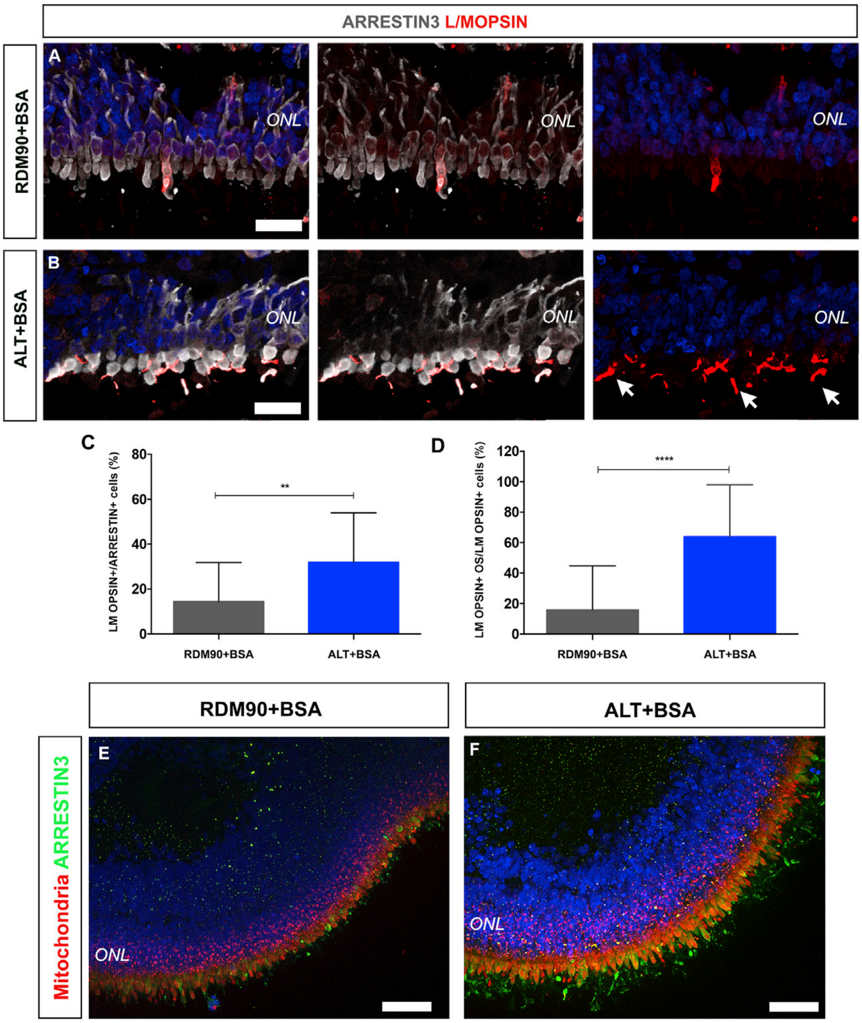

Figure 4. Development of outer segment-bearing cone photoreceptors

(A and B) Representative images showing ARRESTIN3+ and L/MOPSIN+ cone photoreceptors cultured in RDM90 (A) and ALT medium (B). (B) In ALT medium L/MOPSIN staining is localized to outer segments (white arrows).

(C) Histogram showing the percentage of ARRESTIN3+ cones that were also LM OPSIN+ (±SD; $^{**}p < 0.01$; n = 30 images, N = 3 experiments).

(D) Histogram showing the percentage of LM OPSIN+ cones that had LM OPSIN localized to the OS (±SD; $^{****}p < 0.0001$; n = 30 images, N = 3 experiments).

(E and F) Cross-sectional image, showing the mitochondria+ ISs (red) and ARRESTIN3+ cone OSs (green). Nuclei were stained with DAPI (blue).

Scale bars, 25 μm (A and B), 50 μm (E and F). ONL, outer nuclear layer; OS, outer segment; IS, inner segment.

compared with RDM90 +BSA medium (Figure 4C; 32 ± 22% versus 14 ± 17%, respectively; p = 0.0011; two-tailed Mann-Whitney test; n = 30 images, N = 3 differentiations). To determine if this corresponded to improved segment formation, we quantified the percentage of cones showing LM OPSIN+ segment-like structures. While 16 ± 29% of cones demonstrated elongated LM OPSIN+ segment-like structures under standard conditions, this was significantly increased with ALT medium to 64 ± 34% (Figure 4D; p < 0.0001; two-tailed Mann-Whitney test; n = 30 images, N = 3 differentiations). Furthermore, examining whole retinal organoids in cross-section demonstrated the augmented formation of cone OS-like structures (ARRESTIN3+; green), apical to mitochondria-rich inner segments (MITOCHONDRIA+; red), with ALT (Figures 4E and 4F). These results suggest that ALT medium supports enhanced segment formation in cone as well as rod photoreceptor subtypes.

Ultrastructural analysis of hPSC-derived photoreceptors indicates improved outer segment disc structure following culture in ALT medium

The unique structure of photoreceptor OSs, made up of densely packed membranous discs, is crucial for efficient phototransduction. Immunohistochemical analysis of photoreceptors cultured with ALT medium demonstrated elongated axonemes as shown by acetylated-tubulin (cyan), as well as RHODOPSIN- and PHPR2-positive OS-like structures (Figures 5A and 5B, cyan and pink, respectively). To determine the ultrastructure of these extended structures, we examined late-stage retinal organoids by electron microscopy. TEM of photoreceptors maintained in ALT medium confirmed the abundance of structures containing membranous infoldings, reminiscent of OS discs (Figures 5C–5G and S4D–S4K). In contrast, few diffuse and disorganized structures were observed in RDM90 cultures, as described previously (Figures S4A–S4C;

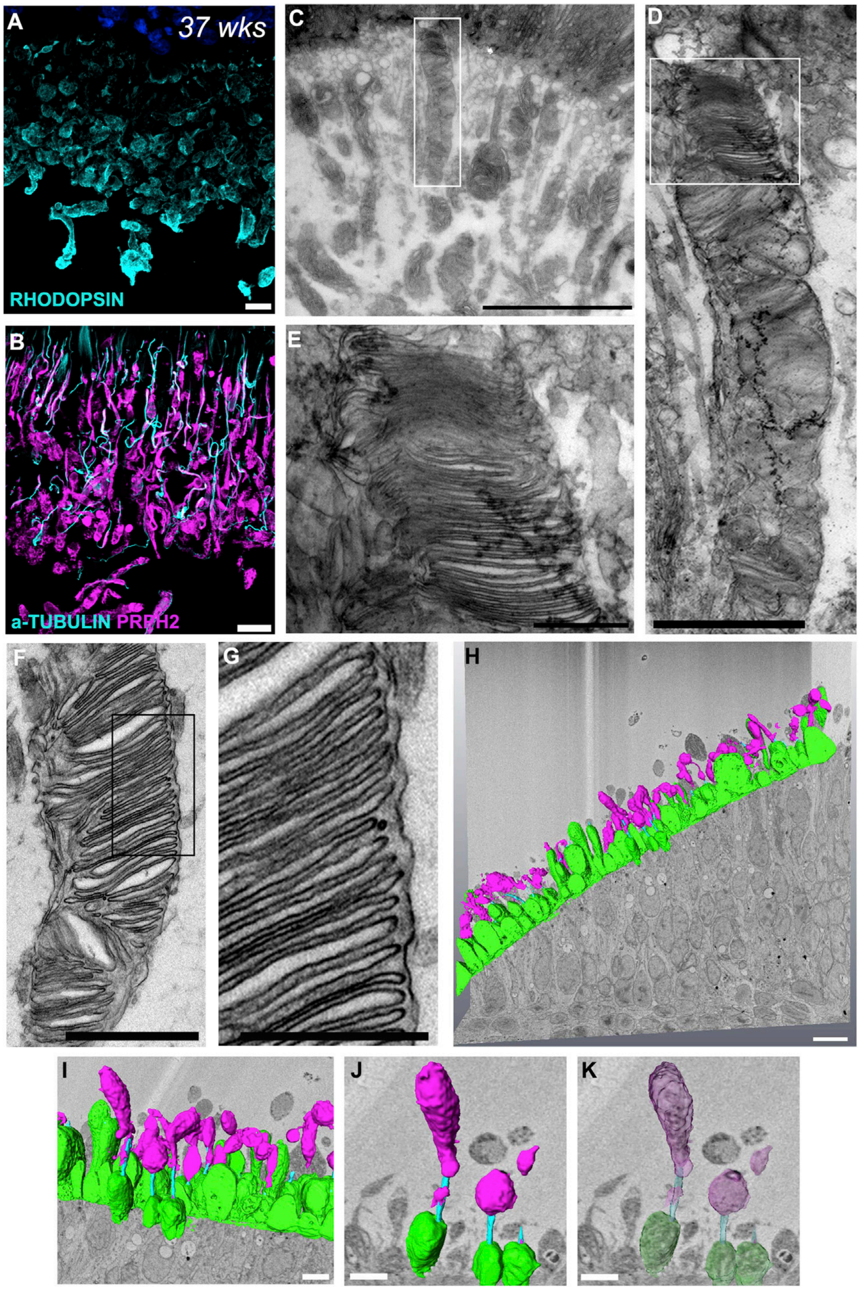

Figure 5. Ultrastructure analysis of photoreceptor cells with improved outer segment formation

(A) Immunohistochemical image of RHODOPSIN+ (cyan) OS regions in ALT maintained photoreceptors.

(B) Image showing the localization of α-TUBULIN+ axonemes (cyan) and PHPR2+ OSs (pink) in ALT-cultured photoreceptors.

(C–E) TEM micrographs of 26-week hPSC-derived photoreceptors cultured in ALT +DHA, showing the abundance of OSs (C). High magnification image of inset from C, showing the morphology of disc membranes (D). High magnification image of inset from D, showing stacked disc membranes (E).

(F and G) Transverse TEM section through one OS (F). High magnification image of inset from (F), showing organized and stacked membranous discs (G).

(H–K) 3view serial 3D reconstruction of the ONL region of an ALT +DHA cultured retinal organoid. The photoreceptor ISs (green), CC (blue), and OSs (pink) were pseudo-colored. Scale bars, 0.5 μm (E and G), 1 μm (F), 2 μm (D),10 μm (A–C). IS, inner segment; CC, connecting cilium; ONL, outer nuclear layer; OS, outer segment.

Gonzalez-Cordero et al., 2017). The OSs of photoreceptors cultured in ALT medium showed discrete membranous structures, with closely stacked disc-like structures clearly present for photoreceptors cultured in ALT +DHA (Figures 5E–5G and S4G–S4K). To confirm the increased abundance of OSs with disc-like morphology following culture in ALT medium, serial block-face scanning electron microscopy (3View) reconstructions of 150 TEM serial sections were performed for all culture conditions (Figures 5H–5K, S5A–S5D and Video S1). In addition, scanning electron microscopy also confirmed an increase in OS formation, following culture in ALT medium (Figures S5E–S5N).

Generation and characterization of RPGR-deficient iPSC-derived retinal organoids

To determine if these enhanced culture conditions improve disease modeling, we investigated the cellular phenotype present in retinal organoids derived from XLRP3 patients with mutations in the RPGR gene. PBMCs were isolated from three XLRP3 patients, and RPGR-deficient iPSCs

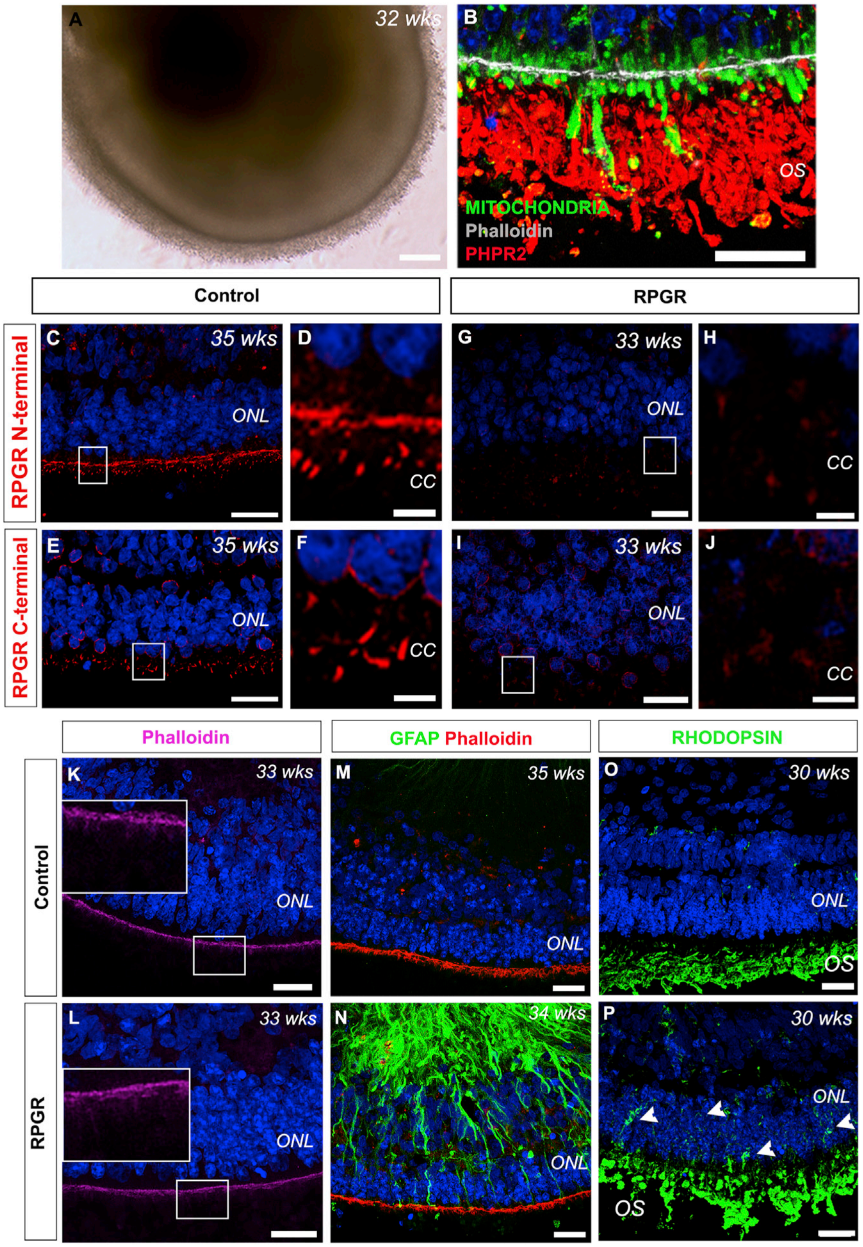

Figure 6. Differentiation and characterization of iPSC-derived RPGR-deficient retinal organoids

(A) Bright field image of an RPGR-deficient retinal organoid showing the brush border.

(B) Image of RPGR-deficient photoreceptors showing mitochondria-rich ISs (green), phalloidin delineated OLM (gray) and PHPR2+ OSs (red).

(C–F) Immunohistochemical analysis with RPGR N-terminal specific antibody (C, D, G, and H) and RPGR C-terminal specific antibody (E, F, I, and J). Images of control retinal organoids, showing typical punctate localization of RPGR (red) to the CC with both antibodies (C–F).

(G–J) Images of RPGR-deficient retinal organoids, showing less punctate staining for the constitutive variant of RPGR (G and H) and an absence of staining for the ORF15 isoform (I and J) in the region of the CC.

(K and L) Representative images of control and RPGR-deficient organoids showing phalloidin (magenta) localized to the OLM. Inset high magnification panels show no differences in staining pattern.

(M and N) Increased GFAP (green) staining in RPGR-deficient neuroepithelia, compared with control. OLM delineated with phalloidin (red) shows no difference.

(O and P) Images showing typical RHODOPSIN staining (green) localized to the OSs in both control and RPGR-deficient rod photoreceptors. Mis-localized RHODOPSIN (green) can also be seen in the cell bodies and processes of RPGR-deficient rods (P, white arrowheads). Nuclei were stained with DAPI (blue).

Scale bars, 5 μm (D, F, H, and J), 25 μm (B, C, E, G, I, and K–P) and 50 μm (A). IS, inner segment; CC, connecting cilia; ONL, outer nuclear layer; OLM, outer limiting membrane; OS, outer segment.

were generated, with all three lines demonstrating typical colony morphology, pluripotency markers, and a normal karyotype (Figure S6). Upon retinal differentiation, all lines gave rise to retinal organoids that were further cultured with our enhanced medium, in addition to three healthy control lines (Figure S6W). Bright field images of 32-week RPGR-deficient retinal organoids demonstrated pronounced brush borders, similar to controls (Figures 6A, 3B and 3C, respectively). Immunohistochemistry confirmed the presence of numerous segment structures apical to the OLM, demarcated by phalloidin (gray), with both mitochondria-rich inner segments (green) and PHPR2+ OS-like structures (red) present (Figure 6B). RPGR protein localizes to the CC in photoreceptors and is thought to be involved with protein trafficking to the OS, with RPGR-deficient mouse models demonstrating opsin mis-localization and photoreceptor degeneration (Hong et al., 2001). To evaluate the presence of the two major RPGR isoforms, RPGR (encoded by 19 exons) and RPGR-ORF15 (terminates within intron 15), we analyzed both N- and C-terminal RPGR binding antibodies, respectively. In control organoids, punctate staining was observed apical

to the edge of the ONL-like layer, in the region of the CC, for both antibodies (Figures 6C–6F). In contrast, little to no staining was observed in this region for RPGR-deficient photoreceptors, with N- and C-terminal specific antibodies, respectively (Figures 6G–6J). We further analyzed RPGR-deficient retinal organoids for characteristics of the disease phenotype. An increase in actin polymerization, as demonstrated by increased phalloidin staining of the CC, has previously been reported in RPGR-deficient cell lines, RPGR KO mice, and recently in RPGR-deficient iPSC-derived retinal organoids (Gakovic et al., 2011; Megaw et al., 2017). However, we could not detect a difference between our control and RPGR-deficient organoids, with a typical staining pattern of the OLM apparent in both (Figures 6K and 6L, insets and Figures S7A, S7C, and S7D). Reactive gliosis, revealed by increased GFAP expression in Müller glia, is a well-described feature of many retinal degenerations. Such a phenotype has been described in both RPGR KO mice and RPGR iPSC-derived retinal organoids previously (Deng et al., 2018; Megaw et al., 2017). Similarly, GFAP (green) upregulation was evident in RPGR-deficient organoids, when compared with controls (Figures 6M and 6N and S7B and S7C). Finally, as RPGR is thought to be involved in transport across the CC, we examined the localization of RHODOPSIN within both healthy and RPGR-deficient photoreceptors. While in control organoids, RHODOPSIN (green) was discreetly localized to the OSs, in RPGR-deficient photoreceptors, RHODOPSIN was also found throughout the cell, including the cell body and processes (Figures 6O and 6P, arrowheads and S7D). Thus our improved culture conditions enable us to distinguish a clearly defined and clinically relevant cellular phenotype in RPGR-deficient retinal organoids.

Characterization of RPGR-deficient iPSC-derived retinal organoids following AAV-mediated gene supplementation

To test the efficacy of RPGR gene supplementation, we used an adeno-associated viral (AAV) vector in which a photoreceptor-specific human rhodopsin kinase (RK) promoter (Khani et al., 2007) was used to express a shortened RPGR-ORF15 transgene (AAV7m8.RK.RPGR). The shortened transgene has been used to rescue a mouse model of XLRP3 (Pawlyk et al., 2016) but had not previously been shown to rescue function in human RPGR-deficient photoreceptors. An AAV vector driving a GFP reporter under the control of the same RK promoter was used as a control (AAV7m8.RK.GFP). Vectors were added to cultures between 15 and 18 weeks and retinal organoids analyzed from 22 weeks, with an estimated transduction efficiency of ~44% of photoreceptors, as determined using the control vector (Figure S7E, 44 ± 11.4% GFP^+ cells; n = 13 sections, N = 5 ROs). First, we determined the presence of RPGR-ORF15 using the C-terminal specific antibody. RPGR-ORF15 protein (red) was present apical to the ONL-like layer in the CC region of RPGR-deficient photoreceptors in RK.RPGR-treated organoids, similar to healthy controls (Figures 7C, 7D, 6E, and 6F, respectively). In contrast, little RPGR-ORF15 protein (red) was detected in the CC region of RK.GFP-treated organoids (Figures 7A and 7B). Quantitative analysis of RPGR-ORF15 staining in the CC region demonstrated a significant increase in RPGR protein from 0.02 ± 0.04% positive pixels in RK.GFP to 0.41 ± 0.31% in RK.RPGR-treated organoids (Figure 7E; $p < 0.05$; Kruskal-Wallis with Dunn's MCT; $n \geq 12$ images, N = 4 experiments). Although the RPGR signals did not reach the levels observed in healthy controls (1.33 ± 0.50% positive pixels), this is most likely due to incomplete transduction of all photoreceptors in the RPGR-deficient retinal organoids.

Having established RPGR-ORF15 supplementation, we examined GFAP upregulation and RHODOPSIN mis-localization in 35-week organoids. While immunohistochemical analysis of control-treated RPGR-deficient retinal organoids revealed GFAP upregulation (red), treatment with RK.RPGR resulted in decreased GFAP levels, similar to healthy controls (Figures 7F, 7G, S7F and S6M, respectively). Quantitative analysis of GFAP staining confirmed a significant decrease in GFAP+ Müller cells in RK.RPGR-treated compared with RK.GFP-treated organoids (Figure 7H; 0.13 ± 0.09% versus 0.99 ± 0.91% positive pixels, respectively; p = 0.005; two-tailed Mann-Whitney test; $n \geq 7$ images, N = 3 experiments). In addition, immunohistochemical analysis of RK.GFP-treated organoids confirmed RHODOPSIN mis-localization to the cell body and processes of photoreceptors, as observed in untreated RPGR-deficient organoids (Figures 7I and 6P, respectively). In contrast, far less RHODOPSIN was observed mis-localized to the ONL-like layer in RK.RPGR-treated mutant photoreceptors (Figures 7J and S7G). To establish if the difference observed was significant, the intensity of RHODOPSIN staining was measured as the percentage of pixels above threshold in the ONL-like layer, not including the segment region. RHODOPSIN staining of the ONL-like layer was significantly reduced in RK.RPGR-treated, compared with RK.GFP-treated organoids (Figures 7K; 7.1 ± 5.6% versus 22.1 ± 21.3% positive pixels, respectively; p = 0.006; two-tailed Mann-Whitney test; n = 30 images, N = 6 experiments). This confirms the improved RHODOPSIN localization observed in RPGR-deficient retinal organoids, following gene supplementation. These findings demonstrate the benefit of enriched culture conditions to investigate therapeutic interventions using hPSC-derived retinal organoids and support the use of this shortened RPGR construct for rescuing function in human as well as mouse photoreceptors.

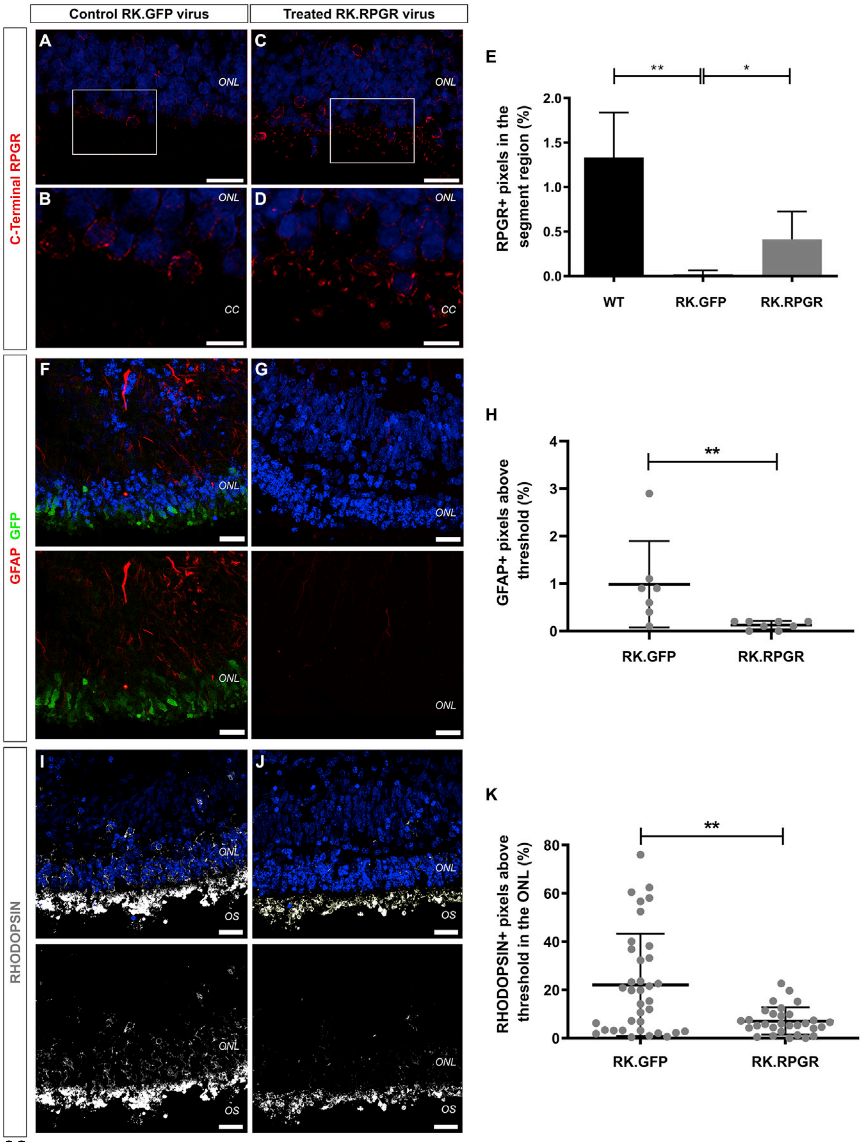

Figure 7. Characterization of RPGR disease phenotype following gene supplementation

(A–D) Representative images of iPSC-derived RPGR-deficient retinal organoids, transduced with either RK.GFP or RK.RPGR and stained for the RPGR-ORF15 isoform (red). High magnification images of insets in (A) and (C), highlighting the IS and CC region, are shown in (B) and (D), respectively.
(E) Histogram showing the percentage of positive pixels for RPGR-ORF15 staining in the segment region of untreated normal retinal organoids and treated RPGR-deficient retinal organoids (±SD; *p < 0.05, **p < 0.01; n ≥ 12 images, N = 4 experiments).
(F and G) Immunohistochemical analysis of GFAP$^+$ glial cell processes (red) in RPGR-deficient organoids, treated with RK.GFP or RK.RPGR virus. Transduced GFP$^+$ photoreceptors (green) can be seen in the RK.GFP-treated control (F).
(H) Graph showing the percentage of GFAP$^+$ pixels in treated RPGR-deficient organoids (±SD; **p < 0.01; n ≥ 7 images, N = 3 experiments).
(I and J) Immunohistochemical analysis demonstrating the localization of RHODOPSIN (gray) within rod photoreceptors, following either RK.GFP or RK.RPGR viral transduction.
(K) Graph showing the percentage of RHODOPSIN$^+$ pixels in the ONL region of treated RPGR-deficient retinal organoids (±SD; **p < 0.01; n = 30 images, N = 6 experiments). Nuclei were stained with DAPI (blue). Scale bars, 10 μm (B and D), 25 μm (A, C, F, G, I, and J). CC, connecting cilium; IS, inner segment; ONL, outer nuclear layer; OS, outer segment.

DISCUSSION

One limitation to the use of retinal organoids to model inherited retinal degenerations is that even after extensive long-term culture (>30weeks), hPSC-derived photoreceptors exhibit few nascent OS-like structures. Therefore, disease phenotypes related to the mature structure of photoreceptors, such as ciliopathies, have proved difficult to model effectively using retinal organoids alone. Here, we describe enhanced culture conditions that permit the generation of photoreceptors with well-developed OSs containing disc-like structures. These new conditions support the development of both rod and cone photoreceptor segments *in vitro*. Importantly, this protocol improves the proportion of photoreceptors that form OSs, providing a more robust model with which to investigate retinal disease. To demonstrate the utility of this modified protocol, we generated iPSC lines from patients with frameshift mutations in exon *ORF15* of the RPGR gene that result in XLRP3. RPGR-deficient retinal organoids demonstrated clear disease phenotypes including the mis-localization of RHODOPSIN, which was confined to the photoreceptor OSs in healthy control organoids but could also be found in the cell body and processes of mutant photoreceptors. This has been observed previously in KO mouse models but has not been reported using hPSC-derived photoreceptors to date, possibly because of the mis-localization of RHODOPSIN in control organoids, due to the inefficiency of OS formation (Megaw et al., 2017; Deng et al., 2018; Hong et al., 2005; Wu et al., 2015). Using AAV gene

supplementation to restore RPGR to the mutant photoreceptors, we demonstrated a significant reduction in RHODOPSIN mis-localization, ameliorating the phenotype despite incomplete transduction efficiency. We have previously shown that gene therapy using a shortened RPGR-ORF15 transgene improves photoreceptor function and viability in an animal model of RPGR deficiency (Pawlyk et al., 2016). Here we demonstrate that the shortened transgene is also able to restore function in human photoreceptor cells, providing additional validation of a construct that is currently being used in clinical trials of gene therapy for XLRP3 (ClinicalTrials.gov: NCT03252847).

LC-PUFAs are known to be essential for both brain and retinal development, with dietary restriction resulting in reduced visual function (Wheeler et al., 1975; Neuringer et al., 1984). DHA, a major component of retinal phospholipids, is actively sequestered via the choroidal blood flow and influences rhodopsin content at the disk membranes, as well as photoresponses and disk morphogenesis (Shindou et al., 2017; Anderson et al., 1992; Nguyen et al., 2014). Previous studies have examined the effects of DHA on the differentiation and survival of photoreceptors, from primary retinal progenitors and, more recently, derived from both mouse and human PSCs (Arai et al., 2017; Rotstein et al., 1997; Brooks et al., 2019). Despite increased levels of rhodopsin expression and improved inner segment and CC formation, little improvement in brush border density or OS ultrastructure were demonstrated (Arai et al., 2017; Brooks et al., 2019). In this study, we determined that increased antioxidant levels were required to enable the beneficial effects of DHA to be observed. Therefore, the higher concentrations of BSA-bound lipids used here most likely account for the difference in findings (Arai et al., 2017; Brooks et al., 2019). The exact proportions of specific PUFAs present in lipid-rich BSA (AlbuMAX) are undefined. However, we demonstrate, for the first time to our knowledge, mESC-derived photoreceptors exhibiting membranous OS-like structures, following the addition of either BSA-bound DHA or lipid-rich BSA. Interestingly, the inclusion of BSA-bound lipids also improved the segment structures and M/L opsin content of hPSC-derived cone photoreceptors. Unlike rods, cone OSs are formed by a continuation of the plasma membrane and are comprised of lower levels of DHA or omega-3 relative to omega-6 PUFAs, suggesting a different biophysical lipid requirement (Young 1969; Agbaga et al., 2018). Metabolic cross-talk between the two photoreceptor subtypes, mediated by rod-derived cone viability factor, means we cannot exclude the possibility that improved cone OS formation is a secondary effect of enhanced rod photoreceptor maturation (Aït-Ali et al., 2015). It is also important to note that while our protocol resulted in the efficient generation of hPSC-derived photoreceptors exhibiting organized OSs, the formation and maintenance of perfectly stacked discs in all photoreceptors has yet to be achieved. This will most likely require additional support at the level of the photoreceptor segments, such as that provided by the close apposition of RPE cells *in vivo*. While little difference in gross OS morphology was observed for DHA supplemented retinal organoids, it remains to be determined if there is a functional difference between hPSC-derived photoreceptors grown with increased concentrations of DHA, as opposed to other LC-PUFAs. Despite this, we have continued to use our enhanced media in combination with DHA supplementation for retinal disease modeling. In addition, it will be of interest to establish if the improved OS ultrastructure observed here results in enhanced electrophysiological responses to light. Further studies are needed to address these limitations and determine if retinal organoids maintained with BSA-bound lipids can provide a light-responsive model system for the human retina.

In summary, we designed a nutrient-rich medium to support the increased energetic and biosynthetic demands of maturing PSC-derived photoreceptors. In contrast to our original culture conditions, in which only a limited number of hPSC-derived photoreceptors exhibited rudimentary OS-like structures, these new conditions permitted the efficient development of OS-bearing rod and cone photoreceptors. These improvements were not limited to the increased frequency of OS development in hPSC-derived photoreceptors, but also resulted in better organization, with membranous structures reminiscent of stacked OS discs. This enabled us to effectively model cellular defects in XLRP and demonstrate rescue by gene supplementation. Together, these findings suggest that our enhanced culture protocol facilitates the development of more structurally mature hPSC-derived photoreceptors, and this may be useful for inherited retinal disease modeling and *in vitro* testing of novel therapeutic strategies.

EXPERIMENTAL PROCEDURES

See supplemental experimental procedures for detailed protocols.

Mouse ESC culture and retinal differentiation

Mouse ESCs were maintained and differentiated to form EBs containing retinal regions as previously described (Kruczek et al., 2017). From day 21 onward the media used was either standard RMM, AOX, or ALT media (see Table S1), supplemented with 50 μM DHA, 12.5 μM fatty acid-free BSA, additional glucose (25 mM final concentration), or AlbuMAX II (0.4 mg/ml).

Human PSC culture and retinal differentiation

hPSCs (see Table S2) were maintained and differentiated as previously described to generate NRVs (Gonzalez Cordero et al., 2017). From 12 weeks of differentiation, media were changed to

either standard RDM90 or ALT medium and supplemented with 50 μM DHA or 12.5 μM fatty acid-free BSA.

Production and use of recombinant AAV viral vector

Both pD10/RK*promoter-GFP* and pD10/RK*promoter-RPGR* constructs containing AAV-2 inverted terminal repeats were used to generate AAV7m8.RK.GFP and AAV7m8.RK.RPGR viral vector. Retinal organoids were infected at 15–18weeks with 3×10^{11} viral particles per organoid, with an estimated gMOI of 6×10^5.

Immunohistochemical analysis

See supplemental experimental procedures (Table S3) for full details. Images were acquired with a confocal microscopy (Leica DM5500Q) and LAS AF image software. Image analysis was performed using FiJi and Gimp 2.8.22 software and blinded, wherever possible.

Ultrastructural analysis

For TEM, sections were imaged with a JEOL 11010 TEM operating at 80 V and acquired with a Gatan Orius camera using Digital Micrograph software. For scanning electron microscopy, specimens were imaged in a Zeiss Sigma FESEM operating at 3–5 kV. For 3view, stacks of backscatter electron micrographs were automatically acquired using a Gatan 3view system working in conjunction with a Zeiss Sigma field emission scanning electron microscope. Stacks were converted to TIFF images in Digital Micrograph software, prior to importation into Amira 5.3.3 software.

Statistical analysis

In all experiments, means are presented ±SD, unless otherwise stated; n = number of images, sections, EBs, or organoids examined; N = number of independent differentiations, cell lines, or experiments performed. Graphpad Prism 6 software was used for statistical analysis.

SUPPLEMENTAL INFORMATION

Supplemental information can be found online at https://doi.org/10.1016/j.stemcr.2022.02.019.

AUTHOR CONTRIBUTIONS

Conceptualization, E.L.W., A.G-C., J.W.B.B., and R.R.A.; Methodology, E.L.W., A.G-C., P.M., M.F., A.N., P.O-R., R.S., M.H., and A.G.; Investigation, E.L.W., A.G-C., P.M., A.N., M.F., M.O'H-W., E.L., R.S., J.R., N.J., and I.O.S.; Resources, M.H. and J.W.B.B.; Writing – Original Draft, E.L.W. and A.G-C.; Writing – Review & Editing, A.G., A.J.S., and R.R.A.; Supervision, A.J.S., J.W.B.B., and R.R.A.; Funding Acquisition, J.W.B.B. and R.R.A.

CONFLICTS OF INTEREST

The authors declare no competing interests.

ACKNOWLEDGMENTS

We thank R. Maswood, O. Semenyuk, and I. Mahamoud from the Institute of Ophthalmology (IoO) vector production facility and D. Sefic-Svara from the IoO microscopy unit for their technical support. The graphical abstract was generated using BioRender (https://biorender.com). This work was supported by grants from the Medical Research Council UK (MR/J004553/1; MR/M007871/1; MR/L012758/1) and European Research Council (ERC-2012-ADG_20120314)). Fight For Sight (1448/1449), the Macular Vision Research Foundation, The Miller's Trust, Moorfields Eye Charity, and a generous donation by Mr Otto van der Wyck.

Received: August 23, 2021
Revised: February 26, 2022
Accepted: February 28, 2022
Published: March 24, 2022

REFERENCES

Anderson, R.E., O'Brien, P.J., Wiegand, R.D., Koutz, C.A., and Stinson, A.M. (1992). Conservation of docosahexaenoic acid in the retina. Adv. Exp. Med. Biol. *318*, 285–294.

Agbaga, M.-P., Merriman, D.K., Brush, R.S., Lydic, T.A., Conley, S.M., Naash, M.I., Jackson, S., Woods, A.S., Reid, G.E., Busik, J.V., et al. (2018). Differential composition of DHA and very-long-chain PUFAs in rod and cone photoreceptors. J. Lipid Res. *59*, 1586–1596.

Aït-Ali, N., Fridlich, R., Millet-Puel, G., Clérin, E., Delalande, F., Jaillard, C., Blond, F., Perrocheau, L., Reichman, S., Byrne, L.C., et al. (2015). Rod-derived cone viability factor promotes cone survival by stimulating aerobic glycolysis. Cell *161*, 817–832.

Arai, E., Parmar, V.M., Sahu, B., Perusek, L., Parmar, T., and Maeda, A. (2017). Docosahexaenoic acid promotes differentiation of photoreceptor cells in three-dimensional neural retinas. Neurosci. Res. *123*, 1–7.

Bazan, N.G., Gordon, W.C., and de Turco, E.B.R. (1992). Docosahexaenoic acid uptake and metabolism in photoreceptors: retinal conservation by an efficient retinal pigment epithelial cell-mediated recycling process. Adv. Exp. Med. Biol. *318*, 295–306.

Boulton, M., and Dayhaw-Barker, P. (2001). The role of the retinal pigment epithelium: topographical variation and ageing changes. Eye (London, England) *15*, 384–389.

Brooks, M.J., Chen, H.Y., Kelley, R.A., Mondal, A.K., Nagashima, K., Val, N.D., Li, T., Chaitankar, V., and Swaroop, A. (2019). Improved retinal organoid differentiation by modulating signaling pathways revealed by comparative transcriptome analyses with development in vivo. Stem Cell Rep. *13*, 891–905.

Deng, W.-L., Gao, M.-L., Lei, X.-L., Lv, J.-N., Zhao, H., He, K.-W., Xia, X.-X., Li, L.-Y., Chen, Y.-C., Li, Y.-P., et al. (2018). Gene correction reverses ciliopathy and photoreceptor loss in iPSC-derived retinal organoids from retinitis pigmentosa patients. Stem Cell Rep. *10*, 1267–1281.

Gakovic, M., Shu, X., Kasioulis, I., Carpanini, S., Moraga, I., and Wright, A.F. (2011). The role of RPGR in cilia formation and actin stability. Hum. Mol. Genet. *20*, 4840–4850.

Gao, M.-L., Lei, X.-L., Han, F., He, K.-W., Jin, S.-Q., Zhang, Y.-Y., and Jin, Z.-B. (2020). Patient-specific retinal organoids recapitulate disease features of late-onset retinitis pigmentosa. Front. Cell Dev. Biol. *8*, 128.

Gonzalez-Cordero, A., West, E.L., Pearson, R.A., Duran, Y., Carvalho, L.S., Chu, C.J., Naeem, A., Blackford, S.J.I., Georgiadis, A.,

Lakowski, J., et al. (2013). Photoreceptor precursors derived from three-dimensional embryonic stem cell cultures integrate and mature within adult degenerate retina. Nat. Biotechnol. *31*, 741–747.

Gonzalez-Cordero, A., Kruczek, K., Naeem, A., Fernando, M., Kloc, M., Ribeiro, J., Goh, D., Duran, Y., Blackford, S.J.I., Abelleira-Hervas, L., et al. (2017). Recapitulation of human retinal development from human pluripotent stem cells generates transplantable populations of cone photoreceptors. Stem Cell Rep. *9*, 820–837.

Hong, D.H., Yue, G., Adamian, M., and Li, T. (2001). Retinitis pigmentosa GTPase regulator (RPGRr)-interacting protein is stably associated with the photoreceptor ciliary axoneme and anchors RPGR to the connecting cilium. J. Biol. Chem. *276*, 12091–12099.

Hong, D.-H., Pawlyk, B.S., Adamian, M., Sandberg, M.A., and Li, T. (2005). A single, abbreviated RPGR-ORF15 variant reconstitutes RPGR function in vivo. Invest. Ophthalmol. Vis. Sci. *46*, 435–441.

Khani, S.C., Pawlyk, B.S., Bulgakov, O.V., Kasperek, E., Young, J.E., Adamian, M., Sun, X., Smith, A.J., Ali, R.R., and Li, T. (2007). AAV-mediated expression targeting of rod and cone photoreceptors with a human rhodopsin Kinase promoter. Invest. Ophth Vis. Sci. *48*, 3954–3961.

Kruczek, K., Cordero, A.G., Goh, D., Naeem, A., Jonikas, M., Blackford, S.J.I., Kloc, M., Duran, Y., Georgiadis, A., Sampson, R.D., et al. (2017). Differentiation and transplantation of embryonic stem cell-derived cone photoreceptors into a mouse model of end-stage retinal degeneration. Stem Cell Rep. *8*, 1659–1674.

Lane, A., Jovanovic, K., Shortall, C., Ottaviani, D., Panes, A.B., Schwarz, N., Guarascio, R., Hayes, M.J., Palfi, A., Chadderton, N., et al. (2020). Modeling and rescue of RP2 retinitis pigmentosa using iPSC-derived retinal organoids. Stem Cell Rep. *15*, 67–79.

Lukovic, D., Castro, A.A., Kaya, K.D., Munezero, D., Gieser, L., Davó-Martínez, C., Corton, M., Cuenca, N., Swaroop, A., Ramamurthy, V., et al. (2020). Retinal organoids derived from hiPSCs of an AIPL1-LCA patient maintain cytoarchitecture despite reduced levels of mutant AIPL1. Sci. Rep. *10*, 5426.

Megaw, R., Abu-Arafeh, H., Jungnickel, M., Mellough, C., Gurniak, C., Witke, W., Zhang, W., Khanna, H., Mill, P., Dhillon, B., et al. (2017). Gelsolin dysfunction causes photoreceptor loss in induced pluripotent cell and animal retinitis pigmentosa models. Nat. Commun. *8*, 271.

Neuringer, M., Connor, W.E., Petten, C.V., and Barstad, L. (1984). Dietary omega-3 fatty acid deficiency and visual loss in infant rhesus monkeys. J. Clin. Invest. *73*, 272–276.

Nguyen, L.N., Ma, D., Shui, G., Wong, P., Cazenave-Gassiot, A., Zhang, X., Wenk, M.R., Goh, E.L.K., and Silver, D.L. (2014). Mfsd2a is a transporter for the essential omega-3 fatty acid docosahexaenoic acid. Nature *509*, 503–506.

O'Hara-Wright, M., and Cordero, A.G. (2020). Retinal organoids: a window into human retinal development. Development *147*, dev189746.

Ovando-Roche, P., West, E.L., Branch, M.J., Sampson, R.D., Fernando, M., Munro, P., Georgiadis, A., Rizzi, M., Kloc, M., Naeem, A., et al. (2018). Use of bioreactors for culturing human retinal organoids improves photoreceptor yields. Stem Cell Res. Ther., 1–14.

Parfitt, D.A., Lane, A., Ramsden, C.M., Carr, A.-J.F., Munro, P.M., Jovanovic, K., Schwarz, N., Kanuga, N., Muthiah, M.N., Hull, S., et al. (2016). Identification and correction of mechanisms underlying inherited blindness in human iPSC-derived optic cups. Cell Stem Cell *18*, 769–781.

Pawlyk, B.S., Bulgakov, O.V., Sun, X., Adamian, M., Shu, X., Smith, A.J., Berson, E.L., Ali, R.R., Khani, S., Wright, A.F., et al. (2016). Photoreceptor rescue by an abbreviated human RPGR gene in a murine model of X-linked retinitis pigmentosa. Gene Ther. *23*, 196–204.

Rice, D.S., Calandria, J.M., Gordon, W.C., Jun, B., Zhou, Y., Gelfman, C.M., Li, S., Jin, M., Knott, E.J., Chang, B., et al. (2015). Adiponectin receptor 1 conserves docosahexaenoic acid and promotes photoreceptor cell survival. Nat. Commun. *6*, 6228.

Rotstein, N.P., Aveldaño, M.I., Barrantes, F.J., Roccamo, A.M., and Politi, L.E. (1997). Apoptosis of retinal photoreceptors during development in vitro: protective effect of docosahexaenoic acid. J. Neurochem. *69*, 504–513.

Shindou, H., Koso, H., Sasaki, J., Nakanishi, H., Sagara, H., Nakagawa, K.M., Takahashi, Y., Hishikawa, D., Iizuka-Hishikawa, Y., Tokumasu, F., et al. (2017). Docosahexaenoic acid preserves visual function by maintaining correct disc morphology in retinal photoreceptor cells. J. Biol. Chem. *292*, 12054–12064.

Strauss, O. (2005). The retinal pigment epithelium in visual function. Physiol. Rev. *85*, 845–881.

Tee, J.J.L., Smith, A.J., Hardcastle, A.J., and Michaelides, M. (2016). RPGR-associated retinopathy: clinical features, molecular genetics, animal models and therapeutic options. Br. J. Ophthalmol. *100*, 1022–1027.

Wahlin, K.J., Maruotti, J.A., Sripathi, S.R., Ball, J., Angueyra, J.M., Kim, C., Grebe, R., Li, W., Jones, B.W., and Zack, D.J. (2017). Photoreceptor outer segment-like structures in long-term 3D retinas from human pluripotent stem cells. Sci. Rep. *7*, 766.

Wheeler, T.G., Benolken, R.M., and Anderson, R.E. (1975). Visual membranes: specificity of fatty acid precursors for the electrical response to illumination. Science (New York, N.Y.) *188*, 1312–1314.

Wu, Z., Hiriyanna, S., Qian, H., Mookherjee, S., Campos, M.M., Gao, C., Fariss, R., Sieving, P.A., Li, T., Colosi, P., et al. (2015). A long-term efficacy study of gene replacement therapy for RPGR-associated retinal degeneration. Hum. Mol. Genet. *24*, 3956–3970.

Young, R.W. (1969). A difference between rods and cones in the renewal of outer segment protein. Invest. Ophthalmol. *8*, 222–231.

Zhong, X., Gutierrez, C., Xue, T., Hampton, C., Vergara, M.N., Cao, L.-H., Peters, A., Park, T.S., Zambidis, E.T., Meyer, J.S., et al. (2014). Generation of three-dimensional retinal tissue with functional photoreceptors from human iPSCs. Nat. Commun. *5*, 4047.

Zhou, S., Flamier, A., Abdouh, M., Tétreault, N., Barabino, A., Wadhwa, S., and Bernier, G. (2015). Differentiation of human embryonic stem cells into cone photoreceptors through simultaneous inhibition of BMP, TGFβ and Wnt signaling. Development *142*, 3294–3306.

Stem Cell Reports, Volume *17*

Supplemental Information

Antioxidant and lipid supplementation improve the development of photoreceptor outer segments in pluripotent stem cell-derived retinal organoids

Emma L. West, Paromita Majumder, Arifa Naeem, Milan Fernando, Michelle O'Hara-Wright, Emily Lanning, Magdalena Kloc, Joana Ribeiro, Patrick Ovando-Roche, Ian O. Shum, Neeraj Jumbu, Robert Sampson, Matt Hayes, James W.B. Bainbridge, Anastasios Georgiadis, Alexander J. Smith, Anai Gonzalez-Cordero, and Robin R. Ali

Supplemental Information

Supplemental Figures

Figure S1. Long term survival and organisation of mESC-derived photoreceptors in retinal cultures, *related to Figure 1*

(**A**-**H**) Immunohistochemical images demonstrating examples of embryoid body sections for the relevant categories of organisation, non-retinal cells (**A**,**E**), organised PRs (**B**,**C**,**F**,**G**) and disorganised PRs (**D**,**H**). DAPI (blue) was used to determine the presence of ONL-like layers (dashed white lines) within retinal regions, that contained organised Recoverin+ photoreceptors (red; white arrow heads). Crx.GFP^{+} photoreceptors (green) corresponded to the Recoverin^{+} regions observed. (**I**) Flow cytometry plots showing the gating strategy used to determine the percentage of Crx.GFP^{+}/CD73^{+} rod photoreceptors present in dissociated retinal cultures with time. Control samples included an unstained CCE cell sample (**Unstained cells**), unstained Crx.GFP cell sample (**Crx.GFP cells**), CD73 stained CCE cell sample (**CD73 stained cells**) and CD73 stained Crx.GFP cell sample (**Stained Crx.GFP cells**).
Scale bars: 200 μm (**A-D**) and 50 μm (**E-H**). Abbreviations: PRs, photoreceptors.

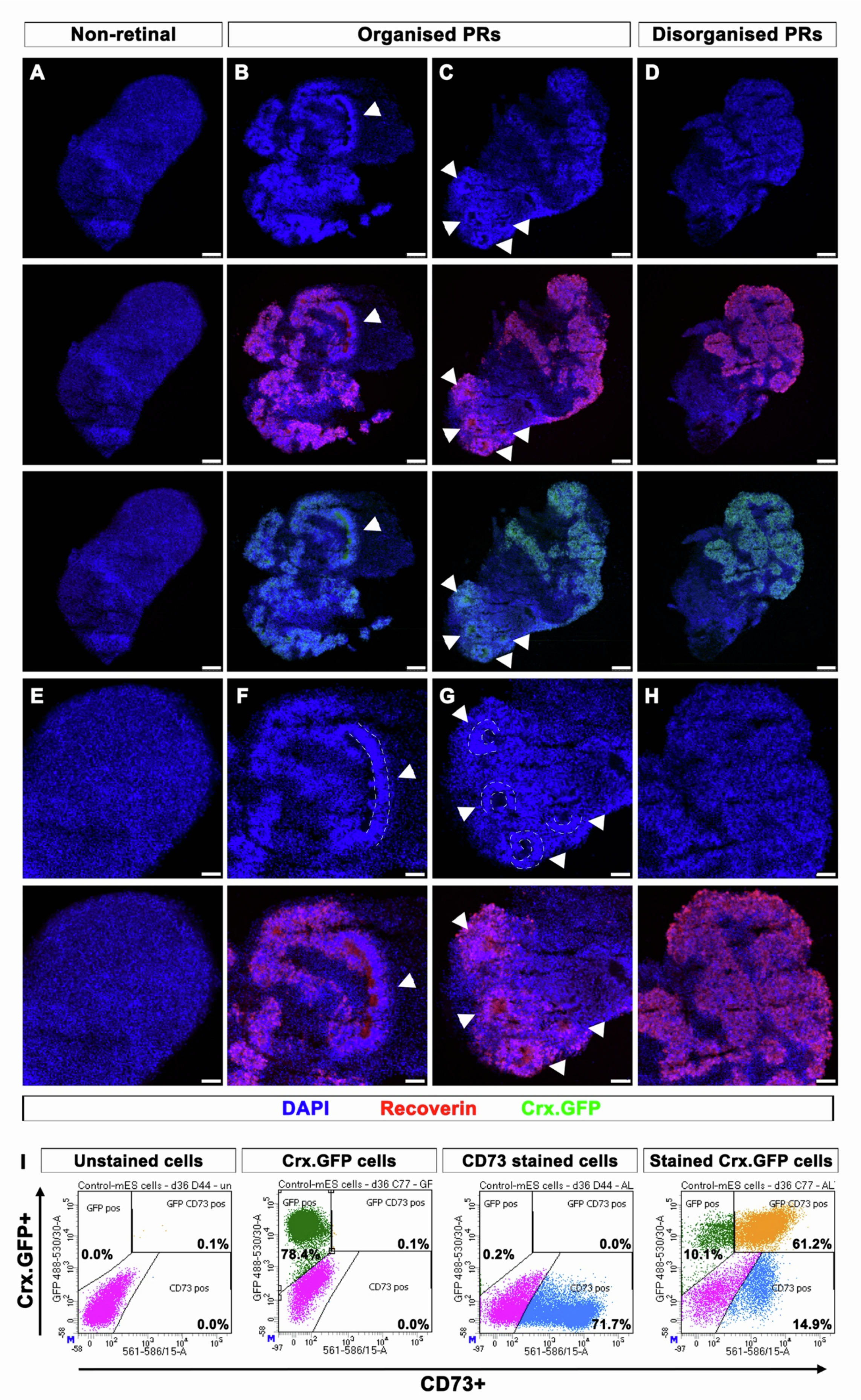
Non-retinal
Organised PRs
Disorganised PRs
A
B
C
D
E
F
G
H
DAPI
Recoverin
Crx.GFP
I
Unstained cells
Crx.GFP cells
CD73 stained cells
Stained Crx.GFP cells
Control-mES cells - d36 D44 - un
Control-mES cells - d36 C77 - GF
Control-mES cells - d36 D44 - AL
GFP pos
GFP CD73 pos
CD73 pos
0.0%
0.1%
0.0%
0.0%
78.4%
0.1%
0.0%
0.2%
0.0%
71.7%
10.1%
61.2%
14.9%
GFP 488-530/30-A
561-586/15-A
Crx.GFP+
CD73+

Figure S2. Characterisation of photoreceptor morphology and organisation in various culture conditions using different mESC lines, *related to Figure 1*

(**A**-**F**) Representative confocal images of mESC-derived retinal neuroepithelia (Crx.GFP line, green photoreceptors) at day 34, maintained in 3 different culture media with +BSA or +DHA and stained for Rhodopsin (grey) and Peripherin2 (red). (**G**-**L**) Representative confocal images of mESC-derived retinal neuroepithelia (CCE cell line) at day 34, maintained in 3 different culture media with +BSA or +DHA and stained for Rhodopsin (grey) and Peripherin2 (red). (**M**) Histogram showing the percentage of sections as classified into 3 set categories (non-retinal, organised and disorganised PRs) maintained in AOX culture media with +BSA, additional glucose (+Glucose), lipid-rich BSA (+AlbuMAX) and +DHA (error bars, mean ± SEM, $p > 0.05$; n>12 sections, N>3 independent experiments). (**N**-**Q**) Representative confocal images of mESC-derived retinal regions (Crx.GFP line; green photoreceptors) at day 34, maintained in AOX culture media with with +BSA (**N**), additional glucose (**O**; +Glucose), lipid-rich BSA (**P**; +AlbuMAX) and +DHA (**Q**) and stained for Rhodopsin (grey) and Peripherin2 (red).
Scale bars: 10 μm (**A**-**F**, **N-Q**) and 25 μm (**G**-**L**). Abbreviations: PRs, photoreceptors.

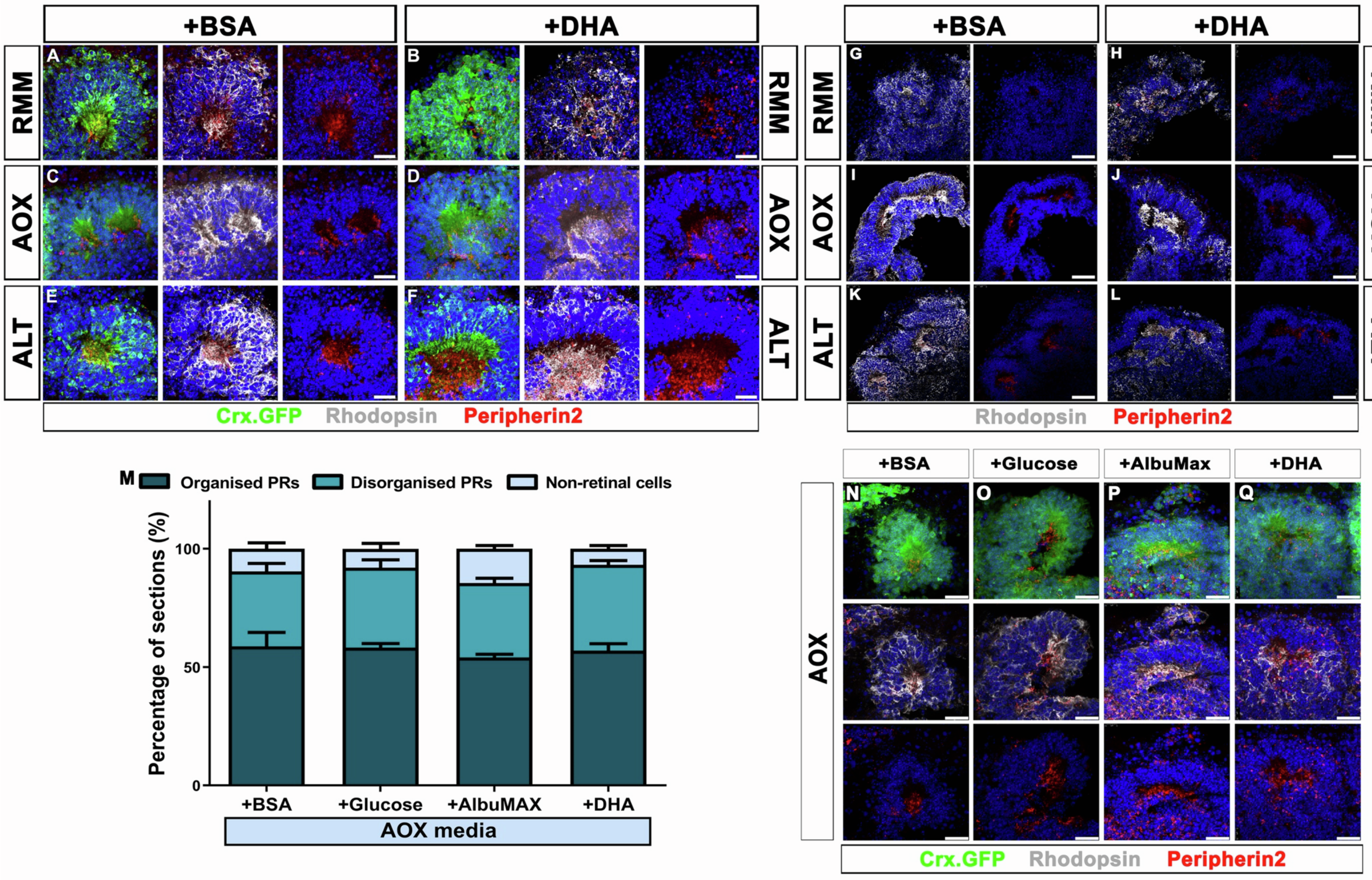
+BSA
+DHA
RMM
AOX
ALT
A
B
C
D
E
F
Crx.GFP
Rhodopsin
Peripherin2
G
H
I
J
K
L
M
Organised PRs
Disorganised PRs
Non-retinal cells
Percentage of sections (%)
100
50
0
+BSA
+Glucose
+AlbuMAX
+DHA
AOX media
+AlbuMax
N
O
P
Q

Figure S3. Characterisation of segment morphology in DHA and ALT supplemented retinal organoid cultures, *related to Figure 2 & 3*

(**A**) Representative bright field image of wk 26 retinal organoid. (**B**) High magnification image of neuroepithelia showing brush border regions in standard RDM90+BSA media. (**C**) IHC analysis showing MITOCHONDRIA rich ISs and developing PRPH2+ OSs. (**D, E**) Representative bright field image of wk 26 retinal organoid and neuroepithelia (**E**, high magnification) showing no differences in brush border developed in RDM90+DHA media. (**F**) IHC analysis showing MITOCHONDRIA rich ISs and developing PRPH2+ OSs. (**G**) Image of RDM90+BSA cultured retinal organoid showing ESPIN positive structures in the CC region and ABCA4 positive OSs. (**H**) Image of ALT+BSA cultured retinal organoid showing ESPIN positive structures in the CC region and elongated ABCA4 positive OSs.
(**I-P**) Representative bright field images of retinal organoids generated from various pluripotent stem cell lines, cultured in either RDM90+BSA (**I**, **K**, **M**, **O**) or ALT+BSA (**J**, **L**, **N**, **P**).
Scale bars: 25μm (**C, F**, **G**, **H**), 50μm (**A-E**, **I-P**). Abbreviations: CC, connecting cilia; IS, inner segment; OS, outer segment; ONL, outer nuclear layer.

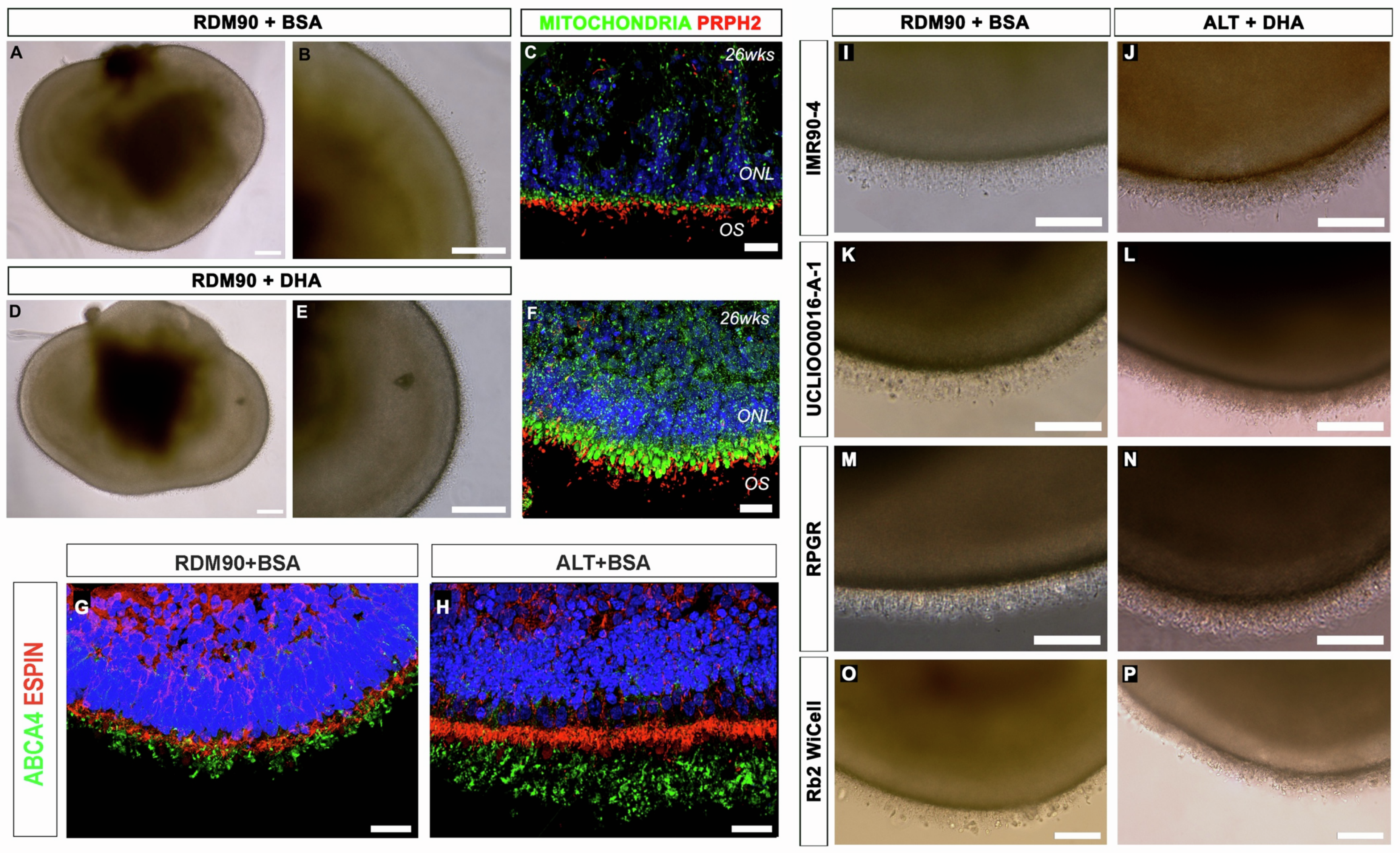
RDM90 + BSA
A
B
MITOCHONDRIA PRPH2
C
26wks
ONL
OS
RDM90 + DHA
D
E
F
26wks
ONL
OS
RDM90+BSA
ALT+BSA
ABCA4 ESPIN
G
H
RDM90 + BSA
ALT + DHA
IMR90-4
I
J
UCLIOO0016-A-1
K
L
RPGR
M
N
Rb2 WiCell
O
P

Figure S4. Ultrastructure analysis of the segment region of retinal organoids in various conditions, *related to Figure 5*

(**A**-**C**) Electron micrographs showing RDM90+BSA grown photoreceptor cells. Insets in **B** highlight two OSs that are shown in higher magnification in panels above.
(**D-F**) Images showing OSs of ALT+BSA cultures. Inset is shown in higher magnification in the panel above**.** OS disk membranes were more discernable in these cultures (**D**-**F**). (**G-K**) Electron micrographs of an ALT+DHA grown retinal organoid showing the brush border region. The ultrastructure of this region of the retinal neuroepithelia demonstrated numerous OSs (**G**). Dashed line inset in **G** is shown in high magnification in **H** and **I** to highlight OS morphology, with clearly visible stacked OS disk membranes. Solid line inset in **G** is shown in higher magnification in **K** and **J** to highlight another region with a number of OSs, with clearly visible stacked OS disk membranes.
Scale bars: 1μm (**E**), 2μm (**B**), 5μm (**A**, **D**, **I**, **J**), 10μm (**C**, **G**, **H**, **K**). Abbreviations: OS, outer segment; CC, connecting cilia; IS, inner segment.

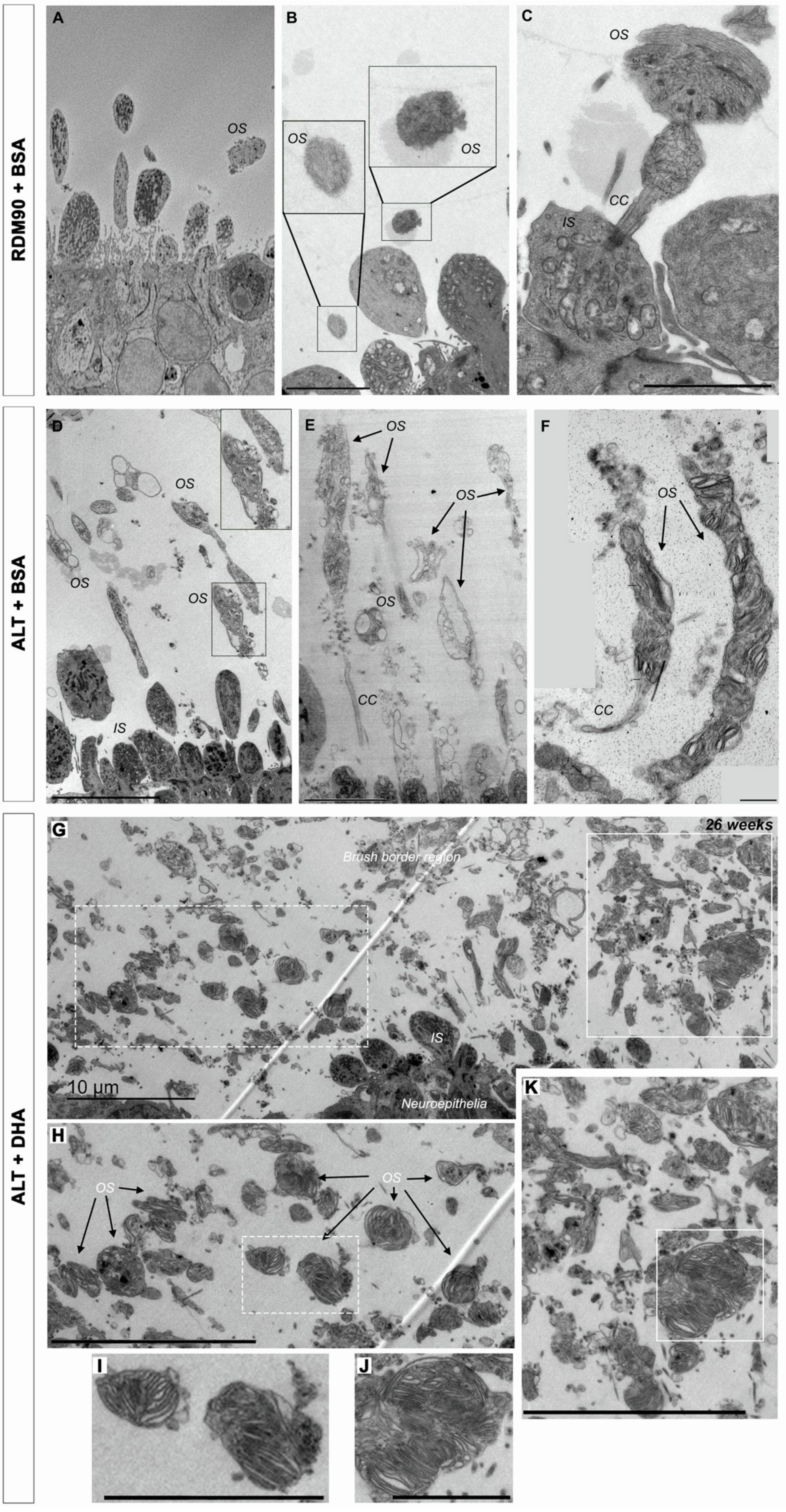
RDM90 + BSA
ALT + BSA
ALT + DHA
A
B
C
D
E
F
G
H
I
J
K
OS
CC
IS
26 weeks
Brush border region
Neuroepithelia
10 µm

Figure S5. 3D Ultrastructural analysis of photoreceptor segment structures present in RDM90 and ALT cultured retinal organoids, *related to Figure 5*

(**A-D**) 3view sequence of backscatter EM images of hPSC-derived retinal neuroepithelia showing photoreceptor OS (magenta), CC (blue) and IS (green). (**A**, **C**) 3view 3D reconstruction of 150 sections with thickness of 100 nm each. (**B**, **D**) Single 3view section of 100nm. (**E**, **J**) Representative bright field images of retinal organoids cultured in RDM90+BSA or ALT+BSA, showing brush border structures apical to neuroepithelia regions. (**F**, **K**) SEM micrographs showing topography of whole retinal organoids highlighting the neuroepithelia region. (**G-I**, **L-N**) Topographic features of neuroepithelia showing photoreceptor cell density and morphology from RDM90+BSA (**G-I**) or ALT+BSA (**L-N**) cultured organoids, at ascending magnifications.

Scale bars: 2μm (**M, N**), 3μm (**H**, **I**), 10μm (**G**, **L**), 20μm (**F**, **K**), 50μm (**E**, **J**). Abbreviations: CC, connecting cilium; IS, inner segment; OLM, outer limiting membrane; ONL, outer nuclear layer; OS, outer segment; RPE, retinal pigment epithelium.

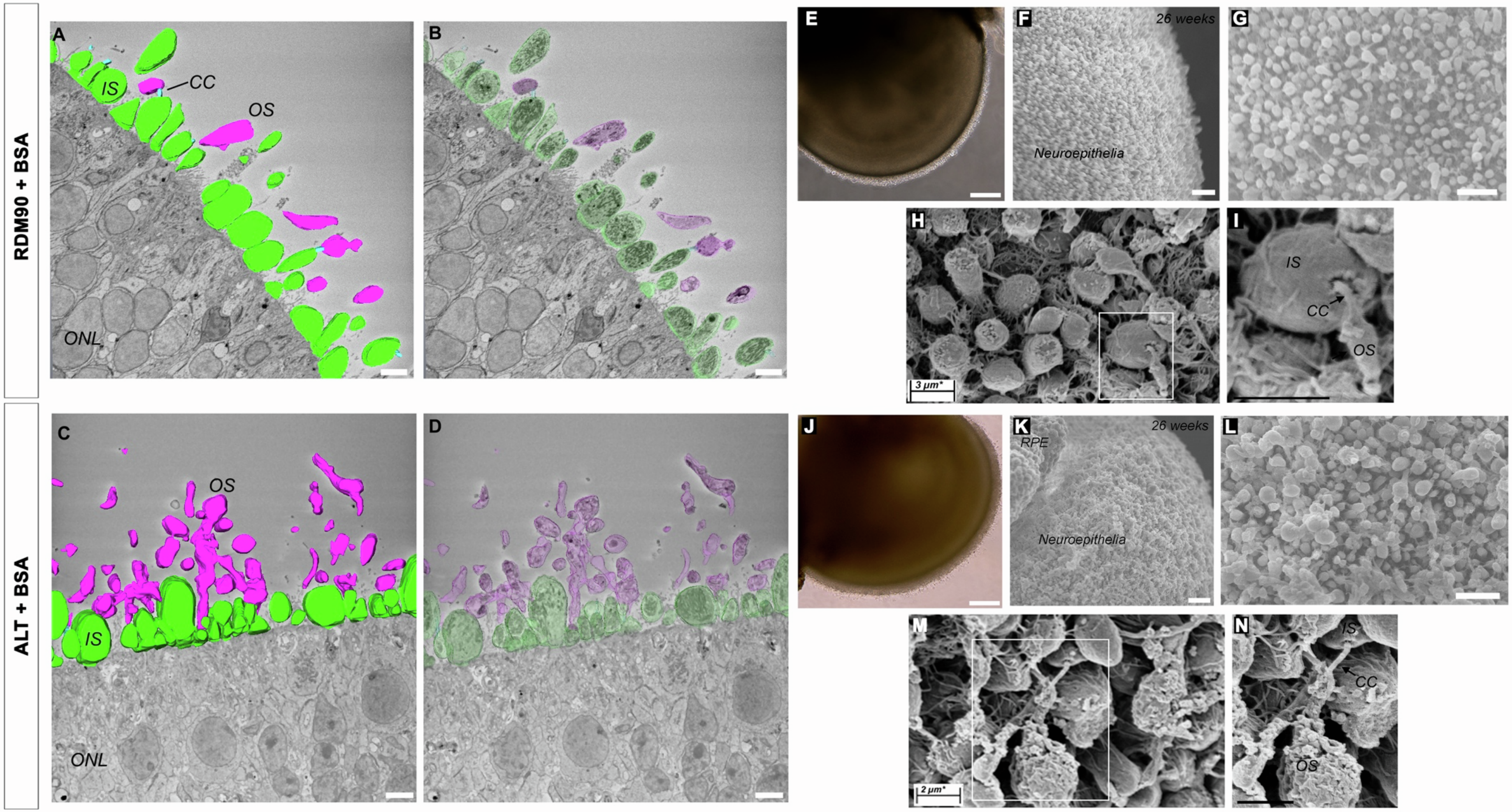
RDM90 + BSA
ALT + BSA
A
IS
CC
OS
ONL
B
C
OS
IS
ONL
D
E
F
26 weeks
Neuroepithelia
G
H
3 µm*
I
IS
CC
OS
J
K
RPE
26 weeks
Neuroepithelia
L
M
2 µm*
N
IS
CC
OS

Figure S6. RPGR iPSC line characterisation, *related to Figure 6*

(**A-R**) Typical iPSC colonies appearance and morphology for all 3 RPGR patient iPSC lines generated (**A, G, M**). All iPSC lines showed a normal male karyotype (**B, H, N**). Immunocytochemistry of iPSCs demonstrated the presence of pluripotency markers SOX2, OCT3/4, C-MYC and NANOG for all lines (**C-F, I-L, O-R**). (**S-U**) Sequencing confirmed the presence of mutations within the RPGR *ORF15* gene for all lines. (**V**) The TaqMan hPSC Scorecard assay was performed on all hPSC lines used in this study. Box and whisker plot confirmed that RPGR1-3 iPSC lines average expression of self-renewal and embryonic germ layer genes fall within the expected range in terms of self-renewal, ectoderm and mesoderm gene expression and are similar to the 3 control lines (H9, Rb2 and IMR90-4) used in this study. (**W**) Representative bright field images of iPSC-derived retinal organoids from all 3 RPGR patient lines, at 7-9 weeks of development.
Scale bars: 75µm (**C-F, I-L, O-R**), 100µm (**W**).

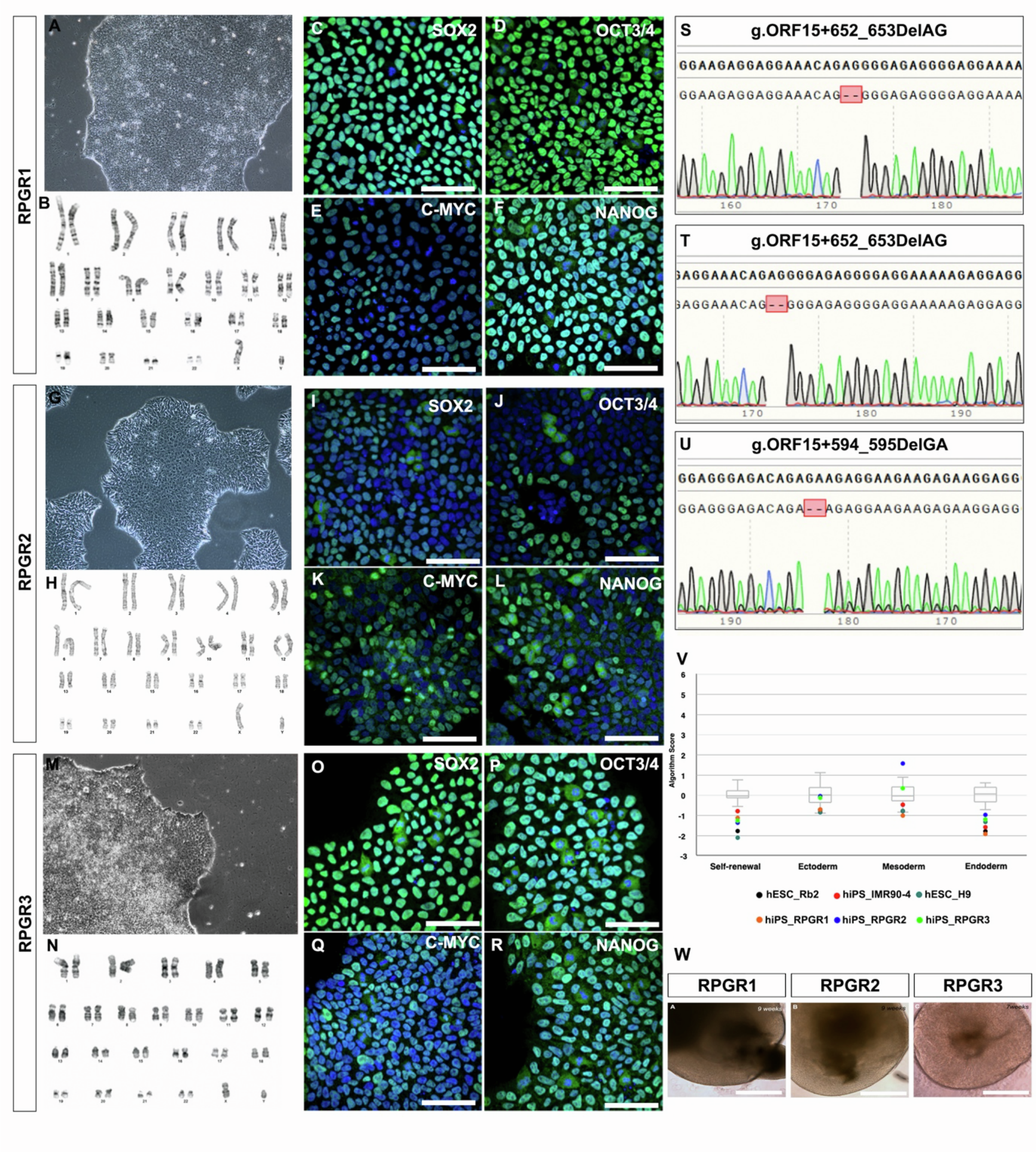

RPGR1
RPGR2
RPGR3
A
B
C
SOX2
D
OCT3/4
E
C-MYC
F
NANOG
G
H
I
SOX2
J
OCT3/4
K
C-MYC
L
NANOG
M
N
O
SOX2
P
OCT3/4
Q
C-MYC
R
NANOG
S
g.ORF15+652_653DelAG
GGAAGAGGAGGAAACAGAGGGGAGAGGGGAGGAAAA
GGAAGAGGAGGAAACAG--GGGAGAGGGGAGGAAAA
160
170
180
T
g.ORF15+652_653DelAG
170
180
190
U
g.ORF15+594_595DelGA
GGAGGGAGACAGAGAAGAGGAAGAAGAGAAGGAGG
GGAGGGAGACAGA--AGAGGAAGAAGAGAAGGAGG
190
180
170
V
Algorithm Score
Self-renewal
Ectoderm
Mesoderm
Endoderm
hESC_Rb2
hiPS_IMR90-4
hESC_H9
hiPS_RPGR1
hiPS_RPGR2
hiPS_RPGR3
W
RPGR1
RPGR2
RPGR3

Figure S7. Characterisation of iPSC-derived RPGR-deficient retinal organoids, with and without AAV-mediated gene supplementation, *related to Figure 6 & 7*

(**A**) IHC analysis showing no differences in Phalloidin (magenta) expression in the OLM of control (IMR90-4) and RPGR-deficient organoids. (**B**) IHC showing upregulation of GFAP (green) in RPGR-deficient compared to control retinal neuroepithelia. (**C**) IHC showing co-localisation of CRALBP (grey) and GFAP (green) in Müller glial cells. (**D**) IHC analysis for RHODOPSIN (red) and Phalloidin (grey) in control and RPGR-deficient retinal organoids showing mis-localisation of RHODOPSIN to the ONL region. Insets are shown at a higher magnification in the top right panel, without DAPI (blue), for these regions. (**E**) Schematic showing the experimental design for the treatment of iPSC-derived retinal organoids with viral vectors. Representative image of an RPGR-deficient retinal organoid transduced with AAV 7m8.RK.GFP, at 33 wks in culture. The transduction efficiency of AAV 7m8.RK.GFP, shown by the percentage of GFP+ cells in the ONL-like layer of retinal organoid sections, was 44 ± 11.4% (mean ± SD; n = 13 sections, N = 5 ROs). (**F**) IHC images showing few GFAP+ activated Müller glial cells in iPSC-derived RPGR-deficient retinal organoids treated with RK.RPGR viral vector. (**G**) IHC image showing the presence of RHODOPSIN in the OSs but not the cell bodies of RPGR-deficient photoreceptors, following RPGR gene supplementation.
Scale bars: 25μm (**A**, **D**, **E-G** and insets). Abbreviations: OLM, outer limiting membrane; ONL, outer nuclear layer; OS, outer segment; RO, retinal organoid.

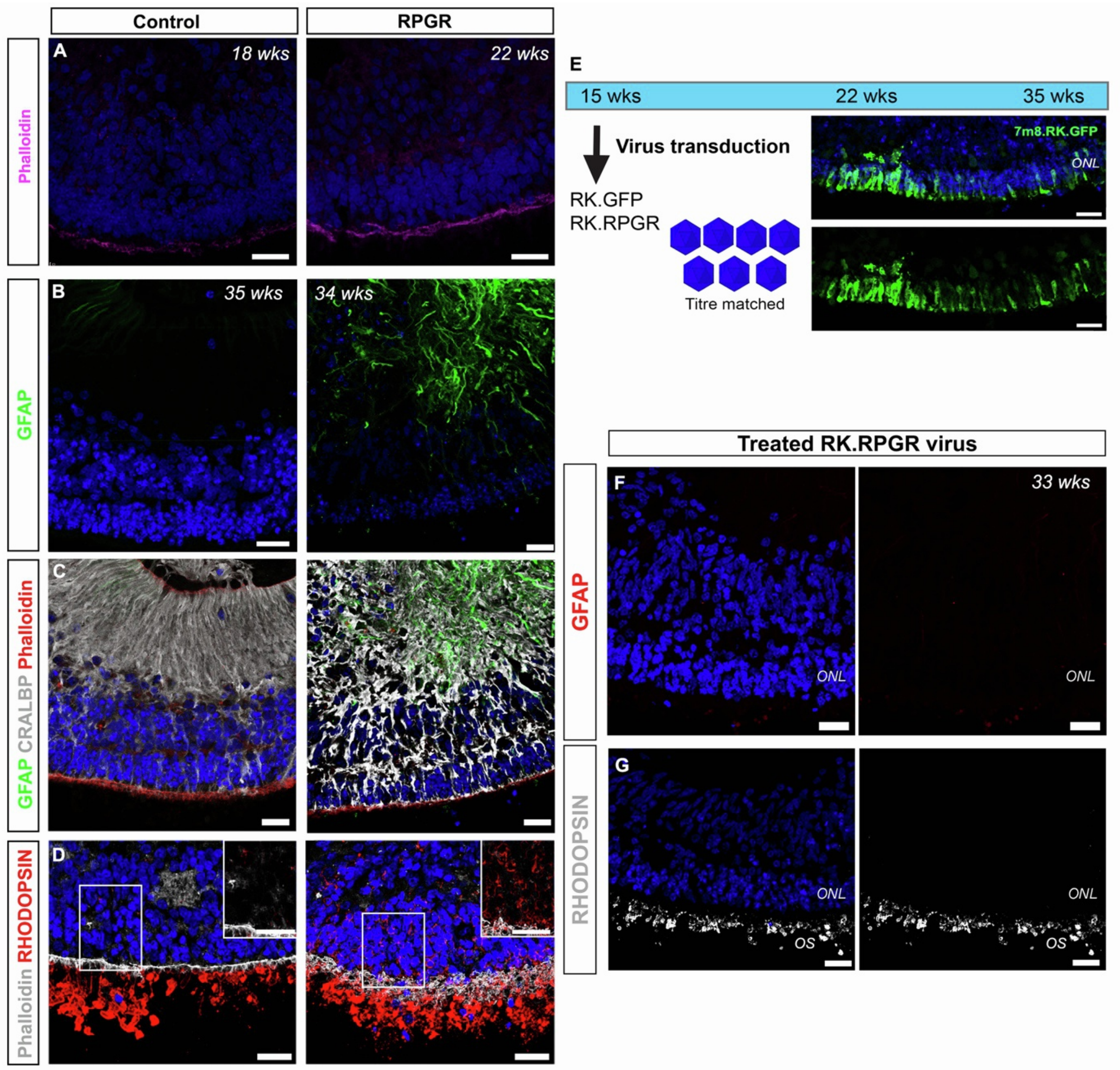
Control
RPGR
A
18 wks
22 wks
Phalloidin
B
35 wks
34 wks
GFAP
C
GFAP CRALBP Phalloidin
D
Phalloidin RHODOPSIN
E
15 wks
22 wks
35 wks
Virus transduction
RK.GFP
RK.RPGR
Titre matched
7m8.RK.GFP
ONL
Treated RK.RPGR virus
F
33 wks
GFAP
ONL
ONL
G
RHODOPSIN
ONL
ONL
OS
OS

Supplemental Video File 1. 3view EM movie through the segment region of an ALT grown retinal organoid, *related to Figure 5.*
3view 3D reconstruction of 150 sections with thickness of 100 nm each. 3view sequence of backscatter EM images of hPSC-derived retinal neuroepithelia showing photoreceptor OS (magenta), CC (blue) and IS (green).

Supplemental Tables

**Supplemental Table 1. Media composition**			
Media Components/Concentrations	RMM	AOX	ALT
DMEM/F12 (Glutamax) Media	✓	✓	___
Advanced DMEM/F12 Media†	___	___	✓
N-2 Supplement	✓	✓	✓
B-27 (-Vit A) Supplement‡	___	✓	✓
Glucose concentration	17.5mM	17.5mM	25mM
Glutamax Supplement concentration	2.5mM	2.5mM	4mM
Antibiotic/Antimycotic	✓	✓	✓
Taurine	0.25mM	0.25mM	0.25mM
Retinoic Acid (*added at day 14 - 21 only)	0.5μM*	0.5μM*	0.5μM*

†contains 400mg/L AlbuMAX® II
‡contains vitamin E, vitamin E acetate, superoxide dismutase, catalase and glutathione antioxidants

**Supplemental Table 2. Details of cell lines used**		
Cell Line	Type	Source
CCE line (EK.CCE 129/SvEv)	msESC	Kind gift of E. Robertson
Crx.GFP line (B6.SJL-Tg(Crx-GFP,-ALPP)1Clc/J)	msESC	Kind gift of Y. Arsenijevic
Nrl.GFP line (B6.Cg-Tg(Nrl-EGFP)1Asw/J)	msESC	Generated in-house
H9, WA09 (WAe009-A)	hESC	WiCell Stem Cell Bank
RB2 (WIC-WA09-RB-002)	hESC	WiCell Stem Cell Bank
IMR90-4 (WISCi004-B)	hiPSC	WiCell Stem Cell Bank
RPGR1 (UCLIOO004-A)	hiPSC	Generated in-house
RPGR2 (UCLIOO0010-A-4)	hiPSC	Generated in-house
RPGR3 (UCLIOO0011-A-1)	hiPSC	Generated in-house
UCLIOO0016-A-1	hiPSC	Generated in-house

Abbreviations: msESC, mouse embryonic stem cell; hESC, human embryonic stem cell; hiPSC, human induced pluripotent stem cell.

**Supplemental Table 3. Antibodies used for immunohistochemistry**			
Antigen	Host species	Concentration used	Supplier
**Nrl**	goat	1 in 200	R & D Systems (AF2945)
**M/L Opsin**	rabbit	1 in 100	Millipore (AB5405)
**CRALBP**	mouse	1 in 500	Abcam (AB15051)
**Crx**	mouse	1 in 800	Abnova (H00001406-M02)
**Mitochondria**	mouse	1 in 200	Millipore (MAB1273)
**GFAP**	rat	1 in 300	Calbiochem (345860)
**Arrestin3**	goat	1 in 100	Novus (NBP1-37003)
**Sox2**	mouse	1 in 200	Stemlight Cell signaling technology (NEB 9092S)
**Nanog**	rabbit	1 in 200	Stemlight Cell signaling technology (NEB 9092S)
**c-Myc**	mouse	1 in 200	Stemlight Cell signaling technology (NEB 9092S)
**Oct3/4**	rabbit	1 in 200	Stemlight Cell signaling technology (NEB 9092S)
**Recoverin**	rabbit	1 in 1000	Chemicon (AB5585)
**Rhodopsin**	mouse	1 in 1000	Sigma (O4886)
**Acetylated α-tubulin**	mouse	1 in 200	Abcam (ab24610)
**ARL3**	mouse	1:100	New East Bioscience (26070)
**ARL13b**	rabbit	1:1000	Proteintech (17711)
**Rxrγ**	rabbit	1 in 200	Abcam (ab15518)
**RETGC**	rabbit	1 in 100	Gift from K. Palczewski
**N-terminal RPGR**	rabbit	1 in 1000	Novus (NBP-57905)
**C-terminal RPGR ORF15**	rabbit	1 in 100	Gift from Alan Wright
**Peripherin-2**	rabbit	1 in 1000	Gift from Gabriel Travis
**Pericentrin (PCN)**	rabbit	1 in 1000	Abcam (ab4448)
**ABCA4**	mouse	1 in 100	Abcam (ab77285)

Supplemental Table 4. Gene-specific Primers used				
Gene name	Forward Primer (5'-3')	Reverse Primer (5'-3')	Amplicon size (bp)	Probe number
CRX	caccaggctgtgccctac	tgggtcttggcaaacagtg	107	17
ABCA4	gcgtctctggctgaagatg	tcaactctcaagtccgtccag	116	1
Rhodopsin	gcctcatcgtcacccagt	tcatctatatcatgatgaacaagcag	88	84

Supplemental Experimental Procedures

Mouse ESC culture and retinal differentiation

The mouse embryonic stem cell (mESC) lines (**Table S2**) were maintained on feeder free conditions, as previously described (Osakada et al., 2009). For 3D retinal differentiation, 3 × 10^3 dissociated mESCs were resuspended in differentiation medium (GMEM containing 1.5% KSR, 0.1 mM NEAA, 1 mM pyruvate, 0.1 mM 2-mercaptoethanol) and plated into each well of a 96-well ultra low-binding U-bottom plate (Nunclon Sphera, Thermo) and incubated at 37 °C, 5% CO_2. This was defined as day 0 of differentiation culture. Growth factor-reduced Matrigel (BD Biosciences) was added to embryoid body (EB) cell aggregates on day 1 of culture to a final concentration of 2% (v/v). For whole EB (wEB) retinal differentiation, wEBs were transferred into retinal maturation medium (RMM; DMEM/F12 Glutamax containing N2 supplement and Pen/strep) at day 9, plated in 24-well low-binding plates (Corning) at a density of 6 wEBs/well and incubated at 37 °C, 5% CO_2. The media was changed every 2–3 days, with the addition of 1 mM taurine (Sigma) from day 14 onwards and 500 nM retinoic acid (RA; Sigma) from day 14 - 21 of culture only. For long term culture and media testing wEBs were maintained in either RMM, antioxidant-rich media (AOX; DMEM/F12 Glutamax containing N2 supplement, B27 supplement without RA and Pen/strep) or advanced long-term media (ALT; Advanced DMEM/F12, B27 supplement without RA, N2 supplement, 4mM glutamax, 7.5 mM glucose and AA) from day 21 of differentiation culture onwards. In addition, docosahexaenoic acid (DHA) was prepared at a 4:1 carrier ratio with fatty acid-free BSA and added at a final concentration of 50μM, with fatty acid-free BSA used as the vehicle control. Where additional components were added to AOX media the final concentration was 25 mM glucose (+Glucose) or 0.4mg/ml AlbuMAX® II (+AlbuMAX).

Human PSC culture and retinal differentiation

The human embryonic and induced pluripotent stem cell lines (**Table S2**) were maintained in feeder free conditions with E8 (Thermo Fisher) and on geltrex coated 6 well plates. Briefly, when 80% confluent hPSCs were dissociated using Versene solution for 10 minutes. PSC small clumps were collected, washed twice with PBS and resuspended in E8 media for further maintenance culture on 6 well plates. For retinal neuroepithelial differentiation human PSCs were maintained as described above until 90-95% confluent, then media without FGF (E6, Thermo Fisher) was added to the cultures for two days (D1 and 2 of differentiation) followed by a neural induction period (up to 7 weeks) in proneural induction media (PIM; Advanced DMEM/F12, MEM non-essential amino acids, N2 Supplement, 100mM Glutamine and Pen/Strep). Lightly- pigmented islands of retinal pigmented epithelium (RPE) appeared as early as week 3 in culture. Optic vesicles were formed from within the RPE region between weeks 4 and 7. During this period neuroretinal vesicles were manually excised with 21G needles and kept individually in low binding 96 well plates in retinal differentiation media (RDM; DMEM, F12, Pen/Strep and B27 without retinoic acid). At 6 wks of differentiation retinal differentiation medium was

supplemented with 10% FBS, 100uM taurine (Sigma, T4871) and 2mM glutamax and at 10 wks 1 uM retinoic acid (RA) was added (RDM+ Factors media). At 10 wks of culture vesicles were transferred to low binding 24 well plates (5 vesicles/well). At 12 wks of differentiation, media was changed again to either our standard RDM90 media, which is RDM+ Factors supplemented with N2 supplement and 0.5 uM RA or the advanced long term (ALT) media, composed of Advanced DMEM/F12, B27 without RA, N2 supplement, 4mM glutamax, 7.5 mM glucose, 100uM taurine, 0.5 uM RA and Pen/Strep. Maintenance cultures of hPSCs were feed daily and differentiation cultures were feed every 2-3 days. All representative images in the paper were from Wicell H9 ESC line, unless stated otherwise. To further validate the system, we assessed segment formation in a second ESC line (H9Rb2, Wicell), an iPSC control line (IMR90-4, Wicell) and several hiPSC lines from patients (**Table S2**). In all cell lines tested (N>5 cell lines), an enhanced brush border was observed (see **Figures 2**, **3**, **6A** and **Figure S7**).

Generation of human iPSC lines

Following informed consent, peripheral blood was taken from individuals with confirmed XLRP3 inherited retinal degeneration. The study followed the tenets of the Declaration of Helsinki and was approved by the Moorfields and Whittington Hospitals' local Research Ethics Committees and the NRES Committee London Riverside Ethics Committee (REC 11/H0721/13).

On day 0, 2x 10^6 PBMCs were defrosted and added to 10ml of prewarmed (37°C) Stemspan 3000 with Penicillin/ Streptomycin (SS Medium). Following centrifugation at 300g for five minutes, the pellet of cells was resuspended in Expansion Medium (EM) and plated in one well of a 12-well plate. On day 3, cells were collected, centrifuged, resuspended in 2ml of EM Medium and plated in one well of a 12-well plate. Cells were left to expand in expansion medium for a further 3 days prior to nucleofection (6 days in total) prior to reprograming. On day 6, medium containing the cells was collected into a 15ml falcon tube. 200,000 cells were transferred into a new tube with 12ml of PBS and centrifuged at 300g for 5 minutes. Lonza P3 Nucleofection supplement was added to P3 buffer (P3 buffer), from P3 Primary Cell 4D-Nucleofector™ X Kit L (Lonza™), according to manufacturer instruction (20µl/electroporation reaction). Addgene episomal plasmids pCXLE-hUL [pCXLE-hUL was a gift from Shinya Yamanaka (Addgene plasmid #27080; http://n2t.net/addgene:27080; RRID:Addgene_27080)], pCXLE-hSK [pCXLE-hSK was a gift from Shinya Yamanaka (Addgene plasmid #27078; http://n2t.net/addgene:27078; RRID:Addgene_27078)] and pCXLE-hOCT3/4-shp53-F [pCXLE-hOCT3/4-shp53-F was a gift from Shinya Yamanaka (Addgene plasmid #27077; http://n2t.net/addgene:27077; RRID:Addgene_27077)](Okita et al., 2007) were added to P3 buffer (0.33 µg each). Following centrifugation, the cell pellet was resuspended in the P3 buffer containing Yamanaka's plasmids. Cells were then transferred to the cuvette strip (Lonza™) and electroporated using program EO-115 of Amaxa 4D-Nucleofector™ System (Lonza). After electroporation cells were kept at room temperature for 5 minutes. 80µL of Roswell Park Memorial Institute (RPMI) 1640 (Gibco™) was added to the cuvette strip and it was placed at 37°C for 10 minutes. Cells were then added to 1 well of a 6-well plate (Corning®Costar), previously coated with Geltrex (1:100), in 2ml of EM. Cells were kept in EM until day 8. From day 8 to day 10 cells were fed daily with a 1:1 mix of Essential 8™ Medium (E8 Medium; Invitrogen) and EM. After day 10 cells were fed daily with E8 Medium. Retinal differentiation of patient iPSC cells was performed as described above.

Production and use of recombinant AAV viral vector

Both pD10/RK*promoter-GFP* and pD10/RK*promoter-RPGR,* construct containing AAV-2 inverted terminal repeat (ITR) were used to generate AAV7m8.RK.GFP and RK.RPGR viral vector. Recombinant AAV2/2 serotype particles were produced through a previously described triple transient transfection method HEK293T cells (Nishiguchi et al., 2015). AAV7m8 serotype was bound to an AVB Sepharose column (GE Healthcare) and eluted with 50 mM Glycine pH2.7 into 1 M Tris pH 8.8. Vectors were washed in 1 × PBS and concentrated to a volume of 100–150 µl using Vivaspin 4 (10 kDa) concentrators. Viral genome titres were determined by quantitative real-time PCR using a probe-based assay binding the SV40 poly-adenylation signal. Amplicon-based standard series of known amounts were used for sample interpolation. Final titres were expressed as vg/mL.

SV40 Forward primer: 5'-Agcaatagcatcacaaatttcacaa-3'.

SV40 Reverse primer: 5'-AGATACATTGATGAGTTTGGACAAAC-3'.

SV40 Probe: FAM-5'-AGCATTTTTTTCACTGCATTCTAGTTGTGGTTTGTC-3'-TAMRA. Retinal organoids were infected at 15-18 wks with $3x10^{11}$ viral particles per organoid, with an estimated gMOI of $6x10^{5}$.

Immunohistochemistry and immunocytochemistry

hPSC-derived retinal organoids (n>20 NRVs; N=3 independent experiments) were used for assessments of ALT media and OS formation as described in the main manuscript. Retinal organoids were collected either unfixed (RPGR antibodies) or fixed for 1 hour in 4% paraformaldehyde (PFA) and embedded in OCT (RA Lamb). Cryosections were cut (12-14 µm thick) and all sections were collected for analysis. For immunohistochemistry, sections were blocked in 5% goat or donkey serum and 1% bovine serum albumin in PBS. Primary antibody (**Table S3**) was incubated overnight at 4°C. Sections were incubated with secondary antibody for 2 hrs at RT, washed and counter-stained with DAPI (Sigma-Aldrich). Alexa fluor 488, 546 and 633 secondary antibodies (Invitrogen-Molecular Probes) were used at a 1:500 dilution.

For immunocytochemistry of whole retinal organoids (3D view) a clearing protocol was performed. Briefly, NRVs were fixed for 1 hour in 4% PFA. Samples were blocked as above, including 0.3% Triton X-100 in PBS, and primary antibody was incubated overnight at 4°C. Samples were incubated with secondary antibody and DAPI overnight at 4°C. Samples were dehydrated in a graded ethanol series (30, 50, 70, 80, 96, and 2 x 100% ethanol in PBS), and transferred into clearing solution (2 parts benzylbenzoate (Sigma-Aldrich):1 part benezylalcohol (Sigma-Aldrich) for 20 min in the dark. Secondary antibodies were used at a 1:300 dilution.

Confocal image acquisition and quantification

Images were acquired by confocal microscopy (Leica DM5500Q). A series of XY optical sections, approximately 0.8µm apart, throughout the depth of the section were taken and built into a stack to give a projection image. LAS AF image software was used.

To determine the organisation of photoreceptors within mESC-derived 3D retinal differentiation cultures, cryoembedded EBs (x6 EBs/sample) were sectioned (18 µm), stained for Recoverin and mounted. As the quantity of organised retinal regions can vary greatly between embryoid bodies and batches of differentiation we analysed at least 12 EBs over multiple independent differentiations (n>3) using several mouse ES cell lines (N=3), to quantify the presence of organised ONL-like photoreceptor layers. Every sixth section through the EBs was examined for the presence of at least one region of Recoverin+ photoreceptors, if this was not present the section was classed as non-retinal (Figure S1A,E). If Recoverin+ photoreceptors were present, the sections were further examined for the presence of an ONL-like layer using DAPI (Figure S1F,G; ONL-like layer indicated with white dashed lines). Recoverin+ segment structures extending from the ONL-like layer could also be observed, further confirming a region of organised photoreceptors (Figure S1B,C,F,G; white arrow heads). If Recoverin+ cells were present but there was no indication of any ONL-like regions observed with DAPI, this section was classed as disorganised photoreceptors (Figure S1D,H). All analysis was performed blinded.

Human retinal organoids were cryoembedded before sectioning and mounting. PHPR2+ OS structures were located using epifluorescence illumination and measures were taken from 10 regions of the neuroepithelia in 10 images using FiJi software. To accurately measure segment length, only regions showing aligned sagittal sections through the segments were used, as determined by Phalloidin expression in the OLM and Mitochondria staining in the ISs.

For pixel intensity quantification a threshold was set that separated the fluorescent signal from background. All images were analysed using the same threshold value and presented as the percentage of positive pixels above threshold. All other aspects of imaging were kept consistent. For the quantification of RPGR and RHODOPSIN, the the CC region and the ONL were selected for analysis, respectively. For the quantification of GFAP, the whole image was used. Image analysis was performed blinded.

Ultrastructural analysis

For ultrastructural analysis, mESC-derived EBs or hPSC-derived retinal organoids were fixed in 3 % glutaraldaehyde and 1 % paraformaldehyde in 0.08M sodium cacodylate-HCl (pH 7.4), for at least 12 hrs at 4ºC. The specimens were washed with 2.5% glutaraldehyde in 0.1M cacodylate-HCl buffer (pH 7.4) and post-fixed in 1 % aqueous solution of osmium tetroxide for 2.5 hrs in the dark. The specimens were then dehydrated by an ascending ethanol series (50 - 100 %, 10 mins per step with rotation) with additional 2 x 10 mins 100 % ethanol and 3 x 10 mins 1,2-epoxypropane steps. The specimens were left in a 50:50 mixture of 1,2-epoxypropane and araldite for a minimum of 3 hrs with rotation, followed by 5 hrs in fresh araldite and embedded and cured at 60 ºC for 48 hrs. Semithin (0.7 µm) and ultrathin (70 - 100 nm) sections were cut using a Leica ultracut S microtome fitted with an appropriate diamond knife (Diatome histoknife Jumbo or Diatome Ultrathin). Semithin sections were stained with 1 % toluidine blue and imaged using a Leitz Diaplan microscope fitted with a Leica digital camera (DC 500). Ultrathin sections were collected on copper formvar coated grids (100 mesh, Agar Scientific) and imaged both with and without 1% lead citrate contrast-staining on a JEOL 11010 TEM operating at 80V. Images were acquired with a Gatan Orius camera and montaged using Digital Micrograph software. Several montaged images were stitched together using Adobe Photoshop elements 12 software and cropped to produce the final figure panels.

For scanning electron microscopy 1 and 2 µm thick semithin sections were cut and dried onto 20 mm glass coverslips, etched for 30 minutes in potassium methoxide (Sigma Cat 60402-250 ml), rinsed twice for five minutes in methanol followed by hexamethyldisilazane (HMDS) and air drying. Dried specimens were coated with 1.5 nm of platinum and imaged in a Zeiss Sigma FESEM operating at 3 – 5kV.

Serial Block-Face Scanning Electron Microscopy (3view EM)

hPSC-derived retinal organoids were fixed in 3% glutaraldehyde and 1% paraformaldehyde in 0.08 M sodium cacodylate –HCl buffer, pH 7.4, and then en bloc stained with osmium ferricyanide-thiocarbohydrazide-osmium, uranyl acetate and Walton's lead citrate with two modifications. First, the osmium concentration was reduced to 1% and, second, graded alcohols (50, 70, 90, 3 x 100%) and propylene oxide were used instead of acetone to dehydrate specimens for infiltration and curing overnight at 60°C in Durcupan ACM resin. Specimens were then superglued to aluminium pins and trimmed to place the region of interest within a 0.5 x 0.5 x 0.4 mm mesa and sputter coated with 5 nm gold palladium. Stacks of backscatter electron micrographs were automatically acquired using a Gatan 3 view system working in conjunction with a Zeiss Sigma field emission scanning electron microscope working in variable pressure mode at a chamber pressure of 9 Pa and 4 kV. At a standard magnification of x1000 and a pixel resolution of 4096 x 4096, the total area sampled measured 255.4 µm2 on x and y and, depending on the number of 100 nm thick sections sampled, between 67 and 150 µm on z. The resulting stacks were normalised for contrast and brightness and then converted to TIFF images in Digital Micrograph software prior to importation into Amira 5.3.3 software for semi-automated segmentation and presentation.

Quantitative PCR

50 ng of cDNA was loaded per well of 96-well plate (Life Technologies Ltd., UK) mixed with 2x Fast Start TaqMan® Probe Master Mix (Roche Ltd., UK), gene-specific forward and reverse primers at a final 900 nM concentration and an appropriate hydrolysis probe binding to the amplified region at a final concentration of 250 nM (Roche Diagnostics Ltd., UK), all dissolved in DNase and RNase free water up to 20 µl final volume. Each cDNA sample was run in triplicate. The reactions were then run on an ABI Prism 7900HT Fast Real-time Sequence Detection System (Applied Biosystems Ltd., UK) equipped with SDS 2.2.2 software for amplication results analysis. From amplification curves Ct values were obtained for each sample. Expression levels were normalized to photoreceptor specific *Crx* (*Crx* gene) mRNA levels for each sample to assess relative expression of particular genes in different experimental conditions. Cycling conditions were as follows 40 cycles of 95°C for 30 sec. and 60°C for 1 minute. **Table S4** contains the list of gene-specific primer sequences used.

Flow cytometry analysis

Mouse ESC-derived retinal vesicles were dissociated at various time points of culture into a single cell suspension using a papain-based Neurosphere Dissociation Kit (Miltenyi Biotec, 130-095-943). Cells were counted and resuspended in 1% Bovine Serum Albumin (in PBS) to a concentration of $1x10^7$ cells per mL, and CD73 antibody staining (Miltenyi Biotec, 130-103-054) was performed in 100µL aliquots and incubated for 30 minutes at 4°C at a dilution of 1:100. Cells were washed once in 1X Binding Buffer and resuspended in PBS. SYTOX Blue (Invitrogen) was then added to the samples at a final concentration of 1µM for 5 minutes at room temperature before analysis. Cells were analysed using FACSDiva software. Background fluorescence was measured using unstained cells and single-stained controls were used to set gating parameters between positive and negative populations (see **Figure S2**). Small debris, cell fragments and aggregates were excluded from analysis on the basis of live-dead dye fluorescence followed by forward and side scatter (measuring cell size and granularity, respectively).

Statistical analysis

All means are presented ± SD (standard deviation), unless otherwise stated; N, number of independent differentiations or experiments performed; n, number of images, sections, EBs or organoids examined, where appropriate. For quantification assessment by Flow cytometry statistical analysis is based on at least three independent experiments. Statistical significance was assessed using Graphpad Prism 6 software and denoted as $*p < 0.05$, $**p < 0.01$, $***p < 0.001$ and $****p < 0.0001$. Appropriate statistical tests were applied including ANOVA with relevant post hoc correction for multiple comparisons; Kruskal-Wallis with Dunn's multiple comparisons test; two-tailed Mann-Whitney test.

Supplemental References

Nishiguchi, K.M., Carvalho, L.S., Rizzi, M., Powell, K., Holthaus, S.-M. kleine, Azam, S.A., Duran, Y., Ribeiro, J., Luhmann, U.F.O., Bainbridge, J.W.B., et al. (2015). Gene therapy restores vision in rd1 mice after removal of a confounding mutation in Gpr179. Nature Communications *6*, 6006.

Okita, K., Ichisaka, T., and Yamanaka, S. (2007). Generation of germline-competent induced pluripotent stem cells. Nature *448*, 313–317.

Osakada, F., Ikeda, H., Sasai, Y., and Takahashi, M. (2009). Stepwise differentiation of pluripotent stem cells into retinal cells. Nature Protocols *4*, 811–824.
